# Strategies for post–cardiac surgery acute kidney injury prevention: A network meta-analysis of randomized controlled trials

**DOI:** 10.3389/fcvm.2022.960581

**Published:** 2022-09-27

**Authors:** Jia-Jin Chen, Tao Han Lee, George Kuo, Yen-Ta Huang, Pei-Rung Chen, Shao-Wei Chen, Huang-Yu Yang, Hsiang-Hao Hsu, Ching-Chung Hsiao, Chia-Hung Yang, Cheng-Chia Lee, Yung-Chang Chen, Chih-Hsiang Chang

**Affiliations:** ^1^Department of Nephrology, Chang Gung Memorial Hospital, Taoyuan, Taiwan; ^2^Chansn Hospital, Taoyuan, Taiwan; ^3^Department of Nephrology, Kidney Research Center, Linkou Chang Gung Memorial Hospital, Taoyuan, Taiwan; ^4^Department of Surgery, National Cheng Kung University Hospital, College of Medicine, National Cheng Kung University, Tainan, Taiwan; ^5^Department of Anesthesiology, Mackay Memorial Hospital, Taipei, Taiwan; ^6^Department of Cardiothoracic and Vascular Surgery, Chang Gung Memorial Hospital, Linkou Medical Center, Taoyuan, Taiwan; ^7^Department of Nephrology, New Taipei Municipal TuCheng Hospital, New Taipei City, Taiwan; ^8^Department of Cardiology, Chang Gung Memorial Hospital, Taoyuan, Taiwan

**Keywords:** acute kidney injury, cardiac surgery, dexmedetomidine, natriuretic peptide, remote ischaemic preconditioning

## Abstract

**Objects:**

Cardiac surgery is associated with acute kidney injury (AKI). However, the effects of various pharmacological and non-pharmacological strategies for AKI prevention have not been thoroughly investigated, and their effectiveness in preventing AKI-related adverse outcomes has not been systematically evaluated.

**Methods:**

Studies from PubMed, Embase, and Medline and registered trials from published through December 2021 that evaluated strategies for preventing post–cardiac surgery AKI were identified. The effectiveness of these strategies was assessed through a network meta-analysis (NMA). The secondary outcomes were prevention of dialysis-requiring AKI, mortality, intensive care unit (ICU) length of stay (LOS), and hospital LOS. The interventions were ranked using the *P*-score method. Confidence in the results of the NMA was assessed using the Confidence in NMA (CINeMA) framework.

**Results:**

A total of 161 trials (involving 46,619 participants) and 53 strategies were identified. Eight pharmacological strategies {natriuretic peptides [odds ratio (OR): 0.30, 95% confidence interval (CI): 0.19–0.47], nitroprusside [OR: 0.29, 95% CI: 0.12–0.68], fenoldopam [OR: 0.36, 95% CI: 0.17–0.76], tolvaptan [OR: 0.35, 95% CI: 0.14–0.90], N-acetyl cysteine with carvedilol [OR: 0.37, 95% CI: 0.16–0.85], dexmedetomidine [OR: 0.49, 95% CI: 0.32–0.76;], levosimendan [OR: 0.56, 95% CI: 0.37–0.84], and erythropoietin [OR: 0.62, 95% CI: 0.41–0.94]} and one non-pharmacological intervention (remote ischemic preconditioning, OR: 0.76, 95% CI: 0.63–0.92) were associated with a lower incidence of post–cardiac surgery AKI with moderate to low confidence. Among these nine strategies, five (fenoldopam, erythropoietin, natriuretic peptides, levosimendan, and remote ischemic preconditioning) were associated with a shorter ICU LOS, and two (natriuretic peptides [OR: 0.30, 95% CI: 0.15–0.60] and levosimendan [OR: 0.68, 95% CI: 0.49–0.95]) were associated with a lower incidence of dialysis-requiring AKI. Natriuretic peptides were also associated with a lower risk of mortality (OR: 0.50, 95% CI: 0.29–0.86). The results of a sensitivity analysis support the robustness and effectiveness of natriuretic peptides and dexmedetomidine.

**Conclusion:**

Nine potentially effective strategies were identified. Natriuretic peptide therapy was the most effective pharmacological strategy, and remote ischemic preconditioning was the only effective non-pharmacological strategy. Preventive strategies might also help prevent AKI-related adverse outcomes. Additional studies are required to explore the optimal dosages and protocols for potentially effective AKI prevention strategies.

## Introduction

Acute kidney injury (AKI) is a common complication in hospitalized patients, with an incidence of 7–18% among inpatients (1). Among patients who have undergone surgery, those who have undergone cardiac surgery are at the highest risk of postoperative AKI (2–4). The incidence of AKI among patients who have undergone cardiac surgery ranges from 20 to 70% depending on the type of cardiovascular surgery and the definition of AKI used (5, 6). Post–cardiac surgery AKI is associated with increased risks of short-term and long-term mortality, increased incidence of chronic kidney disease, and higher medical resource utilization (6–10).

The mechanisms underlying post–cardiac surgery AKI are complex and may include intrarenal hemodynamic perturbation, iron metabolism, an increase in the production of reactive oxygen species and an increase in free hemoglobin levels, the activation of an inflammatory and immunological cascade, and factors specific to cardiovascular operations (cardiopulmonary bypass [CPB], aortic cross-clamping, ischemic or reperfusion injury, and frequent exogenous blood transfusion) (6, 10–14). Both perioperative pharmacological and non-pharmacological AKI prevention strategies have been proposed (4, 6). In 2018, the Acute Disease Quality Initiative (ADQI) Group recommended the avoidance of glucose variability; the use of balanced crystalloid solutions, remote ischemic preconditioning (RIPC), dexmedetomidine, and the KDIGO AKI prevention bundle; and limited use of blood transfusions as strategies to prevent post–cardiac surgery AKI (6). However, several new preventive strategies were not thoroughly discussed in the aforementioned ADQI consensus statement. Furthermore, numerous researchers have adopted their own definitions of AKI or have used criteria that are no longer widely accepted. Moreover, non-pharmacological interventions (other than RIPC) have not been systematically examined in meta-analyses (15–17). The most recent network meta-analysis (NMA), which was published in 2018, only enrolled studies published before 2016 that focused on pharmacological strategies, and the study did not evaluate AKI-related adverse outcomes (extended ICU and hospital length of stay [LOS] and mortality) (18).

To address the aforementioned shortcomings of previous relevant studies, we conducted this updated NMA analyzing the effectiveness of various pharmacological and non-pharmacological AKI prevention strategies as well as the effectiveness of such strategies in preventing dialysis-requiring AKI and AKI-related adverse outcomes, including extended hospital length of stay (LOS), ICU LOS, and mortality.

## Materials and methods

### Literature search strategy

We performed this NMA in accordance with the Preferred Reporting Items for Systematic Reviews and Meta-Analyses Extension for NMAs (Supplementary Table 1) and registered our study design and protocol in PROSPERO (CRD42021278036).

Two authors (J-JC and TL) independently searched for studies published prior to November 3, 2021, in PubMed, Medline, and Embase. The search strategies targeted published clinical trials that compared the efficacy of different interventions in preventing post–surgery AKI in patients who had undergone cardiac surgery. A detailed description of the search strategy and the results of the search process are provided in Supplementary Table 2. Review articles and meta-analyses were excluded from our analysis, but their references were screened for relevant studies. Two authors also searched ClinicalTrials.gov to identify completed but unpublished trials by using the keywords “acute kidney injury” and “cardiac surgery,” limiting our search to clinical trials involving adult populations. A third reviewer (G. Kou) helped resolve disagreements between the two aforementioned authors (J-JC and TL) and reviewed the search strategy.

### Study eligibility criteria

The two authors who conducted the search (J-JC and TL) independently examined the titles and abstracts of the identified studies, and articles were excluded upon initial screening if their titles or abstracts indicated that they were clearly irrelevant to the objective of the current study. The full texts of the relevant articles were reviewed to assess the eligibility of the studies. A study was included if it (1) enrolled adults who had undergone cardiac surgery, including coronary artery bypass grafting (CABG), heart valve surgery, or aortic surgery; (2) was a randomized controlled trial (RCT) with a parallel design; (3) assigned patients to at least two intervention arms to compare the efficacy of preventive strategies; and (4) reported either outcomes related to AKI (or dialysis-requiring AKI) or acute renal failure (diagnosed according to the Kidney Disease: Improving Global Outcomes [KDIGO]; Acute Kidney Injury Network [AKIN]; and Risk, Injury, Failure, Loss of kidney function, and End-stage kidney disease [RIFLE] criteria and others). The third reviewer (G. Kou) was consulted to help establish a consensus in case of any disagreement regarding a study’s eligibility. Surgical techniques and procedures (on-pump or off-pump CABG, cerebral protection devices, and CPB-related techniques) were not evaluated in the current study. The study population of interest comprised patients who had undergone open cardiac surgery; therefore, studies that enrolled patients who had undergone endovascular procedures or who had received percutaneous interventions were excluded.

### Data extraction and outcomes

Two investigators (J-JC and TL) independently classified the interventions and used a standardized data abstraction form to extract data related to the study design (randomization and double-blind or non-blinded designs), participant demographics (age and gender), interventions, types of cardiac surgery (CABG, heart valve surgery, or aortic surgery), and definitions of AKI (KDIGO, AKIN, RIFLE, or others). Data related to the primary outcome, namely post–cardiac surgery AKI, were also extracted. Data on the secondary outcomes, namely dialysis-requiring AKI, and AKI-related adverse outcomes [mortality and intensive care unit (ICU) and hospital LOS (in days)], were also extracted.

### Data synthesis and analysis

The characteristics of the enrolled participants (mean age, percentage of female participants, and cardiac operation type), definition of AKI, and interventions in the included studies were extracted from the corresponding articles (Supplementary Table 3). We identified and classified 54 interventions. For three-arm studies evaluating different doses of erythropoietin (EPO) (19) or dexmedetomidine (20), four-arm studies evaluating different doses of THR-184 (21) or ABT-719 (α-melanocyte-stimulating hormone analog), and five-arm studies evaluating different durations of RIPC (22), we combined the results from intervention arms with different doses/durations into a single arm. Various synthetic replacement fluids (6% hydroxyethyl starch [HES; 130/0.4], 6% HES [7.2% NaCl, HES 200/0.5], and succinylated gelatin [4%]) were categorized as synthetic colloids. Various chloride-poor crystalloid fluids (e.g., Ringer’s lactate solution, Plasma-Lyte, and Hartmann’s solution) were categorized as balanced fluids. One RCT compared two interventions: chloride-liberal and chloride-restrictive intravenous fluids (23). In the chloride-restrictive fluid arm, multiple colloid and crystalloid fluids were used (HES [130/0.4] in balanced colloid solution [Volulyte] and balanced salt crystalloid solution [PlasmaLyte A]). Therefore, we considered this study as the control arm compared with a chloride-restrictive fluid strategy. Normal saline as a replacement fluid and a chloride-liberal fluid strategy were considered as control arms.

To evaluate the effectiveness of various strategies in preventing AKI, dialysis-requiring AKI, and mortality, we calculated odds ratios (ORs) to pool binary outcomes. To analyze ICU and hospital LOS, we extracted the mean and the standard deviation of hospital and ICU LOS within each intervention arm. For studies that reported ICU and hospital LOS in terms of medians and interquartile ranges, we extracted the relevant data and translated them into means and standard deviations according to the Cochrane guidelines (24). Heterogeneity was examined using *I*^2^ values (with a value of ≥ 50% indicating substantial heterogeneity). We used the statistical package *netmeta* in R (version 4.0.2) to perform a frequentist NMA with a random-effects model (25). All pairwise comparisons were summarized in a league table. The *P*-score method is used to measure the probability that an intervention is superior to a competing intervention in terms of a particular outcome (26, 27). Each intervention was assigned a *P*-score ranging from 0 to 1; an effective intervention with a higher *P* score was determined to be more effective than an intervention with a lower *P* score. We performed a sensitivity analysis to assess the robustness of our results, in which studies that did not use international AKI criteria (those other than the RIFLE, AKIN, or KDIGO criteria) were excluded. We also performed a sensitivity analysis in which studies with some or high risk of bias were excluded and another sensitivity analysis in which studies with small numbers of participants (<50) were excluded. In addition, we performed a subgroup analysis to explore heterogeneity in the outcomes of interest among patients who underwent (1) heart surgery (CABG or valve surgery or combination of both) and (2) aortic surgery. The subgroup analysis only included studies that used international AKI criteria. Additional subgroup analyses were performed to explore heterogeneity in the outcomes of interest among participants with (1) preserved renal function (serum creatinine ≤ 1.2 mg/dL or an estimated glomerular filtration rate ≥ 60 mL/min/1.73 m^2^) and (2) impaired renal function (serum creatinine > 1.2 mg/dL or an estimated glomerular filtration rate < 60 mL/min/1.73 m^2^). Studies without baseline mean or median renal function test data were excluded from this subgroup analysis. A two-tailed *p*-value of < 0.05 was considered statistically significant.

### Risk-of-bias and quality assessments

We used the revised Cochrane Risk-of-Bias tool for randomized trials (RoB 2) (28) to assess the quality of the included RCTs. Any disagreements regarding the risk of bias between the two main investigators (J-JC and TL) were resolved through discussion with the third investigator (G. Kou). Inconsistency of current NMA was evaluated using a design-by-treatment interaction model and node-splitting model (29, 30). Small-study effect bias was examined using a funnel plot, and publication bias was measured using the Egger test. The results of the bias assessment were visualized using a risk-of-bias plot (31). The overall confidence in and quality of the results of NMA were evaluated using the Confidence in NMA (CINeMA) framework (30).

## Results

### Search results

The flowchart and search strategy used in the literature search are detailed in Supplementary Figure 1 and Supplementary Table 2. We identified 490, 656, and 281 studies from PubMed, Embase, and Medline, respectively, as well as two meta-analyses from the Cochrane Library and 45 completed trials registered on ClinicalTrials.gov. After duplicates and registered trials without available data for the outcomes of interest were removed, 1382 studies met the search criteria and were screened by title or abstract. After screening, the full texts of 210 potentially eligible studies were examined. Moreover, we identified 42 additional eligible studies by screening references in other meta-analyses and review articles. Therefore, we reviewed the full texts of a total of 252 potentially eligible studies. Ultimately, 161 studies were included in our study (19–23, 32–187). Some studies were excluded because the enrolled participants had already developed AKI (*n* = 5), had already received renal replacement therapy (*n* = 3), had undergone non-cardiac surgery (*n* = 5), or were not adults (*n* = 8). Studies that were not RCTs (*n* = 6), focused on surgical techniques or procedures (*n* = 23), or did not report the incidence of AKI-related outcomes or events (*n* = 28) were also excluded. Eight two-arm studies that could not connect to other studies in the network were also excluded (188–194). One study on the management of postoperative cardiogenic shock that compared vasopressin and norepinephrine, which was identified from a review article (6), was excluded for the same reason (195).

### Study characteristics

The 161 studies involved a total of 46,619 participants with a mean age of 66.9 years (range: 43–78.2 years), and 31.7% of the participants (excluding those in one study that did not report baseline demographic characteristics) were female (77). The studies were published between 2000 and 2021 and had sample sizes ranging from 10 to 3647. Of the included studies, 142, 13, and 7 enrolled participants who had undergone pure heart surgery (CABG or cardiac valve surgery), pure aortic surgery (thoracic or abdominal), and a combination of heart and aortic surgery, respectively. A total of 90 studies defined AKI according to international AKI criteria (AKIN, RIFLE, or KDIGO); the remaining 71 studies used other definitions or did not report the definition of AKI or renal failure employed therein. From the 161 RCTs (which comprised 151 two-arm studies, 5 three-arm studies, 4 four-arm studies, and 1 five-arm study), a total of 54 strategies (including controls) were identified: ABT-719 (AP214 acetate), acetaminophen, albumin, amustaline-treated red blood cells, antioxidants (a combination of omega 3 polyunsaturated fatty acids, vitamin C, and vitamin E), aprotinin, autologous platelet-rich plasma, balanced solutions, bicarbonate, chloride-restrictive fluids, calorie-restricted diets, carvedilol, control, curcumin, cyclosporin, dexmedetomidine (low and high doses), dobutamine, dopamine, EA-230, EPO (low and high doses), ethyl pyruvate, fenoldopam, fenoldopam with N-acetyl cysteine (NAC), furosemide, forced diuresis (RenalGuard system), intensive insulin therapy, KDIGO AKI prevention bundles, L-amino acids, levosimendan, mannitol, methylxanthines, minocycline, NAC, NAC with carvedilol, natriuretic peptides, nifedipine, nitroprusside, prophylactic transfusions, restrictive transfusions, rasburicase, RIPC (different durations and preoperative or postoperative), selenium, small interfering RNA (siRNA, teprasiran), spironolactone, statins, steroids, stroke volume variation (SVV)–guided fluid therapy, synthetic colloids, THR-184 (a bone morphogenetic protein-7 agonist), tolvaptan, vitamin C, vitamin D, vitamin E with allopurinol, and volume replacement therapy. The characteristics and study designs of the selected RCTs are summarized in Supplementary Table 3.

### Network meta-analysis outcome: Acute kidney injury prevention

**FIGURE 1:**
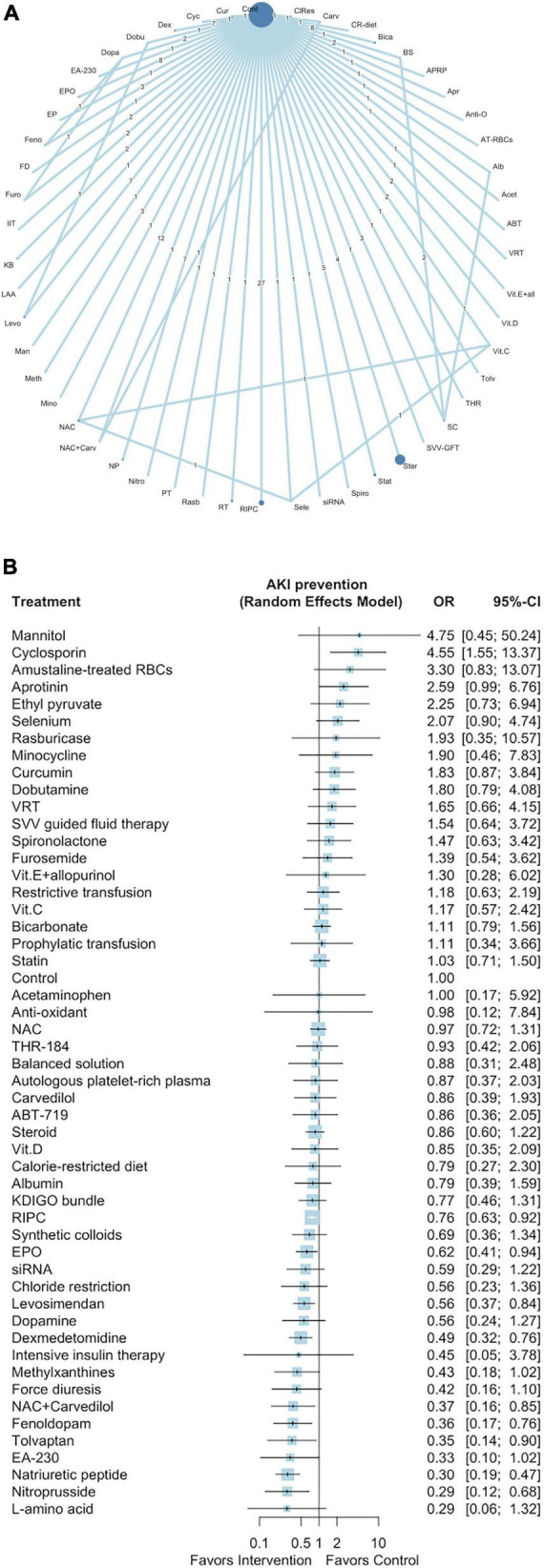
Network plot of eligible comparisons among interventions for AKI prevention **(A)**, Forest plot of network meta-analysis for AKI prevention **(B)**. In the network plot, the size of each node indicates the number of randomized allocated participants. ABT, ABT-719; Acet, acetaminophen; Alb, albumin; AT-RBCs, amustaline-treated RBCs; Anti-O, anti-oxadants; Apr, aprotinin; APRP, autologous platelet-rich plasma; BS, balanced solution; Bica, bicarbonate; CR-diet, calorie-restricted diet; Carv, carvedilol; ClRes, chloride restriction; Cont, control; Cur, curcumin; Cyc, cyclosporin; Dex, dexmedetomidine; Dobu, dobutamine; Dopa, dopamine; EP, ethyl pyruvate; EPO, erythropoietin; Feno, fenoldopam; FD, forced diuresis; Furo, furosemide; IIT, intensive insulin therapy; KB, KDIGO bundle; LAA, L-amino acid; Levo, levosimendan; Man, mannitol; Meth, methylxanthines; Mino, minocycline; NAC, *N*-acetyl cysteine; NP, natriuretic peptide; Nitro, nitroprusside; PT, prophylactic transfusion; Rasb, rasburicase; RT, restrictive transfusion; RIPC, remote ischemic preconditioning; Sele, selenium; Spiro, spironolactone; Stat, statin; Ster, steroid; SVV-GFT, stroke volume variation guided fluid therapy; SC, synthetic colloids; THR, THR-184; Vit.C, vitamin C; Vit.D, vitamin D; Vit.E + all, vitamin E + allopurinol; VRT, volume replacement therapy.

Figure 1A presents a network plot of the 52 interventions (nodes), 43,786 participants, and 149 direct comparisons of interventions for preventing post–cardiac surgery AKI from the 141 selected studies. Eight pharmacological interventions (ordered according to the P score: natriuretic peptide [OR: 0.30, 95% CI: 0.19–0.47], nitroprusside [OR: 0.29, 95% CI: 0.12–0.68], fenoldopam [OR: 0.36, 95% CI: 0.17–0.76], tolvaptan [OR: 0.35, 95% CI: 0.14–0.90], NAC with carvedilol [OR: 0.37, 95% CI: 0.16–0.85], dexmedetomidine [OR: 0.49, 95% CI: 0.32–0.76], levosimendan [OR: 0.56, 95% CI: 0.37–0.84], and EPO [OR: 0.62, 95% CI: 0.41–0.94]) and one non-pharmacological intervention (RIPC, OR: 0.76, 95% CI: 0.63–0.92) were associated with a lower incidence of post–cardiac surgery AKI (Figure 1B). One intervention was associated with an increased incidence of AKI (cyclosporin [OR: 4.55, 95% CI: 1.55–13.37]). The results of the NMA and direct pairwise comparisons are summarized in the league table in Supplementary Document 1. Natriuretic peptides were more effective than levosimendan, EPO, and RIPC (Supplementary Document 1). We noted mild heterogeneity among the included studies (*I*^2^ = 41.4%).

We used the *P*-score method to rank the 52 interventions (Supplementary Table 4). The results of the Egger test and the funnel plot revealed small-study bias (Egger test *p* < 0.01; Supplementary Figure 2). The full design-by-treatment interaction random-effects model revealed inconsistency among the designs of the included studies (*Q* = 17.47 and *p* = 0.03). The node-splitting model revealed potential loop inconsistency (Supplementary Figure 3).

### Network meta-analysis outcome: Dialysis-requiring acute kidney injury and mortality

**FIGURE 2:**
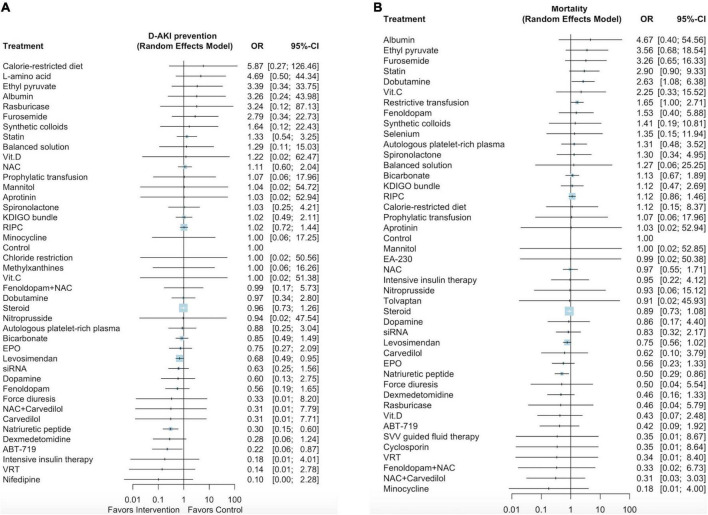
Forest plot of network meta-analysis for dialysis-requiring AKI prevention **(A)**, Forest plot for mortality **(B)**.

Supplementary Figure 4A presents the network plot of the 42 interventions and 31,143 participants from the 119 selected studies that evaluated the effectiveness of the interventions in preventing dialysis-requiring AKI. Three pharmacological interventions (ordered according to the *P*-score: ABT-719 [OR: 0.22, 95% CI: 0.06–0.87], natriuretic peptides [OR: 0.30, 95% CI: 0.15–0.60], and levosimendan [OR: 0.68, 95% CI: 0.49–0.95]), but no non-pharmacological interventions, were associated with the reduced incidence of post–cardiac surgery dialysis-requiring AKI (Figure 2A and Supplementary Document 2). The P scores of the 42 interventions are listed in Supplementary Table 5. We noted low heterogeneity among the studies (*I*^2^ = 0.0%) and no significant publication bias (Egger test *p* = 0.17; Supplementary Figure 4B).

Supplementary Figure 5A presents a network plot of the 44 interventions and 42,374 participants from the 126 selected studies that evaluated mortality after cardiac surgery. Natriuretic peptides and dobutamine were associated with lower mortality rates (OR: 0.50, 95% CI: 0.29–0.86; Figure 2B, Supplementary Document 3) and higher mortality rates (OR: 2.63, 95% CI: 1.08–6.38), respectively. The *P*-scores of the 44 interventions are listed in Supplementary Table 6. We noted low heterogeneity among the studies (*I*^2^ = 0.0%) and no significant publication bias (Egger test *p* = 0.68; Supplementary Figure 5B).

### Network meta-analysis outcome: Intensive care unit and hospital length of stay

**FIGURE 3:**
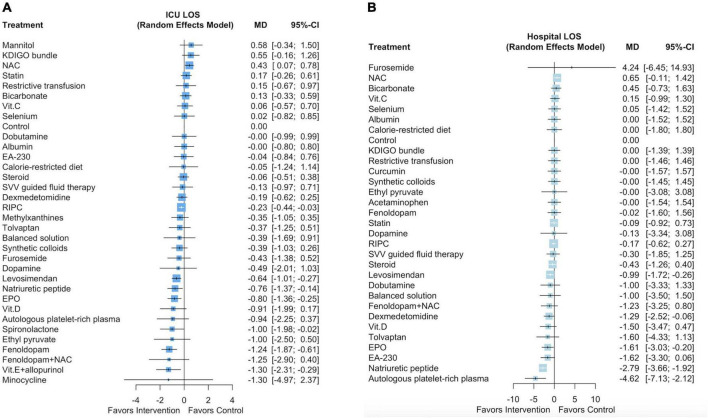
Forest plot of network meta-analysis for ICU **(A)** and hospital **(B)** length of stay.

Supplementary Figure 6A presents a network plot of the 34 interventions and 31,785 participants from the 95 selected studies that evaluated ICU LOS. Six pharmacological interventions (ordered according to the P score: fenoldopam [mean difference (MD): –1.24 days, 95% CI: –1.87 to –0.61], vitamin E with allopurinol [MD: –1.3 days, 95% CI: –2.31 to –0.29], spironolactone [MD: –1.0 days, 95% CI: –1.98 to –0.02], EPO [MD: –0.8 days, 95% CI: –1.36 to –0.25], natriuretic peptides [MD: –0.76 days, 95% CI: –1.37 to –0.14], and levosimendan [MD: –0.64 days, 95% CI: –1.01 to –0.27]) and one non-pharmacological intervention (RIPC, MD: –0.23 days, 95% CI: –0.44 to –0.03) were associated with a shorter ICU LOS (Figure 3A and Supplementary Document 4). The *P* scores of the 34 interventions are listed in Supplementary Table 7. We noted high heterogeneity among the studies (*I*^2^ = 86.3%) but no significant publication bias (Egger test *p* = 0.50; Supplementary Figure 6B).

Supplementary Figure 7A presents a network plot of the 31 interventions and 31,470 participants from the 90 selected studies that evaluated hospital LOS. Five pharmacological interventions (ordered according to the P score: autologous platelet-rich plasma [MD: –4.62 days, 95% CI: –7.13 to –2.12], natriuretic peptides [MD: –2.79 days, 95% CI: –3.66 to –1.92], EPO [MD: –1.61 days, 95% CI: –3.03 to –0.20], dexmedetomidine [MD: –1.29 days, 95% CI: –2.52 to –0.06], and levosimendan [MD: –0.99 days, 95% CI: –1.72 to –0.26]) were associated with a shorter hospital LOS (Figure 3B and Supplementary Document 5). The *P* scores of the 31 interventions are listed in Supplementary Table 8. We noted substantial heterogeneity among the studies (*I*^2^ = 67.6%) but no significant publication bias (Egger test *p* = 0.99; Supplementary Figure 7B).

### Sensitivity analyses

Some of the included studies used non-international AKI criteria (absolute cutoff creatinine levels ranging from 1.5 to 2.0 mg/dL, creatinine elevation of 25–200% of the baseline level, creatinine elevation of > 0.3–0.5 mg/dL from baseline, a decrease in the glomerular filtration rate of > 25% from baseline) or did not report the definition of AKI. We performed a sensitivity analysis excluding these studies to evaluate the robustness of our findings regarding the primary outcome. Supplementary Figure 8A presents a network plot of the 43 interventions and 23,820 participants from the 89 selected studies that evaluated post–cardiac surgery mortality. Four pharmacological interventions (ordered according to the *P* score: methylxanthines [OR: 0.14, 95% CI: 0.03–0.59], natriuretic peptides [OR: 0.19, 95% CI: 0.08–0.44], tolvaptan [OR: 0.35, 95% CI: 0.13–0.95], and dexmedetomidine [OR: 0.49, 95% CI: 0.31–0.78]) and one non-pharmacological intervention (RIPC, OR: 0.78, 95% CI: 0.62–0.98) were associated with a lower incidence of post–cardiac surgery AKI (Supplementary Figure 8B). We noted substantial heterogeneity among the studies (*I*^2^ = 45.8%) but no significant publication bias (Egger test *p* = 0.63; Supplementary Figure 8C).

We performed an additional sensitivity analysis in which studies with some or high risk of bias were excluded. Supplementary Figure 9A presents a network plot of the 23 interventions and 13,923 participants from the 52 selected studies. Only two pharmacological interventions (ordered according to the *P* score: natriuretic peptides [OR: 0.23, 95% CI: 0.06–0.84] and dexmedetomidine [OR: 0.43, 95% CI: 0.27–0.71]) were associated with a lower incidence of post–cardiac surgery AKI (Supplementary Figure 9B). We noted substantial heterogeneity among the studies (*I*^2^ = 45.9%) but no significant publication bias (Egger test *p* = 0.15; Supplementary Figure 9C).

Our final sensitivity analysis excluded studies with small numbers of participants (*n* < 50). Supplementary Figure 10A presents a network plot of the 43,049 participants and 49 interventions from the 123 selected studies. Eight pharmacological interventions (ordered according to the P score: nitroprusside [OR: 0.29, 95% CI: 0.12–0.68], natriuretic peptides [OR: 0.33, 95% CI: 0.20–0.56], fenoldopam [OR: 0.36, 95% CI: 0.17–0.76], tolvaptan [OR: 0.35, 95% CI: 0.14–0.89], NAC with carvedilol [OR: 0.37, 95% CI: 0.16–0.85], dexmedetomidine [OR: 0.44, 95% CI: 0.28–0.70], levosimendan [OR: 0.61, 95% CI: 0.40–0.95], and EPO [OR: 0.62, 95% CI: 0.41–0.94]) and one non-pharmacological intervention (RIPC, OR: 0.78, 95% CI: 0.65–0.95) were associated with a lower incidence of post–cardiac surgery AKI (Supplementary Figure 10B). We noted substantial heterogeneity among the studies (*I*^2^ = 43.8%) and possible publication bias (Egger test *p* < 0.01; Supplementary Figure 10C).

### Subgroup analysis: Heart surgery versus aortic surgery

**FIGURE 4:**
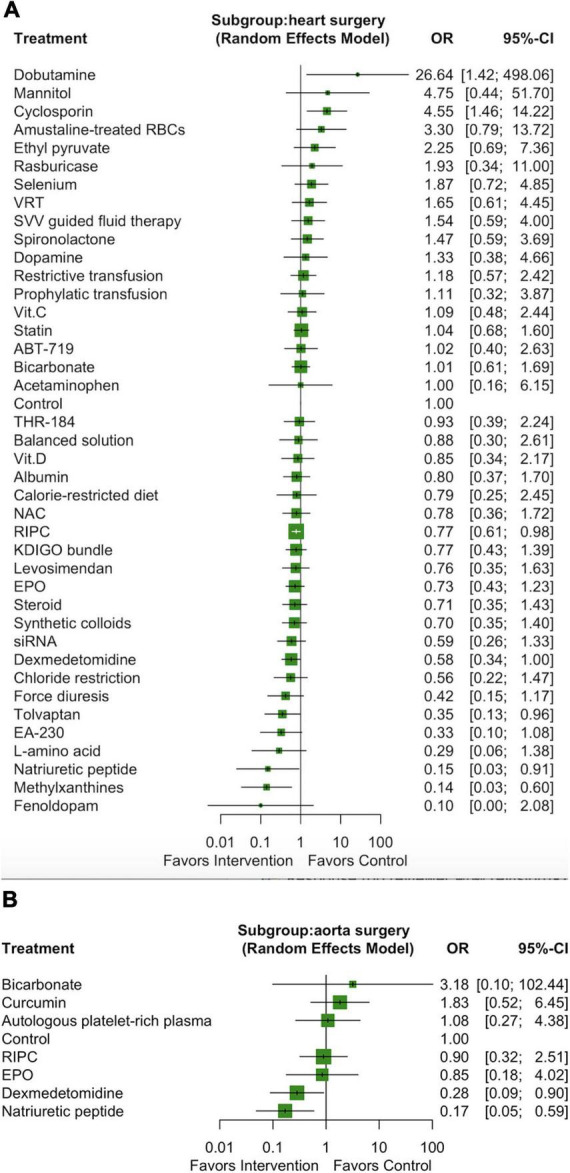
Forest plot of network meta-analysis of subgroup analysis: heart surgery **(A)** and aorta surgery **(B).**

We performed a subgroup analysis including only the 78 studies that enrolled participants who had undergone heart surgery (valve surgery, CABG, or both), which involved a total of 41 interventions and 22,437 participants (Supplementary Figure 11A). Three pharmacological interventions (ordered according to the *P* score: methylxanthines [OR: 0.14, 95% CI: 0.03–0.60], natriuretic peptides [OR: 0.15, 95% CI: 0.03–0.91], and tolvaptan [OR: 0.35, 95% CI: 0.13–0.96]) and one non-pharmacological intervention (RIPC, OR: 0.77, 95% CI: 0.61–0.98) were associated with a lower incidence of AKI among the participants who had undergone heart surgery (Figure 4A). We noted substantial heterogeneity among the studies (*I*^2^ = 50.0%) but no significant publication bias (Egger test *p* = 0.55; Supplementary Figure 11B).

We performed another subgroup analysis including only the 10 studies that enrolled participants who had undergone aortic surgery, which involved a total of 8 interventions and 1354 participants (Supplementary Figure 12A). Only two pharmacological interventions (ordered according to the *P* score: natriuretic peptides [OR: 0.17, 95% CI: 0.05–0.59] and dexmedetomidine [OR: 0.28, 95% CI: 0.09–0.90]) were associated with a lower incidence of AKI among the participants who had undergone aortic surgery (Figure 4B). We noted substantial heterogeneity among the studies (*I*^2^ = 53.5%) but no significant publication bias (Egger test *p* = 0.68; Supplementary Figure 12B).

### Subgroup analysis: Preserved versus impaired baseline renal function

We performed a subgroup analysis including only the 89 studies that enrolled participants with preserved renal function (serum creatinine ≤ 1.2 mg/dL or an estimated glomerular filtration rate of ≥ 6), which involved a total of 41 interventions and 33,787 participants (Supplementary Figure 13A). Five pharmacological interventions (ordered according to the *P* score: natriuretic peptides [OR: 0.30, 95% CI: 0.19–0.49], nitroprusside [OR: 0.29, 95% CI: 0.13–0.67], dexmedetomidine [OR: 0.31, 95% CI: 0.15–0.65], tolvaptan [OR: 0.35, 95% CI: 0.14–0.88], and NAC with carvedilol [OR: 0.37, 95% CI: 0.16–0.84]) and one non-pharmacological intervention (RIPC, OR: 0.76, 95% CI: 0.60–0.96) were associated with a lower incidence of post–cardiac surgery AKI in participants with preserved renal function (Supplementary Figure 13B). We noted substantial heterogeneity among the studies (*I*^2^ = 42.9%) and possible publication bias (Egger test *p* = 0.02; Supplementary Figure 13C).

We performed another subgroup analysis including only the 23 studies that enrolled participants with impaired renal function, which involved a total of 15 interventions and 2669 participants (Supplementary Figure 14A). Only one pharmacological intervention (fenoldopam [OR: 0.26, 95% CI: 0.09–0.70]) was associated with a lower rate of AKI among the participants with impaired renal function (Supplementary Figure 14B). We noted substantial heterogeneity among the studies (*I*^2^ = 40.5%) but no significant publication bias (Egger test *p* = 0.48; Supplementary Figure 14C).

### Assessing risk of bias, ranking of probabilities, and confidence in the network meta-analysis

The results of the risk-of-bias assessment are presented in Supplementary Figure 15 and Supplementary Table 9. Of the included studies, 63.6, 29.6, and 6.8% had low, moderate, and high risk of bias, respectively (Supplementary Document 6).

Information on the potentially effective interventions, their rankings by the *P* score of potentially effective interventions, their effects on primary and secondary outcomes, and confidence in the evidence judged by GRADE framework, is summarized in Table 1.

**TABLE 1:** Summary of potential effective strategies for primary, secondary outcome and rank probability.

AKI		Confidence of evidence	D-AKI		ICU LOS		Hospital LOS		Mortality	
					
Effect	Rank		Effect	Rank	Effect	Rank	Effect	Rank	Effect	Rank
Natriuretic peptide OR: 0.30 (95% CI: 0.19–0.47)	1	Moderate	ABT-719 OR: 0.22 (95% CI: 0.06–0.87)	3	Fenoldopam MD: –1.24 days (95% CI: –1.87 to –0.61)	1	Autologous platelet-rich plasma MD: –4.62 days (95% CI: –7.13 to –2.12)	1	Natriuretic peptide OR: 0.50 (95% CI: 0.29–0.86)	3
Nitroprusside OR: 0.29 (95% CI: 0.12–0.68)	2	Moderate	Natriuretic peptide OR: 0.30 (95% CI: 0.15–0.60)	4	Vitamin E + allopurinol MD: –1.3 days (95% CI: –2.31 to –0.29)	2	Natriuretic peptide MD: –2.79 days (95% CI: –3.66 to –1.92)	2		
Fenoldopam OR: 0.36 (95% CI: 0.17–0.76)	3	Low	Levosimendan OR: 0.68 (95% CI: 0.49–0.95)	13	Spironolactone MD: –1.0 days (95% CI: –1.98 to –0.02)	4	EPO MD: –1.62 days (95% CI: –3.03 to –0.20)	3		
Tolvaptan OR: 0.35 (95% CI: 0.14–0.90)	6	Low			EPO, MD: –0.8 days (95% CI: –1.36 to –0.25)	6	Dexmedetomidine MD: –1.29 days (95% CI: –2.52 to –0.06)	6		
NAC + carvedilol OR: 0.37 (95% CI: 0.16–0.85)	7	Moderate			Natriuretic peptide MD: –0.76 days (95% CI: –1.37 to –0.14)	9	Levosimendan MD: –0.99 days (95% CI: –1.72 to –0.26)	9		
Dexmedetomidine OR: 0.49 (95% CI: 0.32–0.76)	10	Moderate			Levosimendan MD: –0.64 days (95% CI: –1.01 to –0.27)	10				
Levosimendan OR: 0.56 (95% CI: 0.37–0.84)	11	Moderate			RIPC MD: –0.23 days (95% CI: –0.44 to –0.03)	18				
EPO OR: 0.62 (95% CI: 0.41–0.94)	16	Low								
RIPC OR: 0.76 (95% CI: 0.63–0.92)	18	Low								

Confidence of evidence was assessed according to result of NMA regarding AKI prevention by CINeMA. AKI, acute kidney injury; D-AKI, dialysis-requiring acute kidney injury; MD, mean difference; OR, odds ratio.

**FIGURE 5:**
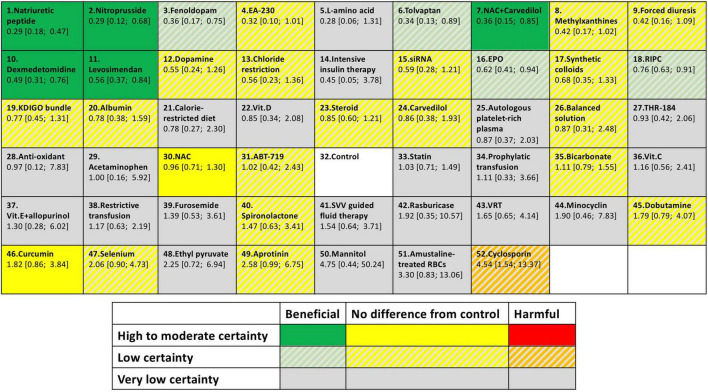
Summary of finding table for AKI prevention. The colors represent in which category of effectiveness and the certainty of evidence of each intervention. Different interventions were ordered according to *P*-score. Each box provides rank probability and relative estimate effect for AKI prevention (in comparison with the control group).

We assessed the degree of confidence in the evidence supporting our findings by using the CINeMA framework. We assessed the confidence of the evidence supporting the effectiveness of each intervention relative to the control group. The results are summarized in Supplementary Table 10. Of the nine strategies determined to be potentially effective in preventing post–cardiac surgery AKI, five (natriuretic peptides, nitroprusside, levosimendan, NAC with carvedilol, and dexmedetomidine) and four (fenoldopam, RIPC, EPO, and tolvaptan) were supported by moderate-confidence and low-confidence evidence, respectively. Detailed descriptions of the bias and confidence assessments are provided in Supplementary Document 7. The trials from ClinicalTrials.gov that were relevant and completed but had no available data for the outcomes of interest are summarized in Supplementary Table 11. We summarized our findings (effect sizes and rankings) regarding the relative effectiveness of the 52 interventions in preventing AKI into findings table (Figure 5), which also includes data regarding confidence in the evidence supporting these interventions (196).

## Discussion

### Discussion

The present study obtained several findings. First, natriuretic peptide therapy was identified to be the most effective strategy for preventing post–cardiac surgery AKI and may be associated with a lower incidence of AKI-related adverse outcomes (dialysis-requiring AKI, extended hospital or ICU LOS, and mortality). Second, RIPC was the most effective non-pharmacological strategy and was associated with a shorter ICU LOS. Third, the results of the sensitivity analysis supported the effectiveness of natriuretic peptides, tolvaptan, dexmedetomidine, and RIPC. After studies with some or high risk of bias were excluded, only natriuretic peptides and dexmedetomidine were identified as effective strategies. Fourth, in the patients who had undergone heart surgery, natriuretic peptides, tolvaptan, and RIPC were effective AKI prevention strategies, whereas in the patients who had undergone aortic surgery, only natriuretic peptides and dexmedetomidine were effective. Fifth, dobutamine and cyclosporine were associated with poor outcomes (higher risks of mortality and AKI, respectively).

In the present study, we demonstrated that natriuretic peptide therapy was the most effective pharmacological intervention for preventing post–cardiac surgery AKI, which is consistent with the results of a previous study. (18) Our study also revealed that natriuretic peptides may be effective for preventing post–cardiac surgery adverse outcomes (dialysis-requiring AKI, hospital and ICU LOSs, and mortality). In addition to their natriuretic effects, natriuretic peptides act as renal vasodilators, antagonists to the renin–angiotensin system, and anti-inflammatory and podocyte-protective agents with potential renoprotective effects (197–199). Eleven of the included studies evaluated the efficacy of natriuretic peptides in preventing post–cardiac surgery AKI (42, 68, 118, 122, 123, 149, 152). However, the doses of natriuretic peptides and natriuretic agents (nesiritide: 0.01 μg/kg/min for 2 or 5 days; human atrial natriuretic peptide: 0.02 μg/kg/min before cardiopulmonary bypass and then 0.01 μg/kg/min for 12 h) varied among these studies. Eleven of the included studies (42, 68, 118, 122, 146–150, 152, 153) compared mortality rates among patients who received natriuretic peptides and controls, and two of these reported that natriuretic peptides are beneficial for survival; (118, 153) however, neither of these studies adopted a AKI definition based on standard criteria, and both had high risk of bias (118, 153). Therefore, these findings should be interpreted with caution. In addition, natriuretic peptides did not reduce the need for dialysis among patients with established AKI (200). Additional trials are required to determine the optimal dose, timing, duration, and cost-effectiveness of natriuretic peptide therapy for preventing post–cardiac surgery AKI.

In the present study, RIPC was associated with a lower incidence of post–cardiac surgery AKI and a shorter ICU LOS. To protect against lethal cardiac and renal ischemia events, remote organs were subjected to brief periods of ischemia, which was achieved using a cuff placed around an upper or lower limb at a pressure of 200–300 mmHg for 2–5 min in 2–5 cycles (4, 201). Iatrogenic intermittent hypoxia activates hypoxia-inducible factor 1, Nrf2 transcription factor, and the anti-inflammatory phenotype, which may exert cardio–renoprotective and neuroprotective effects (201–204). Three of the included studies applied not only preoperative RIPC but also postoperative RIPC (83, 84, 93). Deferrari et al. performed a pairwise meta-analysis of 13 studies published before 2016 and reported that RIPC significantly reduced the incidence of post–cardiac surgery AKI only in a subgroup of patients who received volatile anesthetics (16). In the present study, by analyzing 27 studies that evaluated the protective effects of RIPC, we determined that RIPC was effective but exhibited low potency for preventing post–cardiac surgery AKI. Because RIPC is a non-invasive and low-cost treatment strategy without clinically significant known side effects, it may be incorporated into routine clinical practice.

Two agents (tolvaptan and dexmedetomidine) were determined to protect against post–cardiac surgery AKI in the primary analysis and sensitivity analysis. Dexmedetomidine was also associated with a shorter hospital LOS. Dexmedetomidine, a selective α2-adrenergic receptor agonist, increases renal blood flow and reduces inflammation (4, 205, 206). It also has neuroprotective effects and can prevent postoperative delirium (207, 208). The most commonly used dexmedetomidine prescription protocol in the enrolled studies was 0.4 μg/kg/h after induction with tapering to 0.1 μg/kg/h after extubation for 24 h (55, 151, 161). Only one of the included studies evaluated the effectiveness of tolvaptan. Kishimoto et al. reported that oral tolvaptan started on postoperative day 1 with adequate diuresis could restore fluid balance more rapidly and was associated with a lower risk of AKI (98).

Several other pharmacological interventions, including fenoldopam, levosimendan, and EPO, might be associated with a lower risk of post–cardiac surgery AKI according to our NMA and a previous study (18). Fenoldopam is a renal vasodilator and can increase renal perfusion (12), and levosimendan is an inotrope and vasodilator that can increase renal perfusion (12, 209). However, seven of the eight studies on levosimendan and three of four studies on fenoldopam did not use international AKI criteria. Within-study bias was also a concern. In the current study, fenoldopam, levosimendan, and EPO were not associated with a lower risk of AKI in the sensitivity analysis. Methylxanthines were identified as a potentially effective intervention in the sensitivity analysis and subgroup analysis. However, only three studies compared methylxanthines with a control. Two of these three studies did not use international AKI criteria, and in the remaining study, the incidence of AKI in the control group was significantly higher than that in the intervention group (23.6% vs. 4.2%) (155). According to RoB 2, this study had some risk of bias. Therefore, our findings regarding the efficacy of methylxanthines in the subgroup analysis and sensitivity analysis should be interpreted carefully.

Other non-pharmacological interventions, including the KDIGO AKI prevention bundle, chloride-restrictive fluids, and intensive sugar control, were ineffective in preventing post–cardiac surgery AKI according to our NMA. Notably, the ADQI guidelines suggest the avoidance of glucose variability but not intensive sugar control (6). Cohort studies demonstrated that compared with intensive sugar control, moderate sugar control is associated with lower mortality rates (210), and that high sugar levels (intraoperative glucose concentrations > 150 mg/dL) might be associated with a higher risk of AKI (211). The GLUCO-CABG trial compared intensive sugar control (100–140 mg/dL) with regular sugar control (141–180 mg/dL) and revealed no significant difference in perioperative complications between the two strategies (212). Two of the studies included in our analysis defined the intensive sugar control level as 80–110 and 100–120 mg/dL, respectively (121, 178). Because the results of our NMA revealed that intensive sugar control is ineffective in preventing post–cardiac surgery AKI and is potentially associated with a higher risk of hypoglycemia and mortality, we argue that intensive sugar control should be applied with caution. Other non-pharmacological interventions, namely the KDIGO AKI prevention bundle and chloride-restrictive fluids, are low-cost and are not associated with any clinically significant side effects. Therefore, although they can be safely applied in clinical settings, additional studies are required to further evaluate their efficacy.

### Strengths and limitations

Our study has several strengths. First, we assessed the effectiveness of both pharmacological and non-pharmacological interventions in preventing post–cardiac surgery AKI. Second, we conducted a sensitivity analysis to examine the robustness of our findings because many early studies did not use international AKI criteria or did not report the definition of AKI employed and because some of the studies had a risk of bias. Third, we examined other important outcomes related to post–cardiac surgery AKI, namely dialysis-requiring AKI, mortality, and ICU and hospital LOS. Fourth, we assessed the confidence in the results of our NMA by using the CINeMA framework.

Our study also has several limitations. First, we did not analyze the effectiveness of interventions with different dosages or protocols separately. Second, we did not analyze the effectiveness of surgical procedures and CPB-related techniques (on-pump and off-pump CABG, pulsatile CPB, different anticoagulation agents for CPB, and cerebral protection during CPB). Third, we did not analyze the potential renoprotective effect of volatile anesthetics and we did not compare the effectiveness of vasopressin and norepinephrine in preventing postoperative vasoplegia. Bonanni et al. reported that compared with propofol, volatile anesthetics are associated with lower mortality rates among patients who had undergone cardiac surgery (213). The effect of different anesthetics warrant further examination. Fourth, most of the included studies were two-arm studies that compared the effectiveness of a single intervention with a control, and most of the comparisons were drawn on the basis of indirect evidence. Moreover, confidence in the evidence supporting most of the potentially effective strategies was moderate to low (according to the CiNeMA framework).

## Conclusion

According to the results of our NMA, we identified nine interventions that are potentially effective in preventing post–cardiac surgery AKI. Among them, natriuretic peptides were the most effective and were determined to be associated with a lower incidence of AKI-related adverse outcomes. RIPC was the only effective non-pharmacological intervention and was associated with a shorter ICU LOS. All nine strategies were supported by only moderate- to low-confidence evidence, and among them, only natriuretic peptides and dexmedetomidine were identified as effective in the sensitivity analysis. Our subgroup analysis revealed heterogeneity in the outcomes between patients who underwent heart (CABG, heart valve surgery) and aortic surgery. The effectiveness of the interventions examined in this study, as well as the optimal dosages and protocols for and the cost-effectiveness of such interventions, must be further explored in additional RCTs in the future.

## Data availability statement

The original contributions presented in this study are included in the article/Supplementary material, further inquiries can be directed to the corresponding author.

## Author contributions

J-JC and TL: methodology and writing and writing—original draft preparation. J-JC, GK, and Y-TH: formal analysis. J-JC, TL, and GK: data extraction. C-CL, J-JC, and TL: table and figures formation. P-RC, S-WC, H-YY, H-HH, C-HY, and C-CH: writing—review and editing. Y-CC and C-HC: project administration. All authors have read and approved the final manuscript.
