# Supplementary material for: Strategies for post–cardiac surgery acute kidney injury prevention: A network meta-analysis of randomized controlled trials
Source: Front Cardiovasc Med. 2022 Sep 27;9:960581. doi: 10.3389/fcvm.2022.960581 (PMC9555275; doi:10.3389/fcvm.2022.960581)

Supplementary Figure 1. PRISMA Flow Diagram

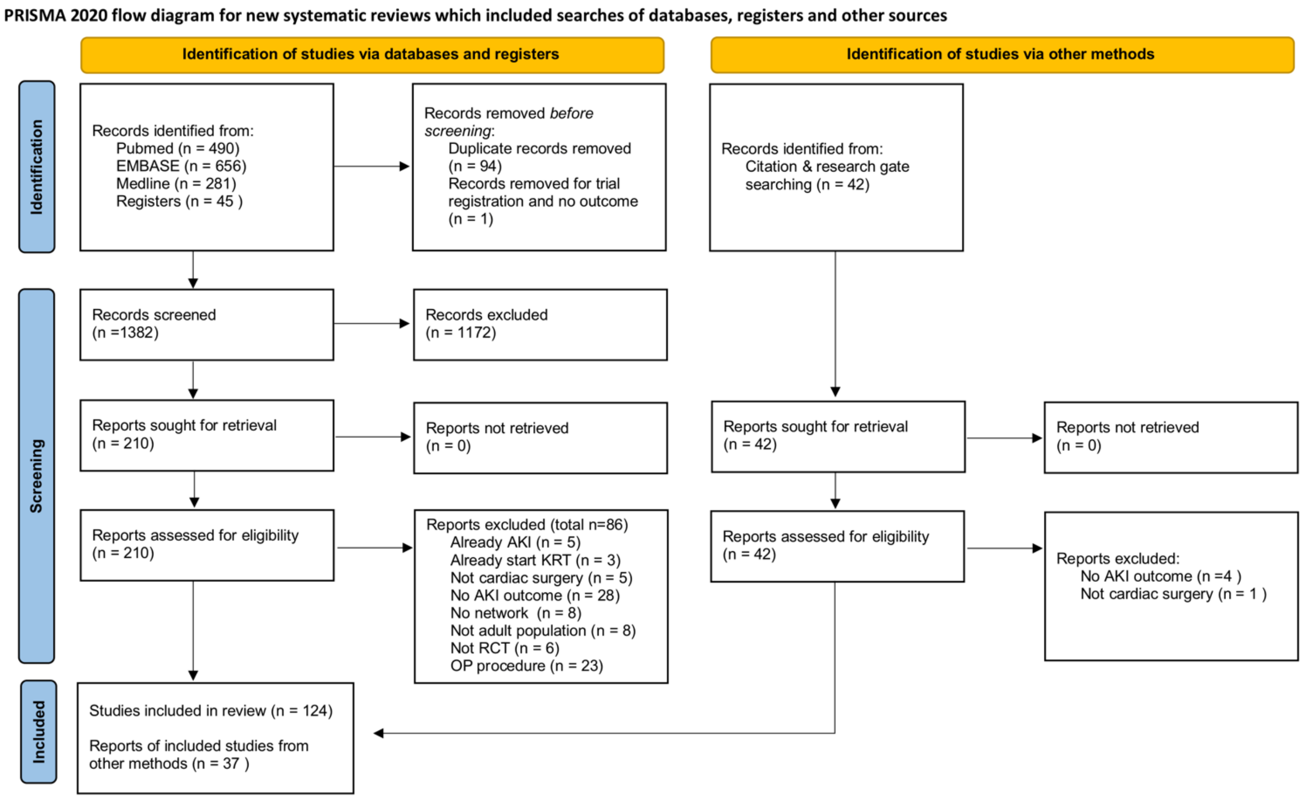

From: Page MJ, McKenzie JE, Bossuyt PM, Boutron I, Hoffmann TC, Mulrow CD, et al. The PRISMA 2020 statement: an updated guideline for reporting systematic reviews. BMJ 2021;372:n71. doi: 10.1136/bmj.n71. For more information, visit: http://www.prisma-statement.org/

Supplementary Figure 2. Comparison-adjusted funnel plots for AKI prevention

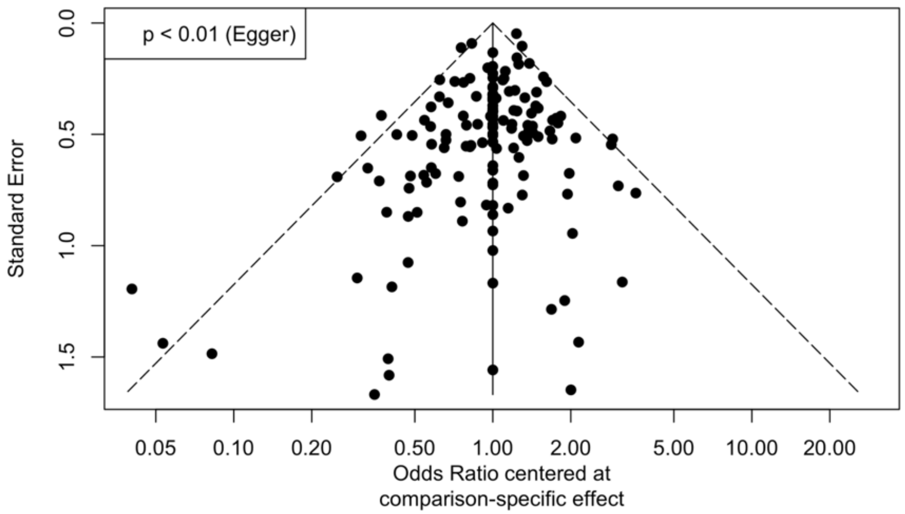

Supplementary Figure 3. Node-splitting plots for inconsistency tests regarding AKI prevention

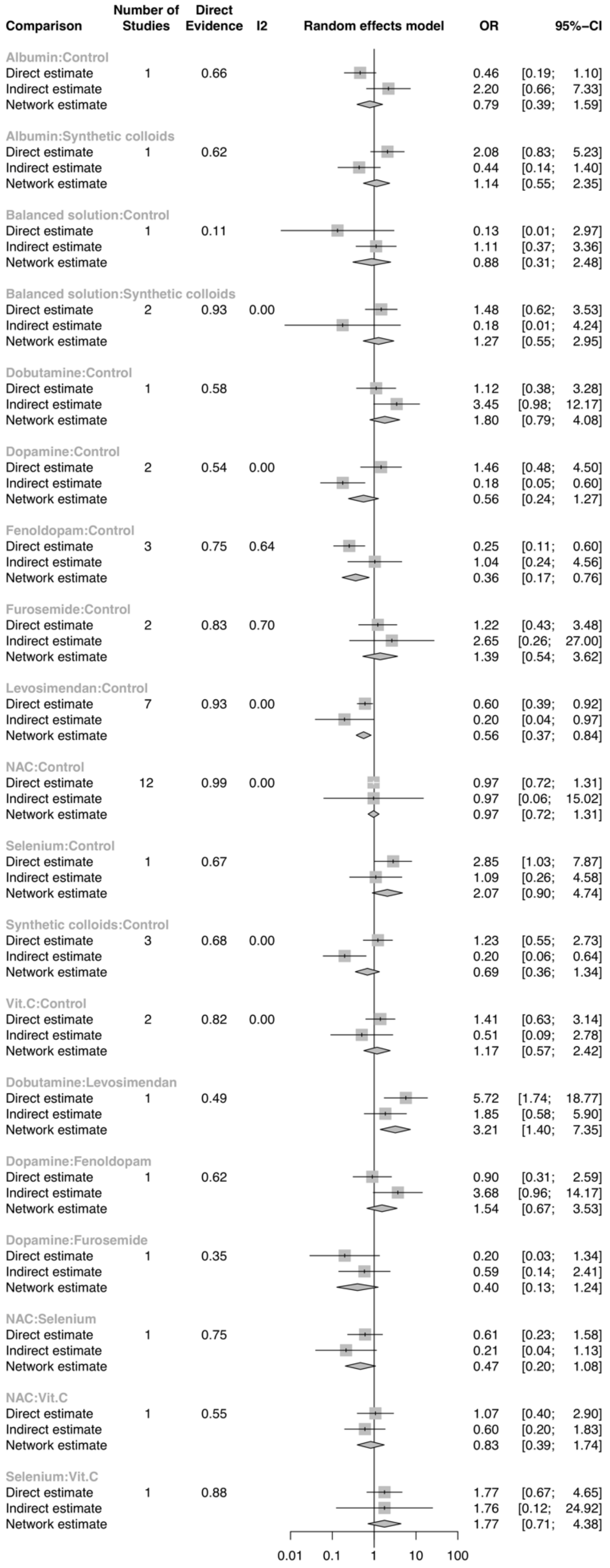

Supplementary Figure 4. Network plot of eligible comparisons among interventions for dialysis-requiring AKI prevention

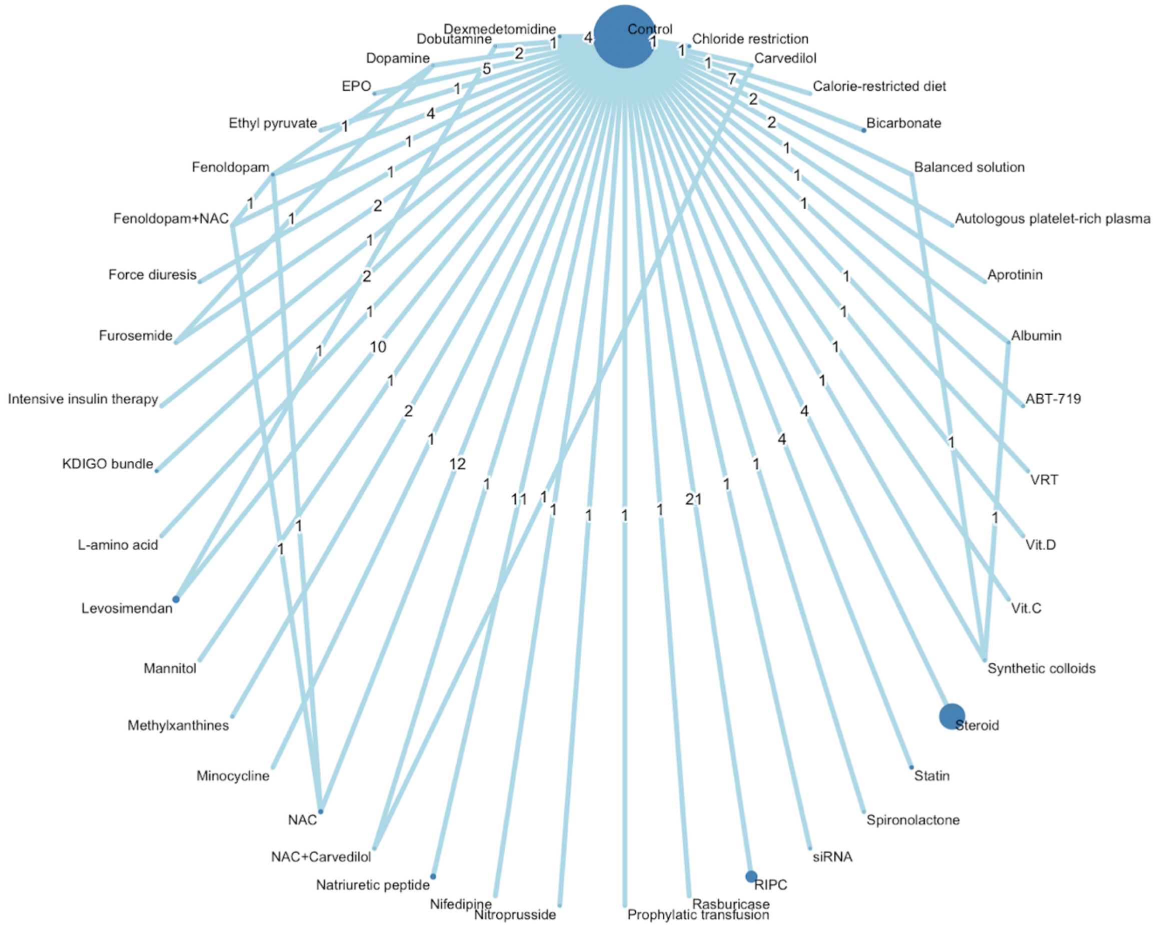

(A)

Abbreviation: EPO: Erythropoietin, NAC: N-acetyl cysteine, RIPC: Remote ischemic preconditioning, VRT: Volume replacement therapy, Vit.C: Vitamin C, Vit.D: Vitamin D

Supplementary Figure 4. Comparison-adjusted funnel plots for dialysis-requiring AKI prevention (B)

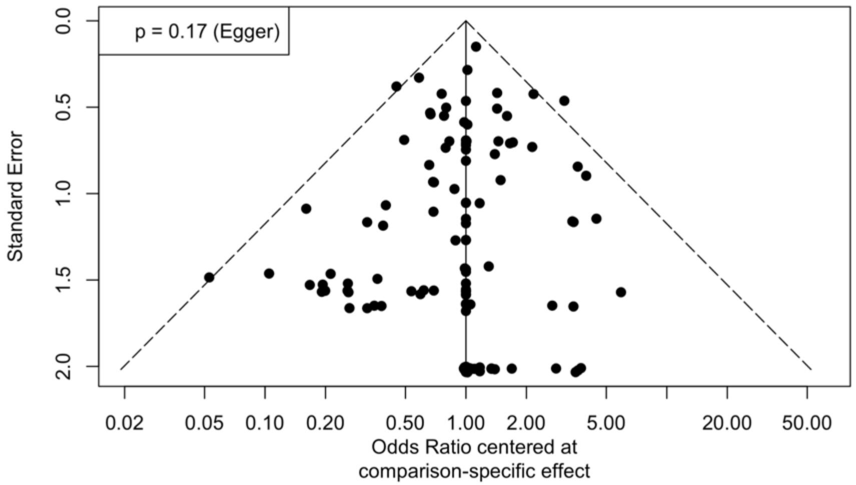

Supplementary Figure 5. Network plot of eligible comparisons among interventions for mortality (A)

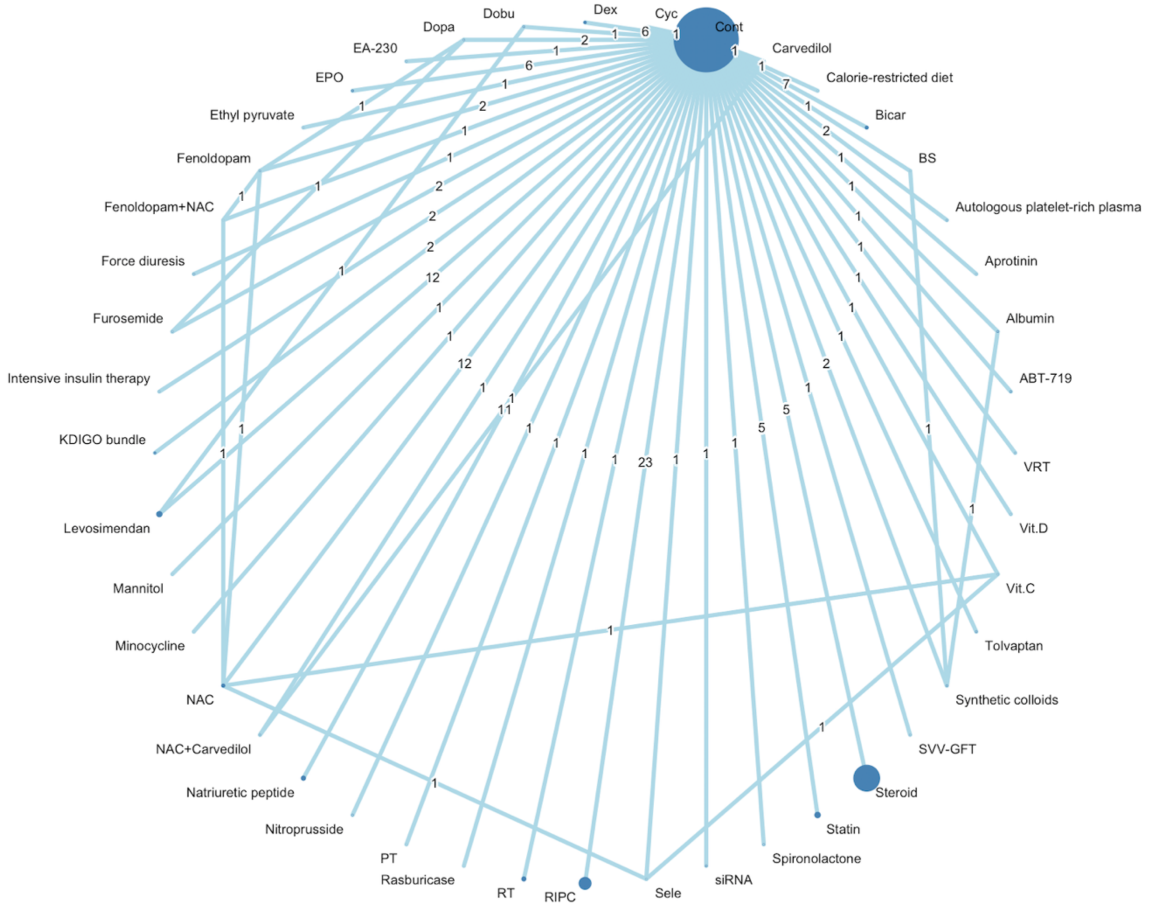

Abbreviation: BS: Balanced solution, Bicar: Bicarbonate, Cont: Control, Cyc: Cyclosporin, Dex: Dexmedetomidine, Dobu:Dobutamine, Dopa: Dopamine, EPO: Erythropoietin, NAC: N-acetyl cysteine, PT: Prophylactic transfusion, RT: Restrictive transfusion, RIPC: Remote ischemic preconditioning, Sele: Selenium, SVV-GFT: Stroke volume variation guided fluid therapy, Vit.C: Vitamin C, Vit.D: Vitamin D, VRT: Volume replacement therapy

Supplementary Figure 5. Comparison-adjusted funnel plots for mortality (B)

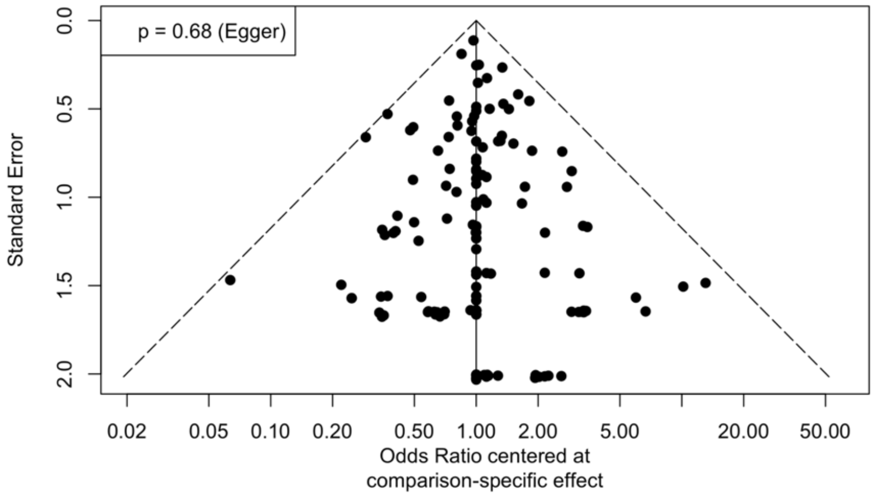

Supplementary Figure 6. Network plot of eligible comparisons among interventions for ICU length of stay (A)

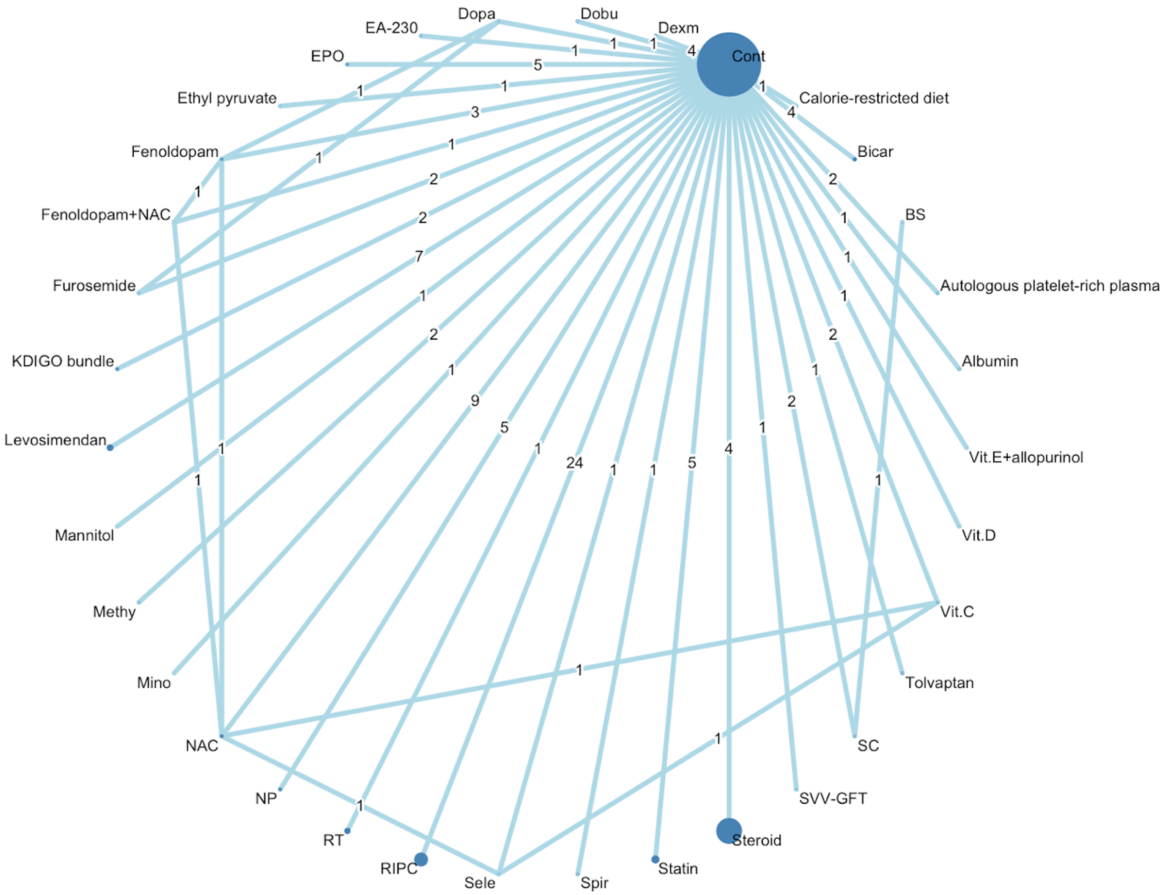

Abbreviation: BS: Balanced solution, Bicar: Bicarbonate, Cont: Control, Dexm: Dexmedetomidine, Dobu:Dobutamine, Dopa: Dopamine, EPO: Erythropoietin, NAC: N-acetyl cysteine, Methy: Methylxanthines, Mino: Minocycline, NAC: N-acetyl cysteine, NP: Natriuretic peptide, RT: Restrictive transfusion, RIPC: Remote ischemic preconditioning, Sele: Selenium, Spiro: Spironolactone, SVV-GFT: Stroke volume variation guided fluid therapy, SC: Synthetic colloids, Vit.C: Vitamin C, Vit.D: Vitamin D , Vit.E: Vitamin E, VRT: Volume replacement therapy

Supplementary Figure 6. Comparison-adjusted funnel plots for ICU length of stay (B)

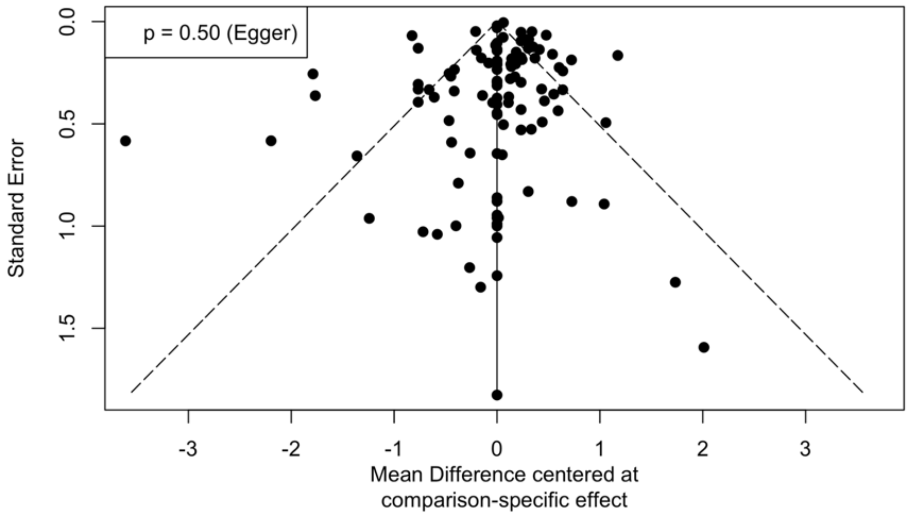

Supplementary Figure 7. Network plot of eligible comparisons among interventions for hospital of stay (A)

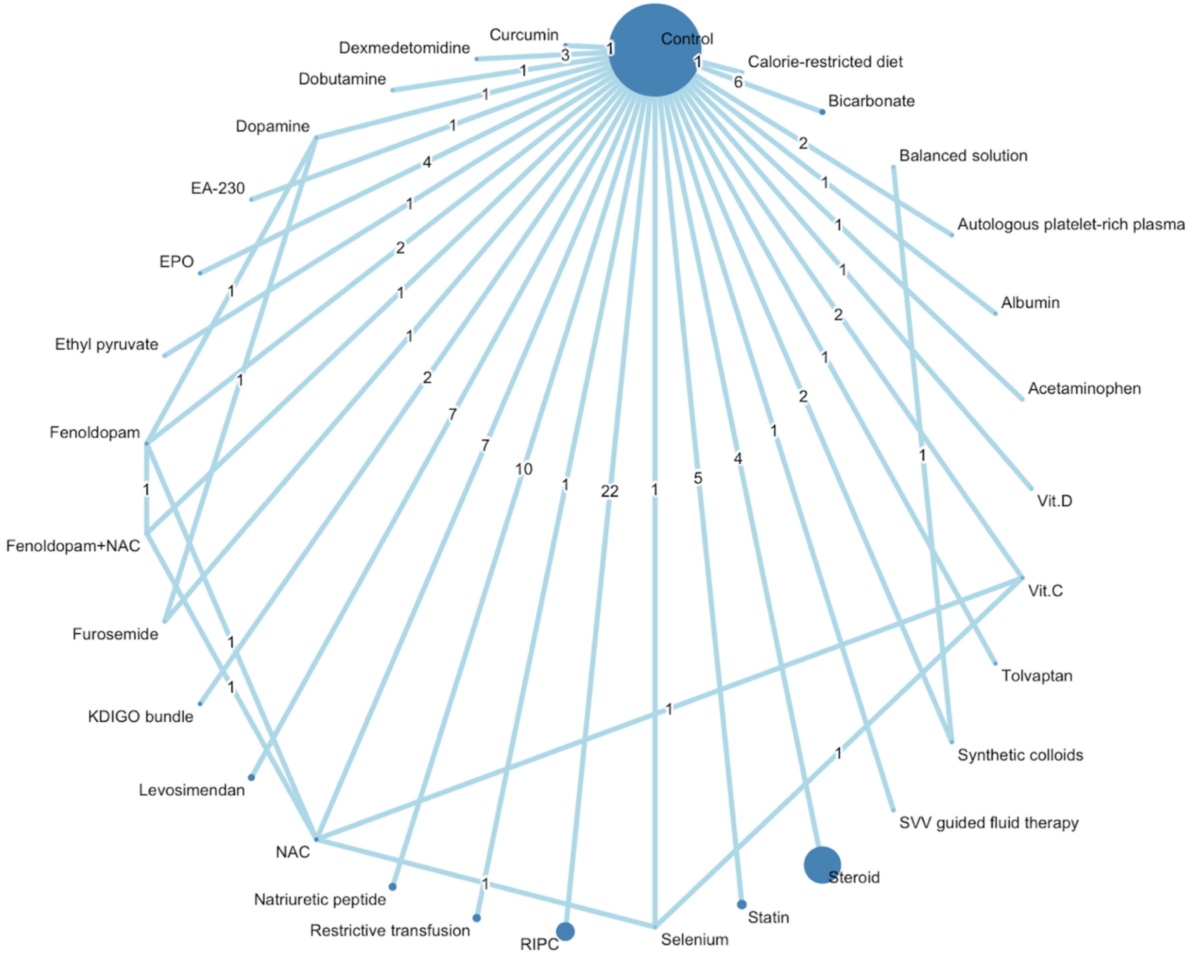

Abbreviation: EPO: Erythropoietin, NAC: N-acetyl cysteine, RIPC: Remote ischemic preconditioning, Vit.C: Vitamin C, Vit.D: Vitamin D, VRT: Volume replacement therapy

Supplementary Figure 7. Comparison-adjusted funnel plots for hospital length of stay (B)

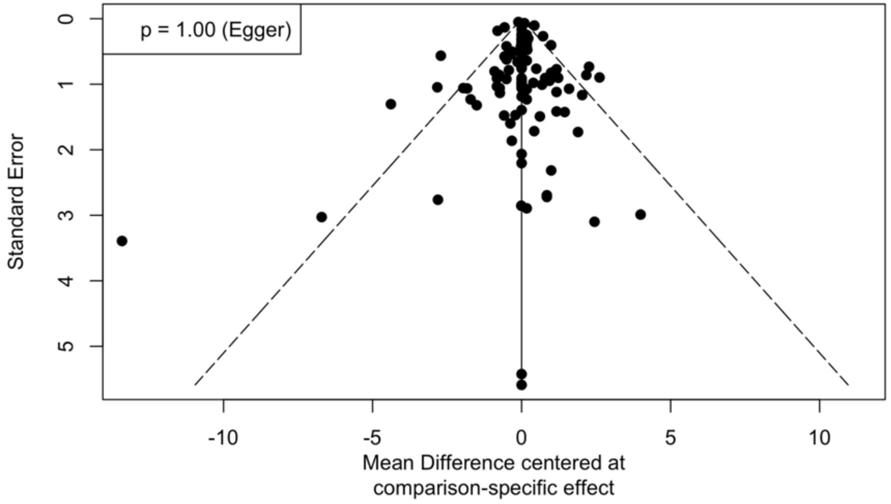

Supplementary Figure 8. Network plot of eligible comparisons among interventions for AKI in sensitivity analysis excluding studies without standard AKI criteria (A)

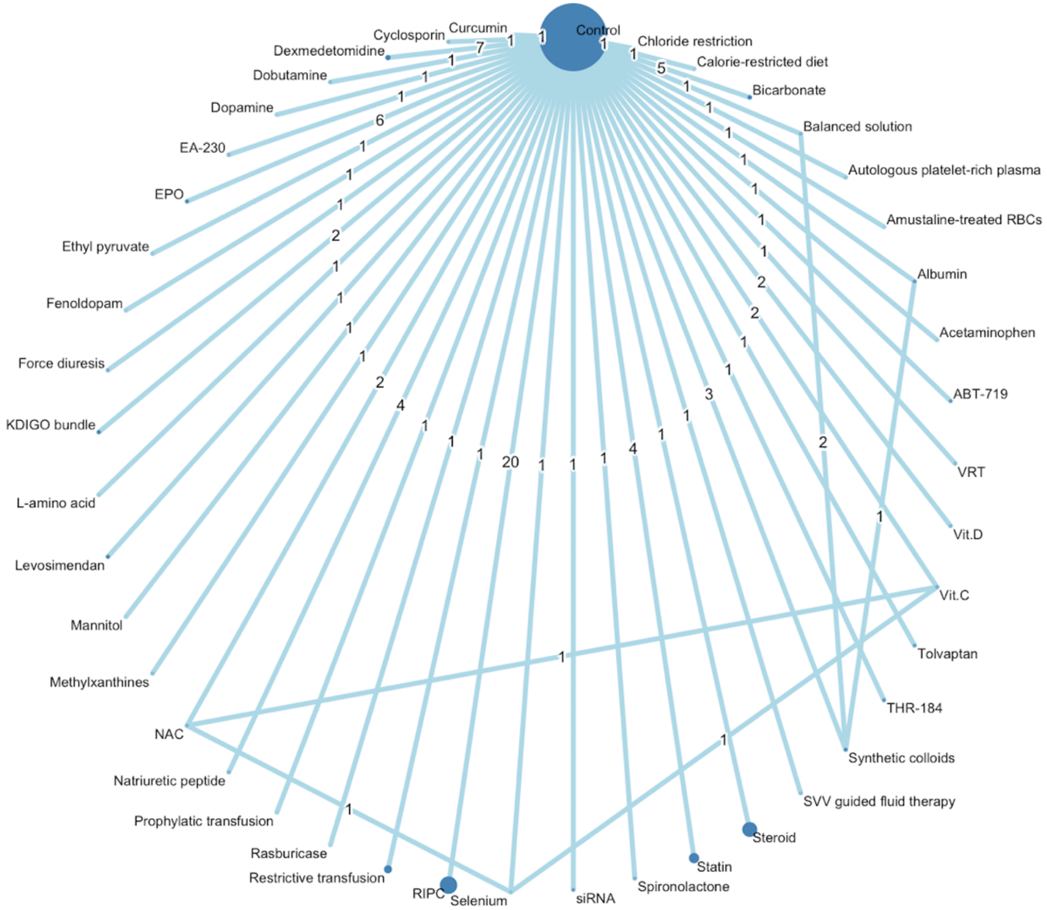

Abbreviation: EPO: Erythropoietin, NAC: N-acetyl cysteine, RIPC: Remote ischemic preconditioning, Vit.C: Vitamin C, Vit.D: Vitamin D, VRT: Volume replacement therapy

Supplementary Figure 8. Forest plot of sensitivity analysis excluding studies without standard AKI criteria (B)

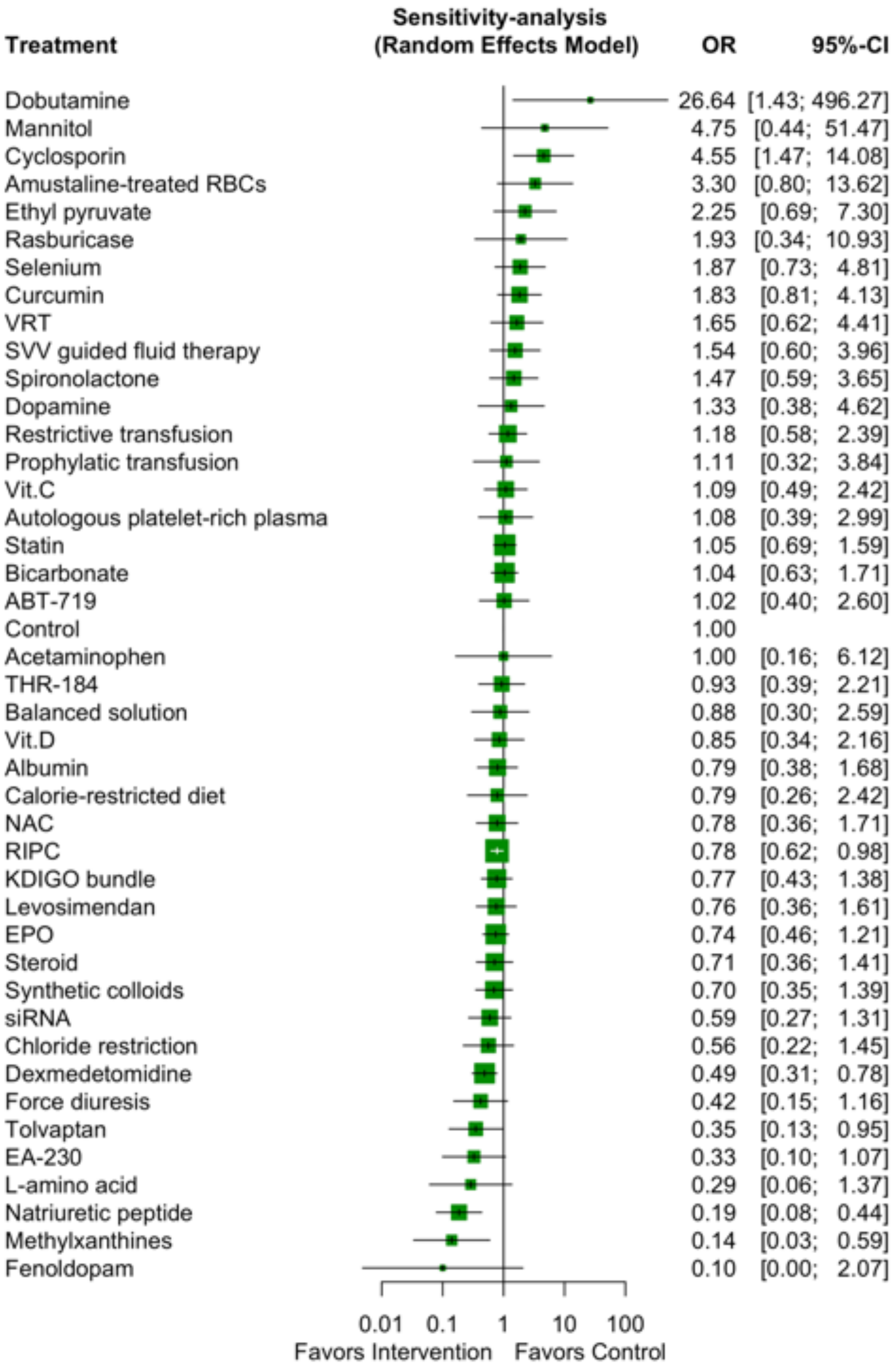

Supplementary Figure 8. Comparison-adjusted funnel plots for sensitivity analysis excluding studies without standard AKI criteria (C)

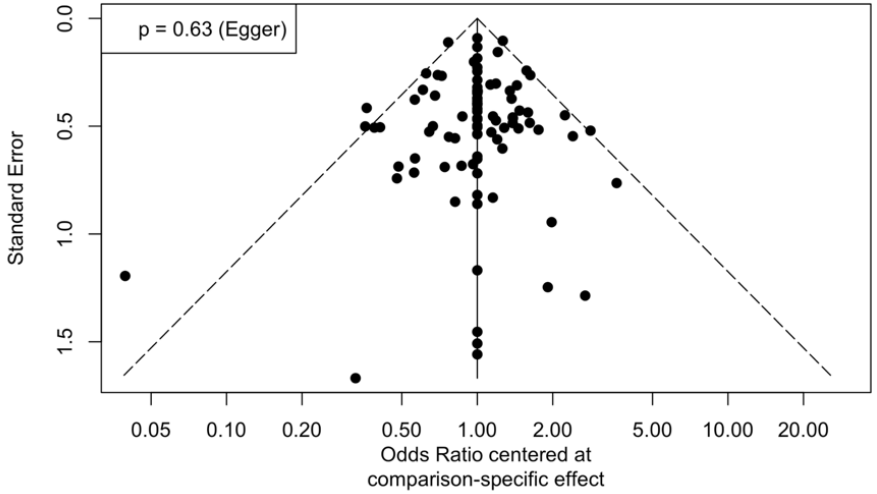

Supplementary Figure 9. Network plot of eligible comparisons among interventions for AKI in sensitivity analysis excluding studies without standard AKI criteria and risk of bias (A)

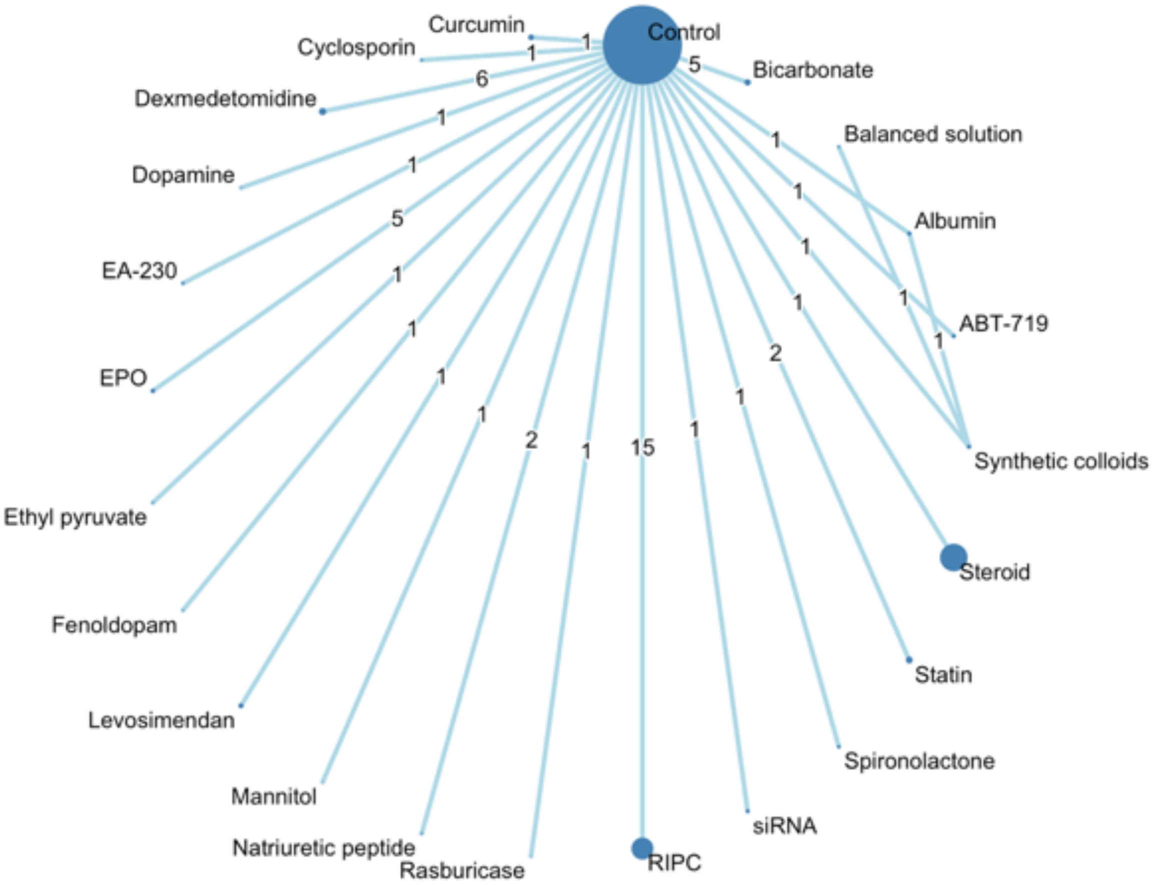

Abbreviation: EPO: Erythropoietin, NAC: N-acetyl cysteine, RIPC: Remote ischemic preconditioning, Vit.C: Vitamin C, Vit.D: Vitamin D, VRT: Volume replacement therapy

Supplementary Figure 9. Forest plot of sensitivity analysis excluding studies without standard AKI criteria and risk of bias (B)

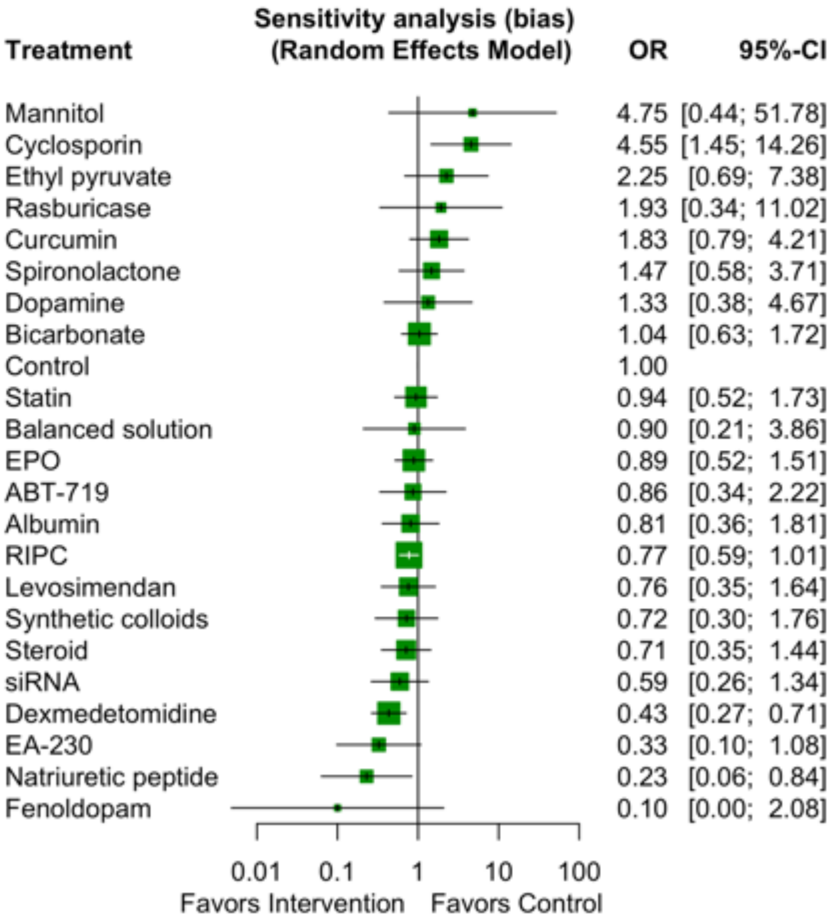

Supplementary Figure 9. Comparison-adjusted funnel plots for sensitivity analysis excluding studies without standard AKI criteria and risk of bias (C)

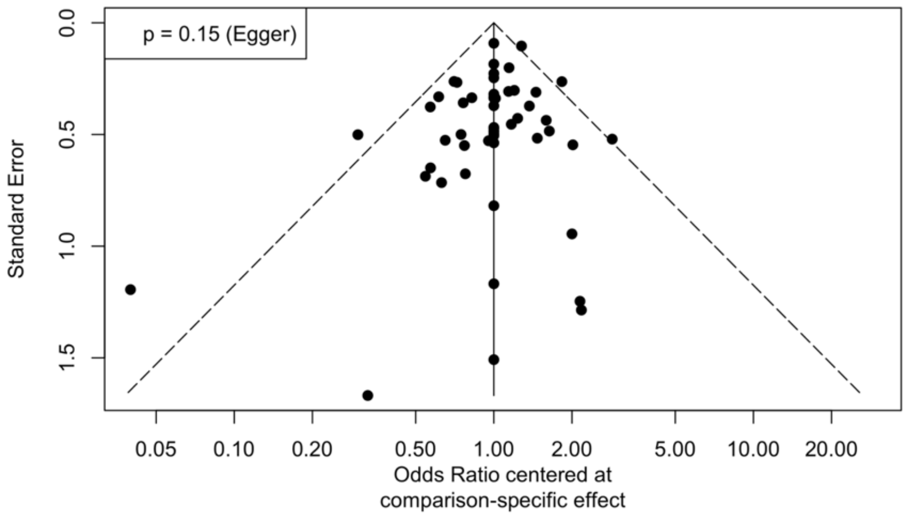

Supplementary Figure 10. Network plot of eligible comparisons among interventions for AKI in sensitivity analysis excluding studies with small number of participants (A)

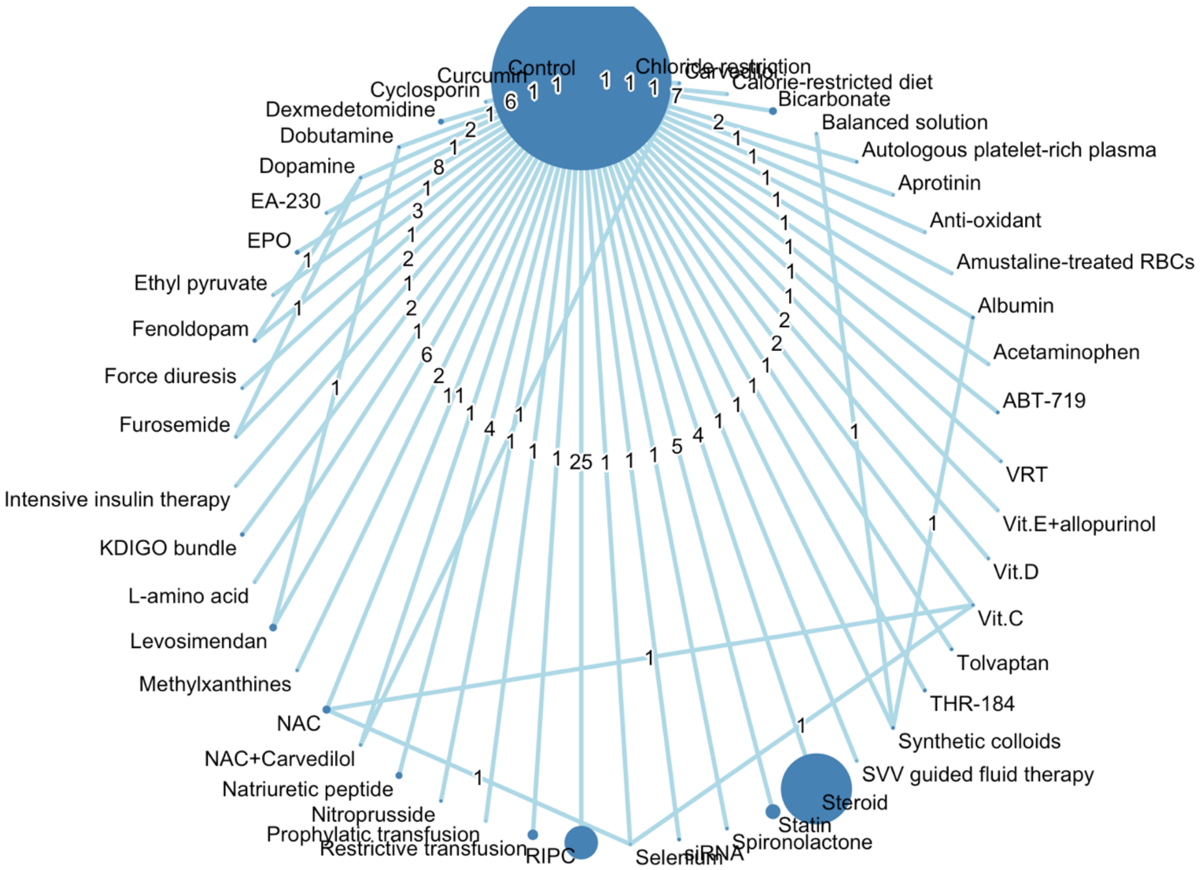

Supplementary Figure 10. Forest plot of sensitivity analysis excluding studies with small number of participants (B)

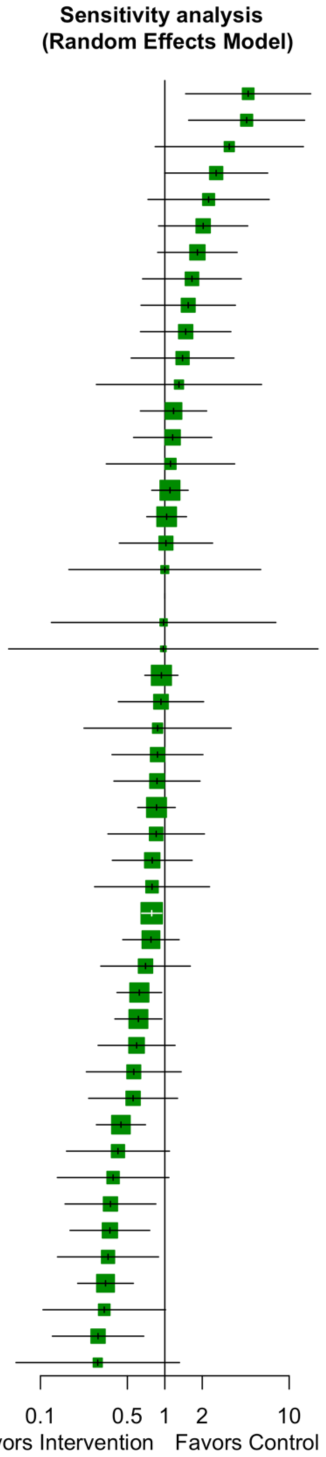

Treatment	OR	95%-CI
Dobutamine	4.68	[1.47; 14.90]
Cyclosporin	4.55	[1.56; 13.31]
Amustaline-treated RBCs	3.30	[0.84; 13.02]
Aprotinin	2.59	[1.00; 6.72]
Ethyl pyruvate	2.25	[0.73; 6.91]
Selenium	2.03	[0.89; 4.65]
Curcumin	1.83	[0.88; 3.82]
VRT	1.65	[0.66; 4.12]
SVV guided fluid therapy	1.54	[0.64; 3.69]
Spironolactone	1.47	[0.64; 3.40]
Furosemide	1.39	[0.54; 3.60]
Vit.E+allopurinol	1.30	[0.28; 6.00]
Restrictive transfusion	1.18	[0.64; 2.17]
Vit.C	1.16	[0.56; 2.38]
Prophylatic transfusion	1.11	[0.34; 3.65]
Bicarbonate	1.10	[0.79; 1.54]
Statin	1.03	[0.72; 1.49]
ABT-719	1.02	[0.43; 2.42]
Acetaminophen	1.00	[0.17; 5.91]
Control	1.00	
Anti-oxidant	0.98	[0.12; 7.82]
Intensive insulin therapy	0.97	[0.06; 17.06]
NAC	0.94	[0.69; 1.27]
THR-184	0.93	[0.42; 2.05]
Balanced solution	0.87	[0.22; 3.42]
Autologous platelet-rich plasma	0.87	[0.38; 2.03]
Carvedilol	0.86	[0.39; 1.92]
Steroid	0.86	[0.61; 1.21]
Vit.D	0.85	[0.35; 2.08]
Albumin	0.79	[0.38; 1.66]
Calorie-restricted diet	0.79	[0.27; 2.29]
RIPC	0.78	[0.65; 0.95]
KDIGO bundle	0.77	[0.46; 1.30]
Synthetic colloids	0.70	[0.31; 1.60]
EPO	0.62	[0.41; 0.94]
Levosimendan	0.61	[0.40; 0.95]
siRNA	0.59	[0.29; 1.21]
Chloride restriction	0.56	[0.23; 1.35]
Dopamine	0.56	[0.24; 1.27]
Dexmedetomidine	0.44	[0.28; 0.70]
Force diuresis	0.42	[0.16; 1.09]
Methylxanthines	0.38	[0.14; 1.08]
NAC+Carvedilol	0.37	[0.16; 0.85]
Fenoldopam	0.36	[0.17; 0.76]
Tolvaptan	0.35	[0.14; 0.89]
Natriuretic peptide	0.33	[0.20; 0.56]
EA-230	0.33	[0.10; 1.01]
Nitroprusside	0.29	[0.12; 0.68]
L-amino acid	0.29	[0.06; 1.31]

Supplementary Figure 10. Comparison-adjusted funnel plots for sensitivity analysis excluding studies with small number of participants (C)

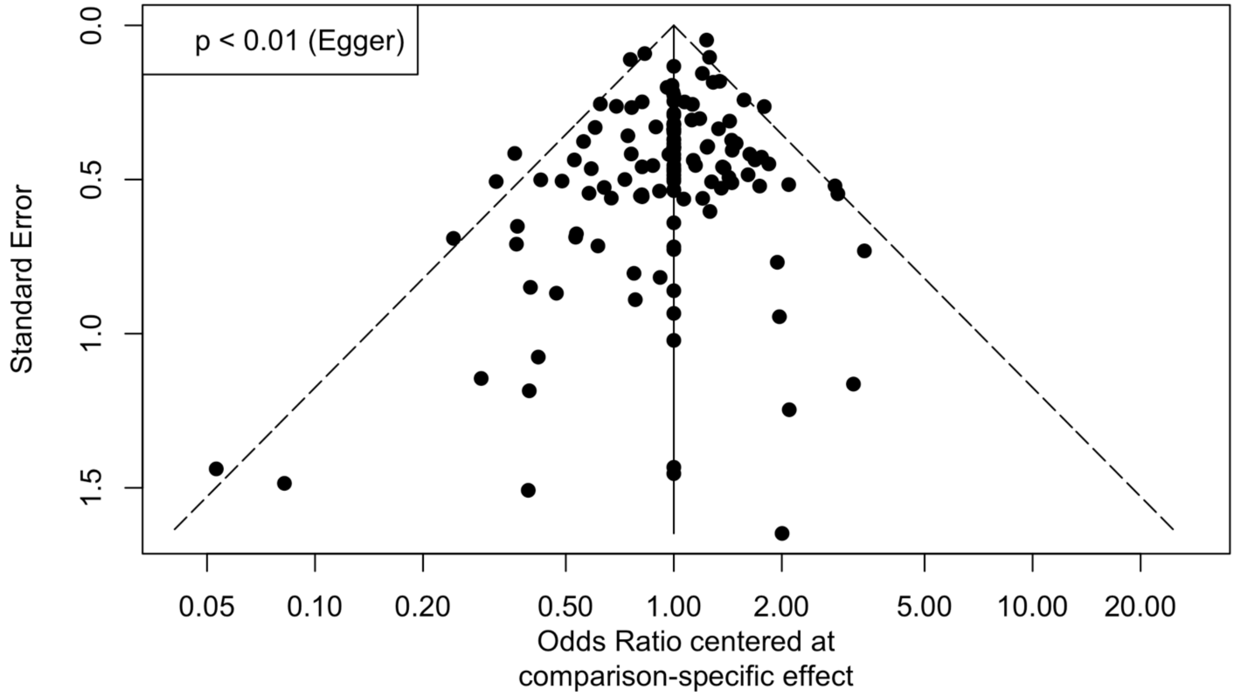

Supplementary Figure 11. Network plot of eligible comparisons among interventions for AKI prevention in subgroup analysis: heart surgery (A)

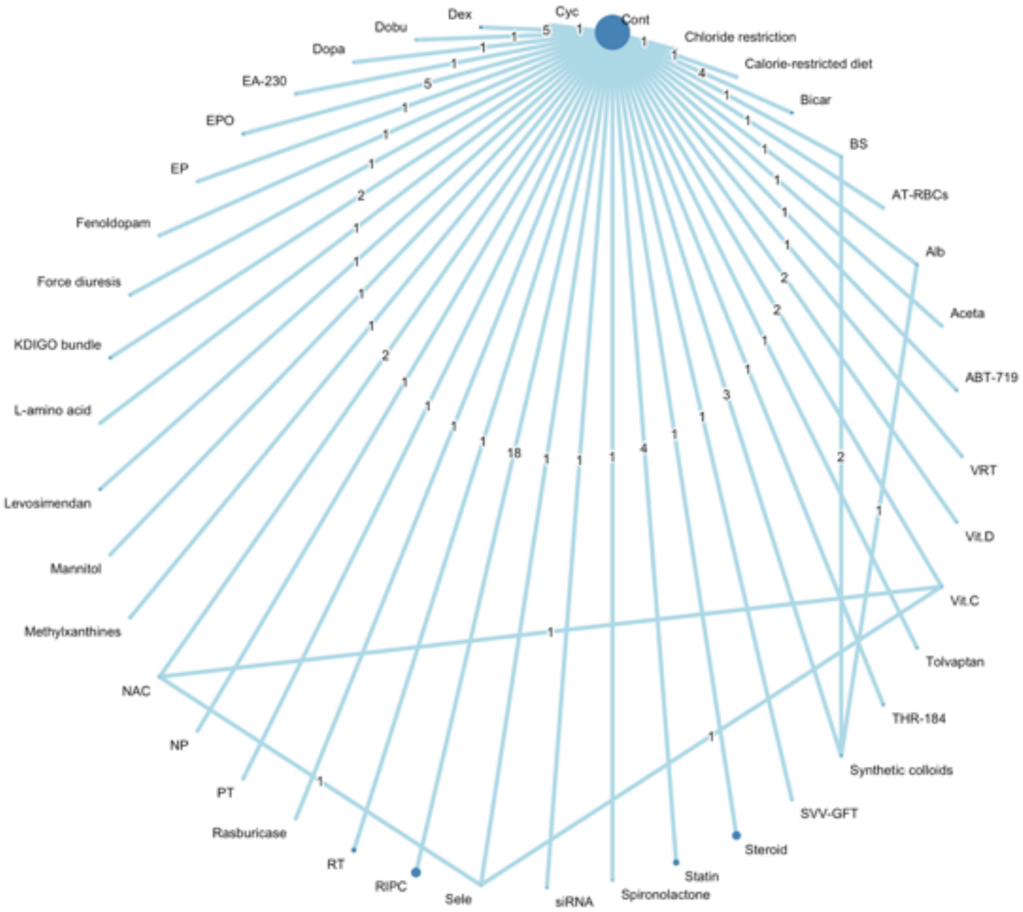

Abbreviation: EPO: Erythropoietin, NAC: N-acetyl cysteine, RIPC: Remote ischemic preconditioning, Vit.C: Vitamin C, Vit.D: Vitamin D, VRT: Volume replacement therapy

Supplementary Figure 11. Comparison-adjusted funnel plots for AKI prevention in subgroup analysis: heart surgery (B)

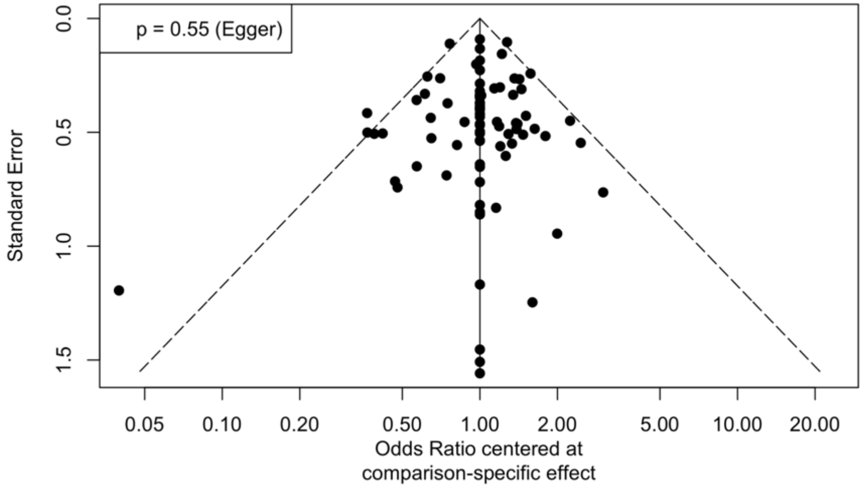

Supplementary Figure 12. Network plot of eligible comparisons among interventions for AKI prevention in subgroup analysis: aorta surgery (A)

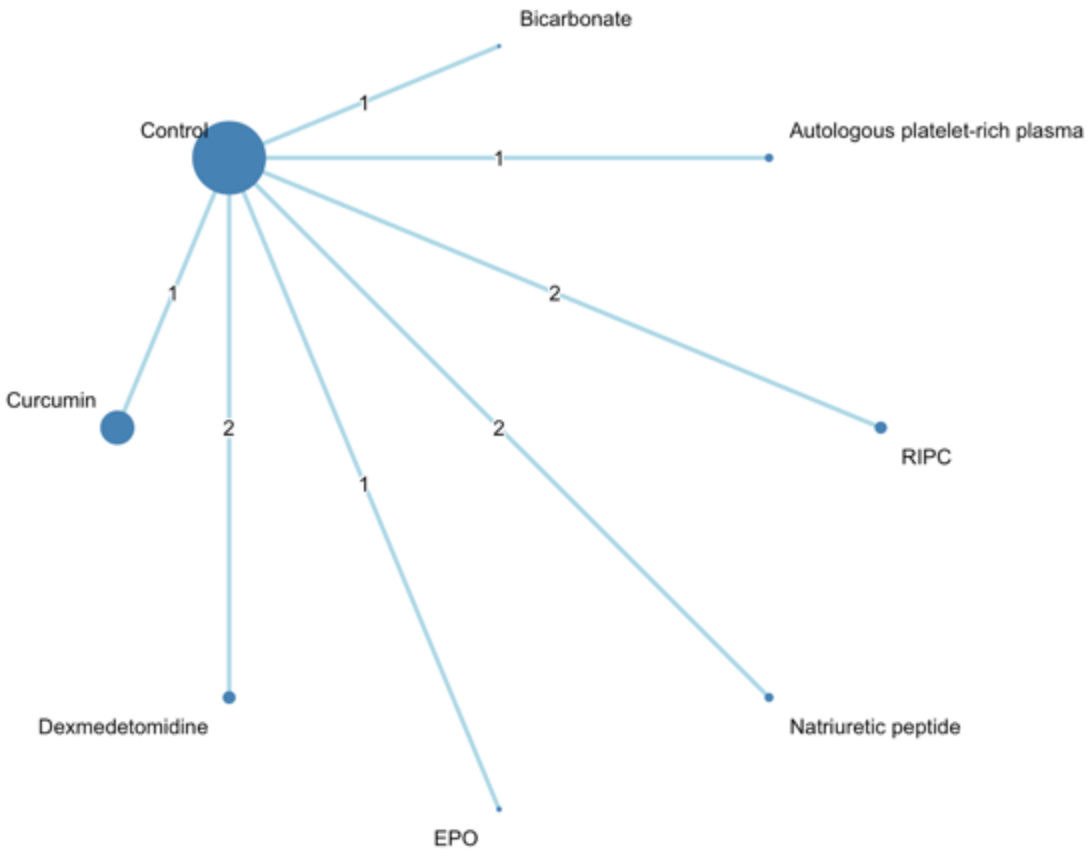

Abbreviation: EPO: Erythropoietin,, RIPC: Remote ischemic preconditioning

Supplementary Figure 12. Comparison-adjusted funnel plots for AKI prevention in subgroup analysis: aorta surgery (B)

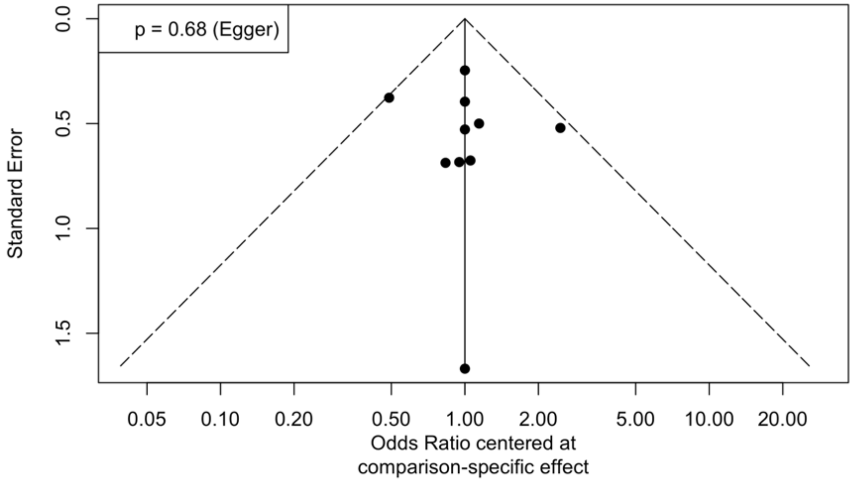

Supplementary Figure 13. Network plot of eligible comparisons among interventions for AKI prevention in subgroup analysis: preserved renal function (A)

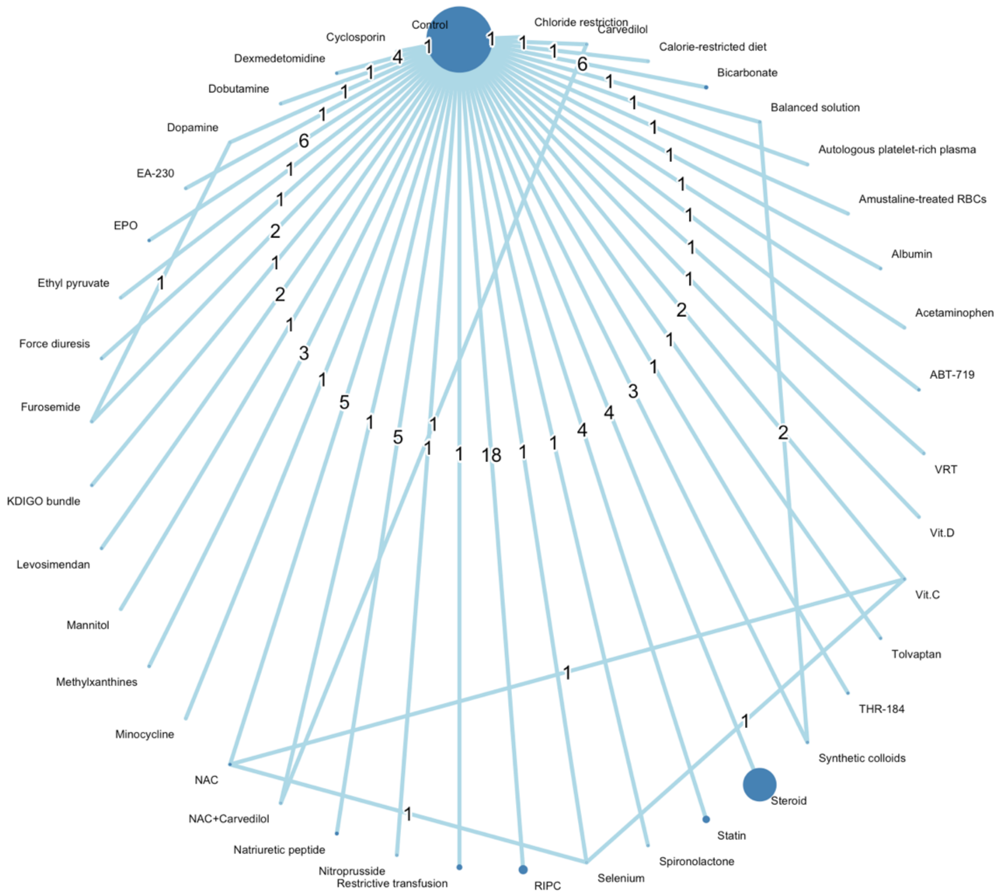

Supplementary Figure 13. Forest plot of network meta-analysis of subgroup analysis: preserved renal function (B)

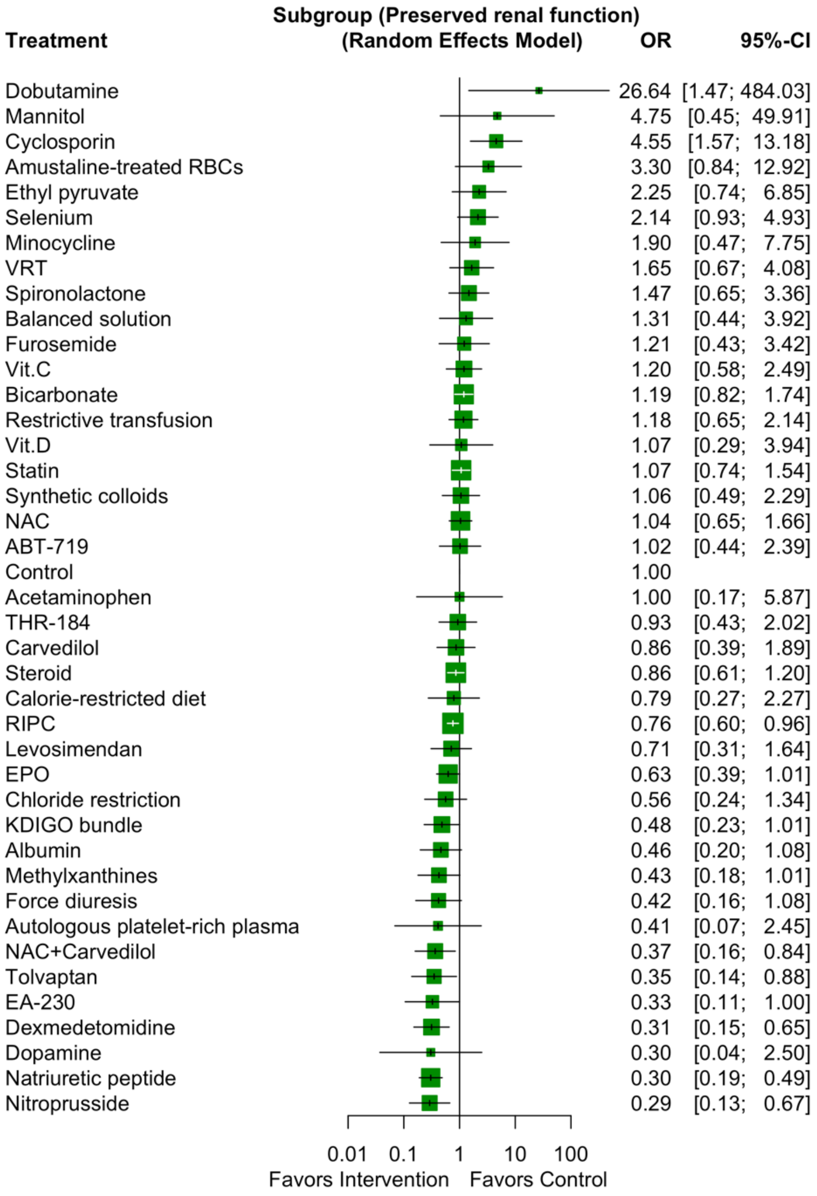

Supplementary Figure 13. Comparison-adjusted funnel plots for AKI prevention in subgroup analysis: preserved renal function (C)

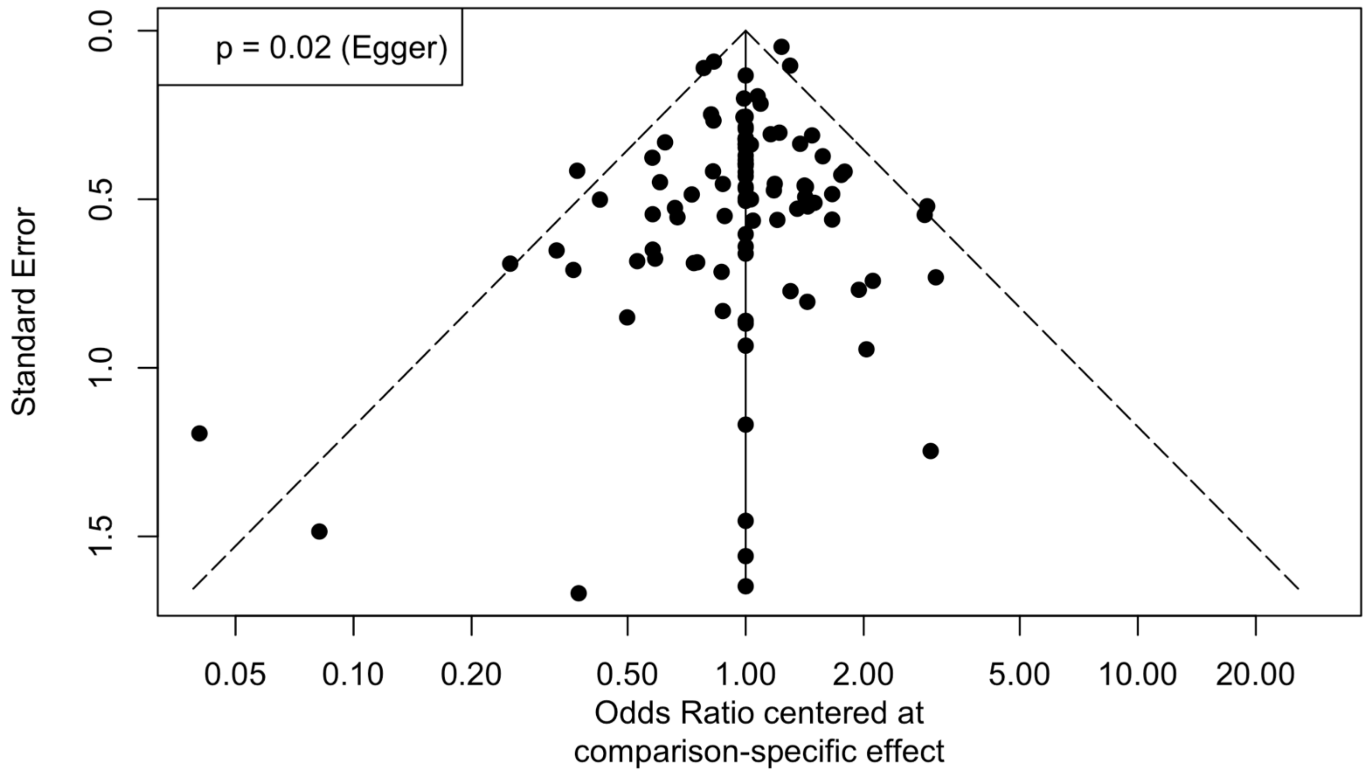

Supplementary Figure 14. Network plot of eligible comparisons among interventions for AKI prevention in subgroup analysis: impaired renal function (A)

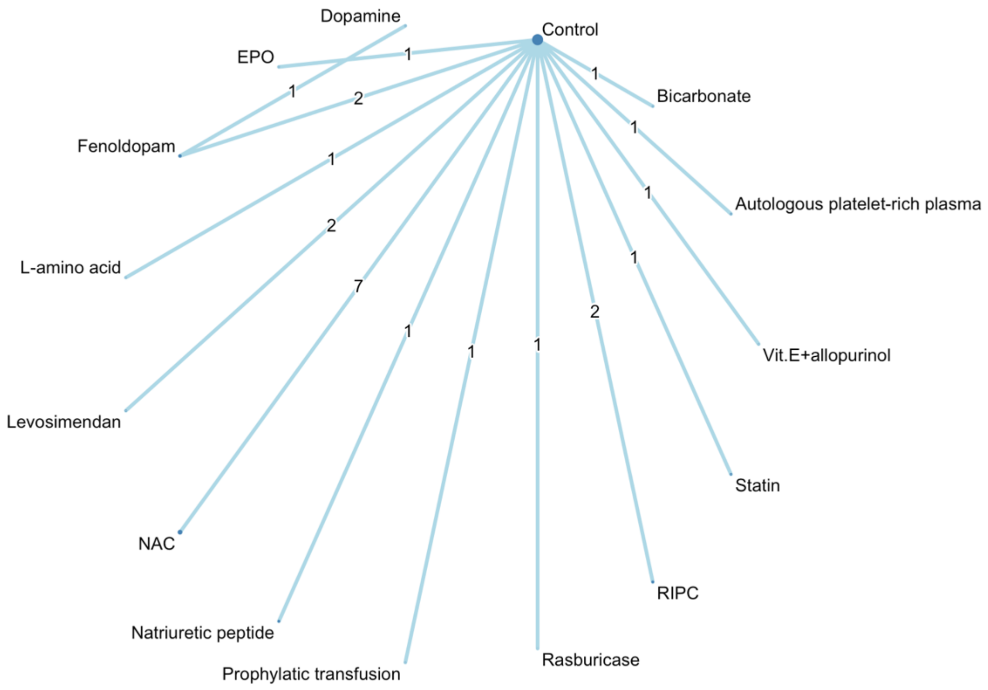

Supplementary Figure 14. Forest plot of network meta-analysis of subgroup analysis: impaired renal function (B)

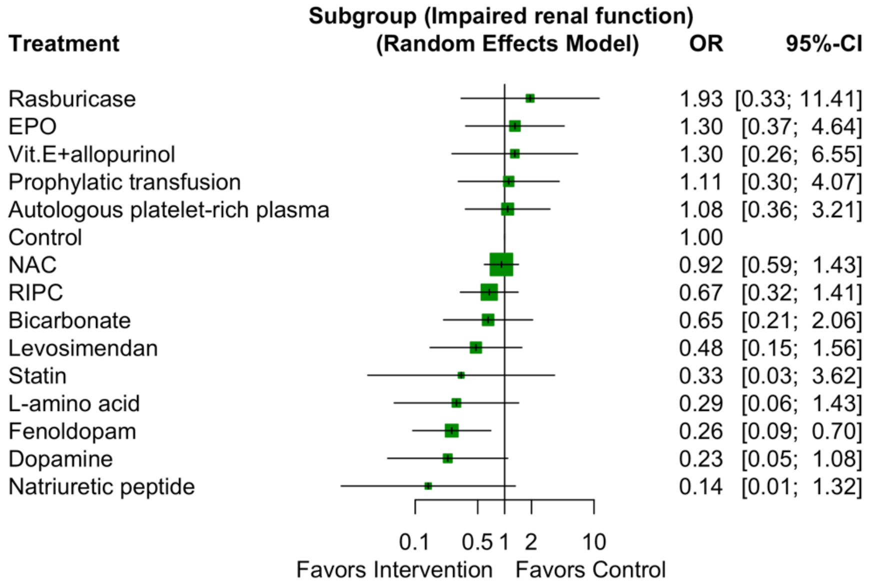

Supplementary Figure 14. Comparison-adjusted funnel plots for AKI prevention in subgroup analysis: impaired renal function (C)

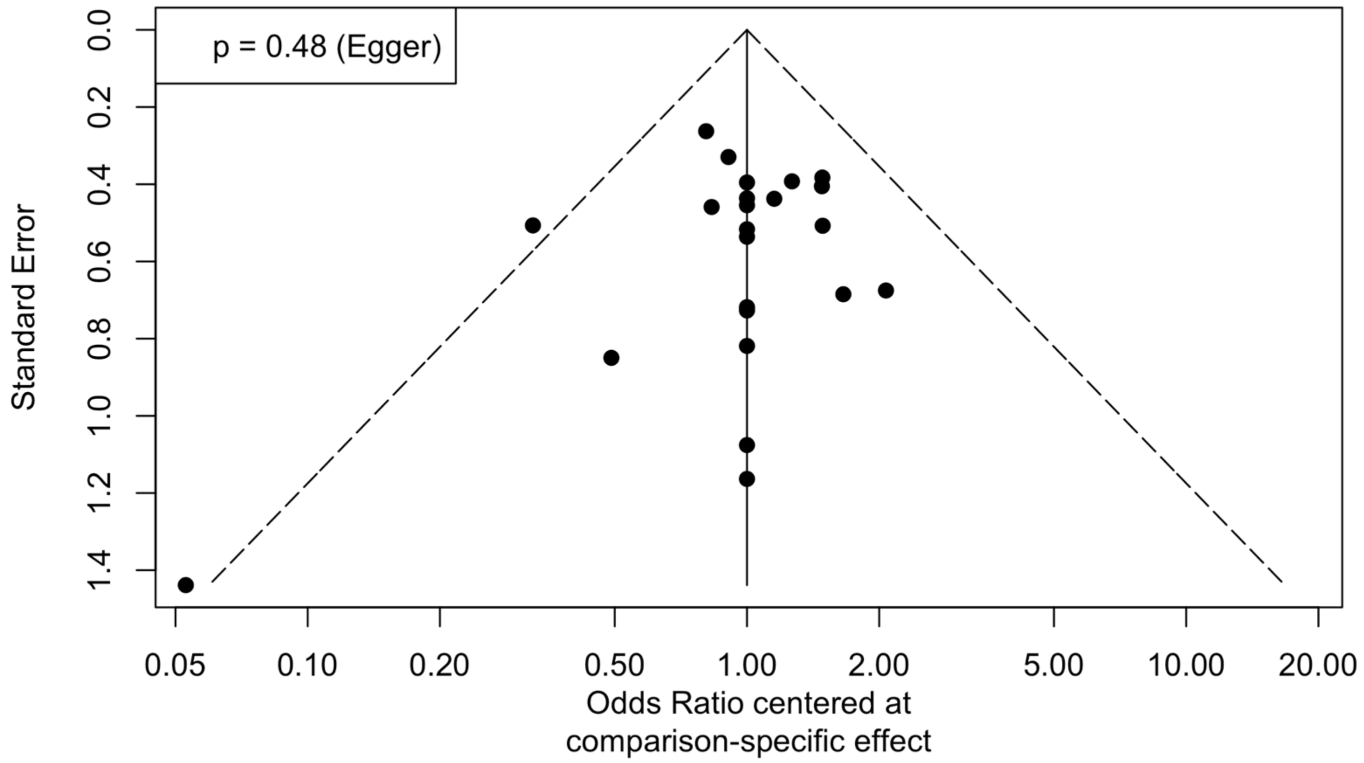

Supplementary Figure 15. Summary of risk of bias.

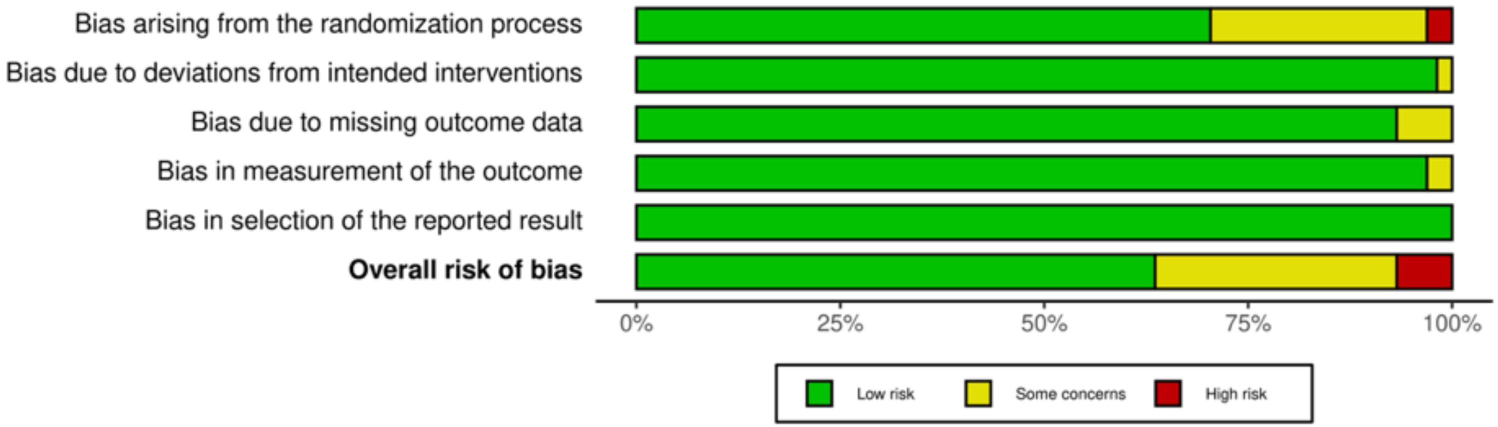

Supplement: Supplementary file 2 [file Data_Sheet_2.PDF]
