# Supplementary material for: Strategies for post–cardiac surgery acute kidney injury prevention: A network meta-analysis of randomized controlled trials
Source: Front Cardiovasc Med. 2022 Sep 27;9:960581. doi: 10.3389/fcvm.2022.960581 (PMC9555275; doi:10.3389/fcvm.2022.960581)
Supplement: Supplementary file 3 [file Data_Sheet_3.docx]

Supplementary Material

**Supplementary Figure Legends**

**Supplementary Figure 1.** PRISMA Flow Diagram

**Supplementary Figure 2.** Comparison-adjusted funnel plots for AKI prevention

**Supplementary Figure 3.** Node-splitting plots for inconsistency tests regarding AKI prevention

**Supplementary Figure 4.** Network plot of eligible comparisons among interventions for dialysis-requiring AKI prevention

**Abbreviation:** EPO: Erythropoietin, NAC: N-acetyl cysteine, RIPC: Remote ischemic preconditioning, VRT: Volume replacement therapy, Vit.C: Vitamin C, Vit.D: Vitamin D

**Supplementary Figure 5.** Network plot of eligible comparisons among interventions for mortality

**Abbreviation:** BS: Balanced solution, Bicar: Bicarbonate, Cont: Control, Cyc: Cyclosporin, Dex: Dexmedetomidine, Dobu:Dobutamine, Dopa: Dopamine, EPO: Erythropoietin, NAC: N-acetyl cysteine, PT: Prophylactic transfusion, RT: Restrictive transfusion, RIPC: Remote ischemic preconditioning, Sele: Selenium, SVV-GFT: Stroke volume variation guided fluid therapy, Vit.C: Vitamin C, Vit.D: Vitamin D,  VRT: Volume replacement therapy

**Supplementary Figure 6.** Network plot of eligible comparisons among interventions for ICU length of stay

**Abbreviation:** BS: Balanced solution, Bicar: Bicarbonate, Cont: Control, Dexm: Dexmedetomidine, Dobu:Dobutamine, Dopa: Dopamine, EPO: Erythropoietin, Methy: Methylxanthines, Mino: Minocycline, NAC: N-acetyl cysteine, NP: Natriuretic peptide, RT: Restrictive transfusion, RIPC: Remote ischemic preconditioning, Sele: Selenium, Spir: Spironolactone, SVV-GFT: Stroke volume variation guided fluid therapy, SC: Synthetic colloids, Vit.C: Vitamin C, Vit.D: Vitamin D , Vit.E: Vitamin E

**Supplementary Figure 7.** Network plot of eligible comparisons among interventions for hospital of stay

**Abbreviation:** EPO: Erythropoietin, NAC: N-acetyl cysteine, RIPC: Remote ischemic preconditioning, Vit.C: Vitamin C, Vit.D: Vitamin D, VRT: Volume replacement therapy

**Supplementary Figure 8.** Network plot of eligible comparisons among interventions for AKI in sensitivity analysis excluding studies without standard AKI criteria

**Abbreviation:** EPO: Erythropoietin, NAC: N-acetyl cysteine, RIPC: Remote ischemic preconditioning, Vit.C: Vitamin C, Vit.D: Vitamin D, VRT: Volume replacement therapy

**Supplementary Figure 9.** Network plot of eligible comparisons among interventions for AKI in sensitivity analysis excluding studies without standard AKI criteria and with risk of bias

**Abbreviation:** EPO: Erythropoietin, RIPC: Remote ischemic preconditioning

**Supplementary Figure 10.** Network plot of eligible comparisons among interventions for AKI in sensitivity analysis excluding studies with small number of participants (A), Forest plot of sensitivity analysis excluding studies with small number of participants (B), Comparison-adjusted funnel plots for sensitivity analysis excluding studies with small number of participants (C)

**Abbreviation:** ABT: ABT-719, Acet: Acetaminophen,  Alb: Albumin, AT-RBCs: Amustaline-treated RBCs, Anti-O: Anti-oxadants, Apr: Aprotinin, APRP: Autologous platelet-rich plasma, BS: Balanced solution, Bica: Bicarbonate, CR-diet: Calorie-restricted diet, Carv: Carvedilol, Cl-Res: Chloride restriction, Cont: Control, Cur: Curcumin, Cyc: Cyclosporin, Dex: Dexmedetomidine, Dobu:Dobutamine, Dopa: Dopamine, EP: Ethyl pyruvate, EPO: Erythropoietin, Feno: Fenoldopam, FD: Forced diuresis, Furo: Furosemide, IIT: Intensive insulin therapy, KB: KDIGO bundle, LAA:L-amino acid, Levo: Levosimendan, Meth: Methylxanthines, NAC: N-acetyl cysteine, NP: Natriuretic peptide, Nitro: Nitroprusside, PT: Prophylactic transfusion, RT: Restrictive transfusion, RIPC: Remote ischemic preconditioning, Sele: Selenium, Spiro: Spironolactone,  Stat: Statin, Ster: Steroid, SVV-GFT: Stroke volume variation guided fluid therapy, SC: Synthetic colloids, THR: THR-184, Vit.C: Vitamin C, Vit.D: Vitamin D , Vit.E+all: Vitamin E + allopurinol , VRT: Volume replacement therapy

**Supplementary Figure 11.** Network plot of eligible comparisons among interventions for AKI prevention in subgroup analysis: heart surgery (A), Comparison-adjusted funnel plots for AKI prevention in subgroup analysis: heart surgery (B)

**Abbreviation:** Aceta: Acetaminophen,  Alb: Albumin, AT-RBCs: Amustaline-treated RBCs, BS: Balanced solution, Bicar: Bicarbonate, Cont: Control, Cyc: Cyclosporin, Dex: Dexmedetomidine, Dobu: Dobutamine, Dopa: Dopamine, EP: Ethyl pyruvate, EPO: Erythropoietin, NAC: N-acetyl cysteine, NP: Natriuretic peptide, PT: Prophylactic transfusion, RT: Restrictive transfusion, RIPC: Remote ischemic preconditioning, Sele: Selenium, SVV-GFT: Stroke volume variation guided fluid therapy, VRT: Volume replacement therapy

**Supplementary Figure 12.** Network plot of eligible comparisons among interventions for AKI prevention in subgroup analysis: aorta surgery (A), Comparison-adjusted funnel plots for AKI prevention in subgroup analysis: aorta surgery (B)

**Abbreviation:** EPO: Erythropoietin,, RIPC: Remote ischemic preconditioning

**Supplementary Figure 13**. Network plot of eligible comparisons among interventions for AKI prevention in subgroup analysis: preserved renal function (A), Forest plot of network meta-analysis of subgroup analysis: preserved renal function (B), Comparison-adjusted funnel plots for AKI prevention in subgroup analysis: preserved renal function (C)

**Supplementary Figure 14.** Network plot of eligible comparisons among interventions for AKI prevention in subgroup analysis: impaired renal function (A), Forest plot of network meta-analysis of subgroup analysis: impaired renal function (B), Comparison-adjusted funnel plots for AKI prevention in subgroup analysis: impaired renal function (C)

**Supplementary Figure 15.** Summary of risk of bias.

**Supplementary Documents**

**Supplementary Document 1.** League table of effect size of different interventions for AKI prevention. Preventive effects are provided as odd ratios and 95% CI. Lower ORs than 1 indicate a clinical significant effect for the column-defining intervention for the network meta-analysis results (lower triangle) and the row-defining intervention for the pair-wise meta-analysis results (upper triangle).

**Supplementary Document 2.** League table of effect size of different interventions for dialysis-requiring AKI prevention. Lower ORs than 1 indicate a clinical significant effect for the column-defining intervention for the network meta-analysis results (lower triangle) and the row-defining intervention for the pair-wise meta-analysis results (upper triangle)

**Supplementary Document 3.** League table of effect size of different interventions for mortality. Lower ORs than 1 indicate a clinical significant effect for the column-defining intervention for the network meta-analysis results (lower triangle) and the row-defining intervention for the pair-wise meta-analysis results (upper triangle)

**Supplementary Document 4.** League table of effect size of different interventions for ICU length of stay. Effects are provided as mean difference and 95% CI. Lower MD than 0 indicate a clinical significant effect for the column-defining intervention for the network meta-analysis results (lower triangle) and the row-defining intervention for the pair-wise meta-analysis results (upper triangle).

**Supplementary Document 5.** League table of effect size of different interventions for hospital of stay. Effects are provided as mean difference and 95% CI. Lower MD than 0 indicate a clinical significant effect for the column-defining intervention for the network meta-analysis results (lower triangle) and the row-defining intervention for the pair-wise meta-analysis results (upper triangle).

**Supplementary Document 6.** Detailed description of risk of bias assessment

**Supplementary Document 7.** Assessment of confidence in NMA (CINeMA) framework

**Supplementary Table Legends**

**Supplementary Table 1.** PRISMA extension checklist of current network meta-analysis

**Supplementary Table 2.** Search strategy for each database

**Supplementary Table 3.** Characteristics of enrolled studies

**Supplementary Table 4.** P-score rank probability matrix of different interventions for AKI prevention

**Supplementary Table 5.** P-score rank probability matrix of different interventions for dialysis-requiring AKI prevention

**Supplementary Table 6.** P-score rank probability matrix of different interventions for mortality

**Supplementary Table 7.** P-score rank probability matrix of different interventions for ICU length of stay

**Supplementary Table 8.** P-score rank probability matrix of different interventions for hospital length of stay

**Supplementary Table 9.** Assessment of risk of bias of including studies

**Supplementary Table 10.** Assessment of confidence of evidence of network meta-analysis of different strategies comparing to control for AKI prevention

**Supplementary Table 11.** Completed but not published trials without available result for post cardiac surgery AKI prevention

**Supplementary Reference**

**Supplementary Reference 151-231**

**Supplementary Document 6. Detailed description of risk of bias assessment**

***Assessment risk of bias of included trials***

Within studies bias were evaluated by the Revised Cochrane Risk-of-Bias tool for randomized trials. Some source of potential bias were identified : (1) not blinded, single blinded owing to preventive strategies in nature (for example: application of KDIGO bundle) and some of enrolled studies did not mention the method for concealing allocation sequence. These studies were considered as some concern regarding to domain 1. Furthermore, 5 studies (Kanchi, 2017; Karkouti, 2012; Pinaud, 2015; Santana-Santos, 2014; Witczak, 2008) were not double blinded in design and have baseline imbalance between groups. These five studies were considered as high concern regarding to domain 1. Eleven studies have some concern regarding to domain 3 due to relative large missing number after participants randomization.  Five studies reported number of acute renal failure/acute kidney injury but did not report pre-specific AKI criteria. These five studies (Cao, 2016; Desai, 2018; Mentzer, 2007; Mohod, 2019; Sezai, 2011) were considered as some concern regarding to domain 4.

**Supplementary Document 7.** Assessment of confidence in NMA (CINeMA) framework

***Grading the evidence of the network meta-analysis using CINeMA***

1. Within-study bias: Overall, 59 of the 161 enrolled studies have high to some concern for within studies bias. The remaining 103 studies were ranked as low concern.

2. Reporting bias: Our search included unpublished study in ClinicalTrials.gov using keywords: “acute kidney injury” AND “cardiac surgery”. Two finished trials which were without available outcome but not published were included. However, overall potential small studies bias were detected by Egger’s test and funnel plot. Therefore, for studies with preventive strategies which was small in enrolled participant number and only examined in single study, we judged this strategy and the study as some concern regarding reporting bias.

3. Indirectness: The indirectness in enrolled studies is judged according to it’s relevance to the research question. We thought all enrolled studies to be no concern in this domain.

4. Imprecision: To evaluate imprecision and heterogeneity, the risk ratio for indicating significant AKI prevention effect was defined as 0.5. Eleven of the 51 strategies were considered as low concern regarding imprecision when comparing with the control group. (see **Supplementary Table 9**).

5. Heterogeneity: The prediction intervals in relation to clinically important effect size were used to judge the variation in treatment effects between enrolled studies. All enrolled strategies were considered as low or some concern except RIPC, which has a prediction interval extending into clinically important effects in both directions.

6. Incoherence: Overall, substantial incoherence was detected. The global incoherence was evaluated using design-by-treatment interaction model. Q value of 17.4 and a *P* value of 0.02 was calculated. Regarding local approach for loop inconsistency, separate Indirect from Direct Evidence (SIDE and node-splitting approach) method was used. When there are both direct and indirect evidence, *P* value from SIDE test was used to judge loop inconsistency.  A *P*  value from SIDE test larger than 0.1 was considered as no concern. For comparisons that had only indirect evidence available, we downgraded the incoherence to some concern levels according to the p-value from  the design by treatment interaction model was less than 0.02. Most comparison was considered as some concern to major concern despite 7 comparison (furosemide vs. control, balanced solution vs. control, dobutamine vs. control, vitamin C vs. control, levosimendan vs. control, NAC vs. control, selenium vs control).

The overall confidence of evidence of each strategy comparing to the control were summarized using the 4 levels of confidence according to the GRADE approach: very low, low, moderate, or high.

**Supplementary Table 1. PRISMA extension checklist of current network meta-analysis**

| Section/Topic | Item | Checklist Item | Page |
| --- | --- | --- | --- |
| **TITLE** |  |  |  |
| Title | 1 | Identify the report as a systematic review *incorporating a network meta-analysis (or related form of meta-analysis).* | 1 |
| **ABSTRACT** |  |  |  |
| Structured summary | 2 | Provide a structured summary including, as applicable:  **Background:** main objectives  **Methods:** data sources; study eligibility criteria, participants, and interventions; study appraisal; and *synthesis methods, such as network meta-analysis.*  **Results:** number of studies and participants identified; summary estimates with corresponding confidence/credible intervals; *treatment rankings may also be discussed. Authors may choose to summarize pairwise comparisons against a chosen treatment included in their analyses for brevity.*  **Discussion/Conclusions:** limitations; conclusions and implications of findings.  **Other:** primary source of funding; systematic review registration number with registry name. | 4 |
| **INTRODUCTION** |  |  |  |
| Rationale | 3 | Describe the rationale for the review in the context of what is already known*, including mention of why a network meta-analysis has been conducted.* | 6 |
| Objectives | 4 | Provide an explicit statement of questions being addressed, with reference to participants, interventions, comparisons, outcomes, and study design (PICOS). | 6 |
| **METHODS** |  |  |  |
| Protocol and registration | 5 | Indicate whether a review protocol exists and if and where it can be accessed (e.g., Web address); and, if available, provide registration information, including registration number. | 7 |
| Eligibility criteria | 6 | Specify study characteristics (e.g., PICOS, length of follow-up) and report characteristics (e.g., years considered, language, publication status) used as criteria for eligibility, giving rationale. *Clearly describe eligible treatments included in the treatment network, and note whether any have been clustered or merged into the same node (with justification).* | 7-8 |
| Information sources | 7 | Describe all information sources (e.g., databases with dates of coverage, contact with study authors to identify additional studies) in the search and date last searched. | 7 |
| Search | 8 | Present full electronic search strategy for at least one database, including any limits used, such that it could be repeated. | 7  Table S2 |
| Study selection | 9 | State the process for selecting studies (i.e., screening, eligibility, included in systematic review, and, if applicable, included in the meta-analysis). | 7-8 |
| Data collection process | 10 | Describe method of data extraction from reports (e.g., piloted forms, independently, in duplicate) and any processes for obtaining and confirming data from investigators. | 8-9 |
| Data items | 11 | List and define all variables for which data were sought (e.g., PICOS, funding sources) and any assumptions and simplifications made. | 8-9 |
| **Geometry of the network** | **S1** | Describe methods used to explore the geometry of the treatment network under study and potential biases related to it. This should include how the evidence base has been graphically summarized for presentation, and what characteristics were compiled and used to describe the evidence base to readers. | 8-9 |
| Risk of bias within individual studies | 12 | Describe methods used for assessing risk of bias of individual studies (including specification of whether this was done at the study or outcome level), and how this information is to be used in any data synthesis. | 11 |
| Summary measures | 13 | State the principal summary measures (e.g., risk ratio, difference in means). *Also describe the use of additional summary measures assessed, such as treatment rankings and surface under the cumulative ranking curve (SUCRA) values, as well as modified approaches used to present summary findings from meta-analyses.* | 9-10 |
| Planned methods of analysis | 14 | Describe the methods of handling data and combining results of studies for each network meta-analysis. This should include, but not be limited to:   - *Handling of multi-arm trials;* - *Selection of variance structure;* - *Selection of prior distributions in Bayesian analyses; and* - *Assessment of model fit.* | 9-10 |
| **Assessment of Inconsistency** | **S2** | Describe the statistical methods used to evaluate the agreement of direct and indirect evidence in the treatment network(s) studied. Describe efforts taken to address its presence when found. | 11 |
| Risk of bias across studies | 15 | Specify any assessment of risk of bias that may affect the cumulative evidence (e.g., publication bias, selective reporting within studies). | 11 |
| Additional analyses | 16 | Describe methods of additional analyses if done, indicating which were pre-specified. This may include, but not be limited to, the following:   - Sensitivity or subgroup analyses; - Meta-regression analyses; - *Alternative formulations of the treatment network; and* - *Use of alternative prior distributions for Bayesian analyses (if applicable).* | 9-10 |
| **RESULTS** |  |  |  |
| Study selection | 17 | Give numbers of studies screened, assessed for eligibility, and included in the review, with reasons for exclusions at each stage, ideally with a flow diagram. | 11-12  Fig S1 |
| **Presentation of network structure** | **S3** | Provide a network graph of the included studies to enable visualization of the geometry of the treatment network. | 11-12  Fig 1 |
| **Summary of network geometry** | **S4** | Provide a brief overview of characteristics of the treatment network. This may include commentary on the abundance of trials and randomized patients for the different interventions and pairwise comparisons in the network, gaps of evidence in the treatment network, and potential biases reflected by the network structure. | 11-12 |
| Study characteristics | 18 | For each study, present characteristics for which data were extracted (e.g., study size, PICOS, follow-up period) and provide the citations. | 12 |
| Risk of bias within studies | 19 | Present data on risk of bias of each study and, if available, any outcome level assessment. | 14 |
| Results of individual studies | 20 | For all outcomes considered (benefits or harms), present, for each study: 1) simple summary data for each intervention group, and 2) effect estimates and confidence intervals. *Modified approaches may be needed to deal with information from larger networks.* | Tab S3 |
| Synthesis of results | 21 | Present results of each meta-analysis done, including confidence/credible intervals. *In larger networks, authors may focus on comparisons versus a particular comparator (e.g. placebo or standard care), with full findings presented in an appendix. League tables and forest plots may be considered to summarize pairwise comparisons.* If additional summary measures were explored (such as treatment rankings), these should also be presented. | 14-17 |
| **Exploration for inconsistency** | **S5** | Describe results from investigations of inconsistency. This may include such information as measures of model fit to compare consistency and inconsistency models, *P* values from statistical tests, or summary of inconsistency estimates from different parts of the treatment network. | 14  Supp  Document |
| Risk of bias across studies | 22 | Present results of any assessment of risk of bias across studies for the evidence base being studied. | 14  Supp  Document |
| Results of additional analyses | 23 | Give results of additional analyses, if done (e.g., sensitivity or subgroup analyses, meta-regression analyses*, alternative network geometries studied, alternative choice of prior distributions for Bayesian analyses,* and so forth). | 16-17 |
| **DISCUSSION** |  |  |  |
| Summary of evidence | 24 | Summarize the main findings, including the strength of evidence for each main outcome; consider their relevance to key groups (e.g., healthcare providers, users, and policy-makers). | 18-19 |
| Limitations | 25 | Discuss limitations at study and outcome level (e.g., risk of bias), and at review level (e.g., incomplete retrieval of identified research, reporting bias). *Comment on the validity of the assumptions, such as transitivity and consistency. Comment on any concerns regarding network geometry (e.g., avoidance of certain comparisons).* | 20 |
| Conclusions | 26 | Provide a general interpretation of the results in the context of other evidence, and implications for future research. | 21 |
| **FUNDING** |  |  |  |
| Funding | 27 | Describe sources of funding for the systematic review and other support (e.g., supply of data); role of funders for the systematic review. This should also include information regarding whether funding has been received from manufacturers of treatments in the network and/or whether some of the authors are content experts with professional conflicts of interest that could affect use of treatments in the network. | 21 |

**Supplementary Table 2.** Search strategy for each database

| No language restrictions were applied. |
| --- |
| **Pubmed through Dec., 10, 2021**  #1 ((((((((((((((Coronary Artery Bypass[MeSH Terms]) OR (Cardiac Surgical Procedures[MeSH Terms])) OR (thoracic surgery[MeSH Terms])) OR (Aorta[MeSH Terms])) OR (Aortic Aneurysm[MeSH Terms])) OR (Coronary Artery Bypass Grafting)) OR (CABG)) OR (coronary bypass grafting surgery)) OR (coronary artery bypass)) OR (cardiac surgery)) OR (heart surgery)) OR (valve replacement)) OR (valve surgery)) OR (aortic dissection surgery)) OR (aortic aneurysm surgery)  #2 ((((Acute Kidney Injury[MeSH Terms]) OR (acute kidney failure)) OR (acute renal failure)) OR (acute kidney injury)) OR (acute kidney insufficiency)  #3 #1 AND #2  #4 #3 Filters: Randomized Controlled Trial, **Result: 490** |
| **EMbase through Dec., 10, 2021**  #1 'acute kidney failure'/exp OR 'acute kidney failure'  #2 'acute kidney injury'/exp OR 'acute kidney injury'  #3 ('heart'/exp OR heart) AND ('surgery'/exp OR surgery)  #4 'coronary artery surgery'/exp OR 'coronary artery surgery'  #5 coronary AND ('bypass'/exp OR bypass) AND ('grafting'/exp OR grafting) AND ('surgery'/exp OR surgery)  #6 'coronary artery bypass graft'/exp OR 'coronary artery bypass graft'  #7 'heart valve replacement'/exp OR 'heart valve replacement'  #8  heart AND valve AND surgery  #9 'aortic surgery'/exp OR 'aortic surgery'  #10 'thoracic surgery'/exp OR 'thoracic surgery'  #11 #3 OR #4 OR #5 OR #6 OR #7 OR #8 OR #9 OR #10  # 12 #1 OR #2  # 13 #11 AND #12  # 14 #13 AND 'randomized controlled trial'/de AND ([adult]/lim OR [aged]/lim), **Result: 656** |
| **Medline through Dec., 10, 2021**  #1 "acute kidney injury".mp. or Acute Kidney Injury/  #2 acute renal insufficiency.mp. or Acute Kidney Injury/  #3 acute renal failure.mp. or Acute Kidney Injury/  #4 cardiac surgery.mp. or Thoracic Surgery/  #5 coronary artery bypass grafting.mp. or Coronary Artery Bypass/  #6 CABG.mp. or Coronary Artery Bypass/  #7 Heart Valve Prosthesis/ or Heart Valve Prosthesis Implantation/  #8 heart valve surgery.mp. or Cardiac Surgical Procedures/  #9 Aortic Aneurysm, Abdominal/ or aortic surgery.mp. or Aortic Aneurysm, Thoracic/ or Vascular Surgical Procedures/  #10 1 and 2 and 3  # 11 4 or 5 or 6 or 7 or 8 or 9  # 12 10 and 11  # 13 12 and "Randomized Controlled Trial".sa_pubt. , **Result: 281** |

**Supplementary Table 3. Characteristics of enrolled studies**

| **Name/Year** | **Country** | **Design** | **Arm** | **Female**  **(%)** | **Age**  **(yr)** | **N** | **AKI definition** | **OP type** | **Intervention** | **Intervention**  **dosage** | **Intervention**  **time** |
| --- | --- | --- | --- | --- | --- | --- | --- | --- | --- | --- | --- |
| Adabag, 2008 | U.S. | Double-blind | 2 | 0.0% | 71.0 | 102 | >0.5 mg/dL or ≥25% increase in Cr by postoperative days 5, 7, and 30 | cardiac | NAC | 14 doses of liquid NAC (3 mL, 600 mg) | pre-OP and post-OP |
| Aldemir, 2016 | Turkey | Double-blind | 2 | 30.0% | 71.0 | 60 | Increase SCr ≥1.5 mg/dL of >25% of the baseline | CABG | NAC | Loading dose of 150 mg/kg over 15 min, followed by 50 mg/kg over the next 4 h and 100 mg/kg over 16 h. | pre-OP, intra-OP and post-OP |
| Ali, 2007 | UK | Double-blind | 2 | 7.0% | 74.5 | 82 | Creatinine>2 mg/dL | Abdominal aorta | RIPC | Sequential crossclamping of the common iliac arteries with 10 minutes ischemia followed by 10 minutes reperfusion | pre-OP |
| Amini, 2018 | Iran | Not blind | 4 | 33.8% | 59.4 | 272 | AKIN | CABG | NAC, vitamin C and selenium | 600 mg/1500 mg/0.5 mg twice daily, day before to 2 days after surgery | pre-OP and post-OP |
| Antonic, 2017 | Slovenia | Not blind | 2 | 20.0% | 64.2 | 100 | AKIN | CABG | Vitamin C | 2 grams of ascorbic acid 24 hours and 2 hours preoperatively and 1 gram twice daily five days after the surgery | pre-OP and post-OP |
| Bagheri, 2018 | Iran | Double-blind | 2 | 42.4% | 63.7 | 177 | AKIN | CABG | RIPC | Three cycles of 5 min ischemia and 5 min reperfusion in the upper arm after induction of anesthesia | pre-OP |
| Balkanay, 2015 | Turkey | Double-blind | 3 | 26.0% | 60.5 | 88 | RIFLE | CABG | Dexmedetomidine | Low-dose (total dose <8 μg/kg) and high-dose Dex (total dose ≥8 μg/kg)  Infusion of Dex at a speed of 0.04 μg/kg/h in the intensive care unit follow-up period. Infusion rates of Dex were regulated to obtain sedation with a Ramsey sedation score of 2 or 3. The infusion dose of Dex (or placebo) increased up to 0.5 μg/kg/h according to the needs of sedation of the patient and was continued for a maximum of 24 hour | pre-OP, intra-OP and post-OP |
| Barba-Navarro, 2017 | Mexico | Not blind | 2 | 41.6% | 53.2 | 233 | KDIGO | Cardiac | Spirolactone | Orally (100 mg 12-24 hours before surgery); subsequently 3 further doses of 25 mg were administered orally on postoperative days 0, 1, and 2 | pre-OP and post-OP |
| Barkhordari, 2011 | Iran | Double-blind | 2 | 17.9% | 58.0 | 28 | 50% or 0.3 mg/dl increase in SCr level | CABG | Pentoxifylline | 5 mg/kg intravenous bolus injection, followed by 1.5 mg/kg/h continuous intravenous infusion until 3 hours after cessation of CPB | pre-OP, intra-OP and post-OP |
| Barr, 2008 | U.S. | Double-blind | 4 | 34.2% | 74.2 | 79 | NA | Cardiac | Fenoldopam/NAC/  Fenoldopam+NAC | Fenoldopam 0.1 μg/ kg/min started at induction and continued for 48 hrs/NAC 600 mg orally twice a day, from preoperative day 1 to postoperative day 1 | pre-OP, intra-OP and post-OP |
| Baysal, 2014 | Turkey | Double-blind | 2 | 52.3% | 57.6 | 128 | NA | Valve | Levosimendan | 6 μg/kg, followed by an infusion (0.1 μg/kg/min) | post-OP |
| Beaver, 2018 | U.S. | Double-blind | 2 | 34.5% | 64.7 | 29 | KDIGO | Thoracic aorta, valve | Nesiritide | 0.01 mcg/kg/min for 48 h | pre-OP |
| Bhaskaran, 2018 | India | Not blind | 2 | 29.5% | 58.8 | 600 | AKIN | CABG | Chloride liberal | Chloride‐rich IVFs : hydroxyethyl starch (130/0.4) in 0.9% normal saline (Voluven) and 0.9% normal saline and ringer lactate VS. Chloride‐restricted IVFs: hydroxyethyl starch (130/0.4) in balanced colloid solution (Volulyte) and balanced salt crystalloid solution (PlasmaLyte A) | pre-OP, intra-OP and post-OP |
| Bennett-Guerrero, 2009 | U.S. | Double-blind | 2 | 37.3% | 75.5 | 102 | RIFLE | Cardiac | Ethyl pyruvate | 7,500 mg administered intravenously starting after the induction followed by 5 more doses of 7,500 mg administered every 6 hours | pre-OP, intra-OP and post-OP |
| Billings IV, 2015 | U.S. | Double-blind | 2 | 40.0% | 62.8 | 60 | AKIN | Cardiac | Acetaminophen | Intravenous, 1g every 6 hours for a weight >50kg (maximum of 4g per 24 hours) or 15mg/kg every 6 hours for a weight <50kg (maximum of 75 mg/ kg per 24 hours) | pre-OP, intra-OP and post-OP |
| Billings IV, 2016 | U.S. | Double-blind | 2 | 30.7% | 66.5 | 615 | AKIN | Cardiac | Atorvastatin | 80mg the day before surgery, 40mg the morning of surgery, and 40mg daily following surgery | pre-OP and post-OP |
| Bove, 2005 | Italy | Double-blind | 2 | 27.5% | 68.5 | 80 | ≥25% increase in Cr | Cardiac | Fenoldopam | 0.05 µg/kg/min | pre-OP |
| Brixner, 2018 | U.S. | Double-blind | 2 | 52.9% | 74.1 | 51 | KDIGO | Cardiac | Amustaline-treated RBCs | NA | intra-OP and post-OP |
| Brulotte, 2013 | Canada | Double-blind | 2 | 17.6% | 73.8 | 34 | AKIN | Thoracic aorta, abdomen aorta | Bicarbonate | Bolus of 3 mL/kg over 1 h, then continued at the rate of 1 mL/kg/h for a total of 6 h after the end of surgery. | pre-OP, intra-OP and post-OP |
| Burns, 2005 | Canada | Quadruple-blind | 2 | 21.0% | 69.0 | 295 | SCr increase of >0.5 mg/dL or 25% from baseline | CABG | NAC | 600 mg 4 dose | intra-OP and post-OP |
| Caimmi, 2003 | Italy | Not blind | 2 | 41.1% | 69.0 | 160 | Increasing of SCr > basal SCr 1.5 times | Cardiac | Fenoldopam | Prophylactic continuous intravenous administration of low-dose fenoldopam (0.1-0.3 µgg/kg/min) during CPB and in the early postoperative period | intra-OP |
| Candilio, 2015 | U.K. | Double-blind | 2 | 21.9% | 65.5 | 178 | SCr rise of >0.3 mg/dL or 150%–200% of baseline and/or urine output <0.5 mL/kg/h for >6 contiguous hours | Cardiac | RIPC | 2–5 min cycles of simultaneous upper arm and thigh cuff inflation/deflation | pre-OP |
| Cao, 2016 | China | Not blind | 2 | 51.7% | 53.0 | 60 | NA | Valve | RIPC | After intubation by three times of inflating the cuff to 200 mmHg for 5 min, followed by 5 min of deflation | pre-OP |
| Carrascal, 2016 | Spanish | Not blind | 2 | 34.4% | 66.5 | 90 | creatinine>2 mg/dL | Cardiac | Atorvastatin | 40 mg/ day of atorvastatin 7 days before and after surgery | pre-OP and post-OP |
| Castillo, 2010 | Chile | Double-blind | 2 | 28.4% | 59.0 | 95 | NA | Cardiac | Anti-oxidant(omega 3 polyunsaturated fatty acids +vit C + Vit E) | omega 3 (2 g ⁄ day), vitamins C (1 g ⁄ day) and E (400 IU ⁄ day) | pre-OP and post-OP |
| Cho, 2016 | Korea | Double-blind | 2 | 52.0% | 63.0 | 200 | AKIN | Cardiac | Dexmedetomidine | 0.4 mg/kg/h starting immediately after anesthetic induction and continuing for 24 h after surgery | pre-OP, intra-OP and post-OP |
| Cho, 2017 | Korea | Double-blind | 2 | 31.4% | 54.5 | 70 | AKIN | Valve | Bicarbonate | 0.5 mmol/kg loading dose for 1 h commencing with anesthetic induction, followed by a 0.15 mmol/kg/h infusion for 23 h | pre-OP, intra-OP and post-OP |
| Cholley, 2017 | France | Double-blind | 2 | 15.8% | 68.0 | 333 | NA | Cardiac | Levosimendan | 0.1μg/kg/min for 24 hours | intra-OP and post-OP |
| Choi, 2011 | Korea | Double-blind | 2 | 60.5% | 58.5 | 76 | AKIN | Valve | RIPC | 3 10-minute cycles of lower limb ischemia and reperfusion with an automated cuff inflator | pre-OP |
| Cogliati, 2007 | Italy | Double-blind | 2 | 35.8% | 69.9 | 193 | Post-OP Cr ≥2 mg/dL + increase in Cr of 0.7 mg/dL from baselinee to maximum postoperative value (day1& day2) | Cardiac | Fenoldopam | 0.1 µg/kg/min | pre-OP |
| Coverdale, 2017 | Canada | Double-blind | 2 | 19.6% | 68.5 | 428 | Increase in SCr of 50% or requirement for dialysis | Aorta, cardiac | RIPC | 3 cycles of 5 minutes of ischemia with the cuff inflated to 200 mm Hg alternated with 5 minutes of cessation of pressure |  |
| Dardashti, 2014 | Sweden | Double-blind | 2 | 21.4% | 72.5 | 70 | RIFLE | CABG | EPO | 400 IU/kg | pre-OP |
| Datzmann, 2018 | Germany | Not blind | 2 | 15.9% | 67.8 | 44 | AKIN | CABG | Starch(6% HES 130/0.4) | NA | NA |
| Desai, 2018 | India | Not blind | 2 | 26.7% | 60.9 | 60 | NA | CABG | Levosimendan | 0.1 μg/kg/min in the previous night before surgery and continued for 24 h including intraoperative period. | pre-OP, intra-OP and post-OP |
| Dieleman, 2012 | Netherlands | Double-blind | 2 | 27.5% | 66.1 | 4482 | RIFLE | Cardiac | Dexamethasone | Single intraoperative dose of 1 mg/kg dexamethasone | intra-OP |
| Duncan, 2020 | U.S. | Double-blind | 2 | 35.5% | 70.0 | 141 | RIFLE | CABG | Starch (Voluven: 6% HES 130/0.4 in 0.9% saline) | Max: 35 ml/kg/day | pre-OP and intra-OP |
| Ederoth, 2018 | U.S. | Double-blind | 2 | 15.6% | 69.0 | 154 | RIFLE | CABG | Cyclosporin | 2.5 mg/kg | pre-OP |
| Erb, 2014 | Switzerland | Double-blind | 2 | 15.2% | 66.5 | 33 | NA | Cardiac | Levosimendan | 12.5 mg, 0.1 mg/kg/min | intra-OP |
| Ejaz, 2009 | U.S. | Double-blind | 2 | 34.0% | 65.0 | 94 | AKIN | Cardiac and thoracic aorta | Nesiritide | 0.01 mg µg/kg/min x 5 days | pre-OP, intra-OP and post-OP |
| Ejaz, 2013 | U.S. | Double-blind | 2 | 30.8% | 63.7 | 26 | AKIN | Cardiac | Rasburicase | 7.5 mg in 50 mL of NS over 30 min | pre-OP |
| Eslami, 2021 | Iran | Double-blind | 2 | 19.8% | 58.0 | 111 | KDIGO | CABG | Vitamin D | 150,000 IU VitD tablets daily for 3 consecutive days | pre-OP |
| Fakhari, 2017 | Iran | Double-blind | 2 | 42.0% | 54.8 | 81 | SCr of >0.5 mg/dL and/or >25% to 50% from baseline | Cardiac | Furosemide | 2 mg/h and continued up to 12 hours postoperatively | pre-OP, intra-OP and post-OP |
| Franco, 2021 | Brazil | Double-blind | 2 | 27.5% | 63.5 | 160 | AKIN | CABG | Dobutamine | Dobutamine to all (starting dose: 5 mcg/kg/min) VS. dobutamine sparing : only in low cardiac output (cardiac index of≤2.4 L/ min/m) | intra-OP and post-OP |
| Gallagher, 2015 | U.K. | Single-blind | 2 | 19.8% | 70.8 | 86 | AKIN | CABG | RIPC | Three 5-minute cycles of forearm ischemia followed by reperfusion | pre-OP |
| Garg, 2018 | Canada | Double-blind | 2 | 17.3% | 76.0 | 606 | KDIGO | Abdominal aorta | Curcumin | 2000-mg doses 8 times over 4 d | pre-OP |
| Garg, 2019 | Canada | Not blind | 2 | 40.6% | 69.7 | 7106 | 0.3 mg/dL or greater or 50% or greater in the 14-day period after surgery, or use of dialysis within 30 days after surgery. | Cardiac | Methylprednisolone | 250 mg at anesthetic induction and 250 mg at initiation of cardiopulmonary bypass | pre-OP |
| Golestaneh, 2014 | U.S. | Double-blind | 2 | 23.7% | 62.5 | 38 | 0.3 mg/dl increase in Cr within 5 days after surgery | CABG | Minocycline | 200 mg initially, then 100 mg every 12 h until surgery | pre-OP |
| Grant, 2008 | U.S. | Double-blind | 2 | NA | NA | 120 | eGFR was less than 75% of baseline and acute renal failure when combined with urine output less than 0.5 mL/kg/hour × 6 hours | CABG | Aprotinin | 10,000 kallikrein inhibiting units (KIU) intravenous test dose was followed by 2 million KIU aprotinin and then 500,000 KIU/hour until the end of the operation. | intra-OP |
| Groenendael, 2021 | Netherlands | Double-blind | 2 | 12.3% | 67.5 | 179 | RIFLE | Cardiac | human chorionic gonadotropin hormone-derivative EA-230 | 90 mg/kg/hr | intra-OP and post-OP |
| Grundmann, 2018 | Germany | Not blind | 2 | 21.1% | 73.6 | 76 | KDIGO | Cardiac | Calorie-restricted diet | 60% of the daily energy expenditure (DEE) from the 7 days before surgery | pre-OP |
| Haase, 2007 | Germany | Double-blind | 2 | 26.7% | 68.6 | 60 | Increase >0.5 mg/dL in Cr | Cardiac | NAC | 150 mg/kg over 15 mins, followed by continuous intravenous infusion of 50 mg/kg in 500 mL over4hrs,then 100mg/kg over 20 hrs→ total 300 mg/kg | pre-OP, intra-OP and post-OP |
| Haase, 2013 | Germany | Double-blind | 2 | 28.9% | 65.5 | 350 | RIFLE | Cardiac | Bicarbonate | 24 hours of intravenous infusion of sodium bicarbonate (5.1 mmol/kg) | pre-OP, intra-OP and post-OP |
| Hausenloy, 2015 | U.K. | Sham-controlled | 2 | 29.2% | 76.2 | 1612 | KDIGO | CABG | RIPC | Upper arm, inflated to 200 mm Hg, and left inflated for 5 minutes. The cuff was then deflated to 0 mm Hg and left uninflated for 5 minutes. This cycle was performed four times in total. | pre-OP |
| Himmelfarb, 2018 | Canada | Sham-controlled | 4 | 36.7% | 74.2 | 297 | KDIGO | Cardiac | THR-184(bone morphogenetic protein-7 agonist) | 10-ml intravenous infusions over 60 minutes. | pre-OP and post-OP |
| Hong, 2012 | Korea | Double-blind | 2 | 27.1% | 64.7 | 70 | Postoperative serum creatinine level >2.0mg/dl accompanied by an increase of >0.7 mg/dl from the preoperative baseline. | CABG | RIPC (Pre+Post) | 4 cycles of 5-min ischemia and 5-min reperfusion in a lower limb using a blood pressure cuff inflated to 200 mmHg. RIPC was applied twice, just after anesthesia induction (RIPC) and just after completion of anastomoses (RIPostC) | pre-OP and post-OP |
| Hong, 2013 | Korea | Double-blind | 2 | 38.7% | 60.8 | 1280 | Postoperative SCr of >2.0 mg/dL + increase of creatinine of at least 0.7 mg/dL from the preoperative baseline | Aorta, cardiac | RIPC (Pre+Post) | The cuff was inflated to 200 mmHg for 5 min and deflated for 5 min. This inflation–deflation cycle was repeated four times. This inflation–deflation protocol was applied twice immediately after induction of anaesthesia before cardiopulmonary bypass (CPB) or coronary anastomoses for RIPC, and immediately after the completion of CPB or coronary anastomoses for RIPostC. | pre-OP and post-OP |
| Hu, 2015 | China | Double-blind | 2 | 62.2% | 47.1 | 201 | AKIN | Valve | RIPC | Three cycles of 5-min ischemiae5-min reperfusion in the right thigh during surgery | intra-OP |
| Hynninen, 2006 | Finland | Double-blind | 2 | 21.7% | 66.5 | 69 | NA | Abdominal aorta | NAC | 150 mg/kg mixed in 250 mL infused in 20 min, followed by an infusion of 150 mg/kg over 24 h | pre-OP, intra-OP and post-OP |
| Jacob, 2015 | Netherlands | Double-blind | 2 | 37.6% | 66.2 | 4465 | RIFLE | Cardiac | Dexamethasone | 1mg/kg | intra-OP |
| Kanchi, 2017 | U.S.. | Not blind | 3 | 5.0% | 60.9 | 60 | KDIGO | CABG | Dopamine | 2 μg/kg/min following anesthesia induction till the end of the surgery and standard care | intra-OP |
| Karkouti, 2012 | Canada | Not blind | 2 | 50.0% | 72.0 | 60 | RIFLE | Cardiac | Prophylactic transfusion | 2 units of erythrocytes transfused 1 to 2 days before surgery | pre-OP |
| Kaya, 2007 | Turkey | Double-blind | 2 | 36.3% | 61.0 | 240 | ≥50% increase in Cr | CABG | Sodium nitroprusside | 0.1 mg/kg/h | intra-OP |
| Krämer, 2002 | Germany | Double-blind | 2 | 25.0% | 60.4 | 56 | Increase of ≥0.4 mg/dl | CABG | Theophylline | 4mg/kr bolus, then 0.25mg/kg/hr | pre-OP, intra-OP and post-OP |
| Kristeller, 2013 | U.S. | Double-blind | 2 | 42.4% | 72.5 | 92 | AKIN | Cardiac | Bicarbonate | 150 mEq mixed in 850 ml of 5% dextrose solution, 3 ml/kg/hr starting 1 hour preoperatively and continuing until the patient was started on CPB | pre-OP |
| Kim, 2012 | Korea | Double-blind | 2 | 44.4% | 57.5 | 54 | Increase in serum creatinine level by either >50% or >0.3 mg/dL from baseline within 48 h after surgery | Thoracic aorta, cardiac | RIPC (Pre+Post) | Three 10-min cycles of right-side lower-limb ischemia, which was induced with an automated cuff inflator placed on the right-side upper leg and inflated to 250 mm Hg, with an intervening 10 min of reperfusion during which the cuff was deflated. RIPCpre plus RIPCpost was performed at 10 min after induction of anesthesia and at 10 min after weaning from CPB | pre-OP and post-OP |
| Kim, 2013 | Korea | Double-blind | 2 | 44.9% | 62.5 | 98 | AKIN | Valve | EPO | 300IU/kg | pre-OP |
| Kim, 2016 | Korea | Double-blind | 2 | 33.3% | 64.5 | 63 | RIFLE | Thoracic aorta | EPO | 500IU/kg | pre-OP |
| Kim, 2017 (a) | Korea | Double-blind | 2 | 19.2% | 66.6 | 120 | AKIN | CABG | Starch (Voluven: 6% HES) | 20ml/kg | intra-OP |
| Kim, 2017 (b) | Korea | Not blind | 2 | 46.9% | 62.3 | 160 | AKIN | Cardiac | RIPC | Cycles of 5 min of ischaemia, which was induced by a blood pressure cuff in the upper arm inflated to 200 mmHg, followed by 5 min of reperfusion, during which the cuff was deflated, 24-48 hours before surgery | pre-OP |
| Kishimoto, 2018 | Japan | Not blind | 2 | 41.1% | 70.2 | 280 | KDIGO | Cardiac | Tolvaptan | Orally at a daily dose of 7.5 mg from POD1 until the time at which the body weight returned to the preoperative level or until POD5. | post-OP |
| Lahtinen, 2011 | Finland | Double-blind | 2 | 30.0% | 69.0 | 200 | Increase in Cr level >50% or greater than two times the upper limit of normal | Cardiac | Levosimendan | 24 μg/kg bolus over 30-mins and thereafter at a dose of 0.2 μg/kg/min | intra-OP and post-OP |
| Landoni, 2017 | Australia | Double-blind | 2 | 35.4% | 66.0 | 506 | RIFLE | Cardiac | Levosimendan | 0.025 to 0.2 μg/kg/min | pre-OP, intra-OP and post-OP |
| Lassnigg, 2000 | Austria | Double-blind | 3 | 27.3% | 63.7 | 123 | increase Cr >0.5 mg/dl | Cardiac | Furosemide/  Dopamine | Furosemide (0.5 μg/kg per min)/dopamine (2 μg/kg per min) | intra-OP and post-OP |
| Lee, 2016 | Korea | Double-blind | 2 | 19.7% | 66.1 | 203 | AKIN/KDIGO | CABG | Albumin | Albumin levels< 4.0 g/dl were administered 100, 200, or 300 ml of 20% human albumin according to the preOP albumin level | pre-OP |
| Lee, 2018 | U.S. | Single-blind | 2 | 46.7% | 64.1 | 30 | AKIN | Cardiac | Lsolyte | Chloride-reduced, acetate-buffered balanced crystalloid: Lsolyte VS. normal saline | pre-OP |
| Levin, 2008 | U.S. | Not blind | 2 | 38.7% | 62.1 | 137 | 50% increase in creatinine vs baseline | CABG | Levosimendan | Loading dose of 10 μg/kg for 1 h, then 0.1 μg/kg/min for 24 h | post-OP |
| Levin, 2012 | U.S. | Double-blind | 2 | 25.4% | 63.3 | 252 | Elevated creatinine >50% from baseline | CABG | Levosimendan | Loading dose 10 μg/kg followed by a 23 h continuous infusion of 0.1μg/kg/min | pre-OP, intra-OP and post-OP |
| Li, 2017 | U.K. | Double-blind | 2 | 30.9% | 67.0 | 285 | KDIGO | Cardiac | Dexmedetomidine | 0.6 μg/kg for 10 mins, then 0.4 μg/kg/hr until the end of surgery. After surgery, 0.1 μg/kg/hr | intra-OP and post-OP |
| Liakopoulos, 2007 | Germany | Not blind | 2 | 29.5% | 66.2 | 78 | Creatinine>2 mg/dL | CABG | Methylprednisolone | Single shot of methylprednisolone (15 mg/kg) or placebo before CPB. | intra-OP |
| Ljunggren, 2019 | Sweden | Double-blind | 2 | 7.5% | 66.5 | 40 | RIFLE | CABG | Mannitol | 1000 mL Ringer's acetate, 10 000 units heparin, and 80 mmol sodium chloride and 200 mL mannitol | intra-OP |
| Lomivorotov, 2014 | Russia | Single-blind | 2 | 15.0% | 58.0 | 40 | KDIGO | CABG | Starch(7.2% NaCl/6% HES 200/0.5 ) | 4ml/kg | pre-OP, intra-OP |
| Lucchinetti, 2012 | Canada | Single-blind | 2 | 9.1% | 60.5 | 55 | AKIN | CABG | RIPC | Four 5-min cycles of lower limb ischemia-reperfusion induced by a tourniquet inflated to 300 mmHg | pre-OP |
| Luckraz, 2021 | U.K. | Not blind | 2 | 22.3% | 67.4 | 220 | RIFLE | Cardiac | Force diuresis | Low- dose (0.25–0.5mg/kg) furosemide along with administration of intravenous (i.v.) fluids at a rate that is matched in real time to the urine output | pre-OP, intra-OP and post-OP |
| Macedo, 2006 | Brazil | Double-blind | 2 | 11.9% | 67.1 | 42 | Increase in Cr≥25% up to the third post- operative day | Thoracic aorta or abdominal aorta | NAC | PO 1200 mg BID 24 h before operation and maintained IV 600mg BID for 48 h after operation | pre-OP and post-OP |
| Mannacio, 2008 | Italy | Double-blind | 2 | 27.5% | 60.3 | 200 | creatinine>2.5 mg/dL | CABG | Rosuvastatin | RSV pretreatment (20 mg/d) starting 7 days before the planned operation | pre-OP |
| McCullough, 2016 | U.S. | Double-blind | 4 | 28.6% | 69.1 | 209 | AKIN | Cardiac | ABT-719(α-melanocyte-stimulating hormone analog) | 800-, 1600-, and 2100µg/kg | pre-OP, intra-OP and post-OP |
| McGuinness, 2013 | New Zealand | Double-blind | 2 | 33.0% | 67.0 | 427 | Increase in creatinine> 25% or 0.5 mg/dL within the post-OP 5 days | Cardiac | Bicarbonate | 0.5 mEq/kg/hr for the first hour and then 0.2 mmol/kg/hr for 23 hours. | pre-OP, intra-OP and post-OP |
| Meersch, 2017 | Germany | Not blind | 2 | 27.9% | 68.4 | 276 | KDIGO | Cardiac | KDIGO bundle | Follow KDIGO guideline | post-OP |
| Meersch, 2020 | Germany | Double-blind | 5 | 33.9% | 69.3 | 100 | KDIGO | Cardiac | RIPC | 3 × 5 min | pre-OP |
| Mehta,2017 | U.S. | Double-blind | 2 | 20.0% | 65.0 | 849 | NA | Cardiac | Levosimendan | 0.2 mcg/kg/min for 1st hour followed by 0.1 mcg/kg for 23 hours | pre-OP, intra-OP and post-OP |
| Mentzer, 2007 | U.S. | Double-blind | 2 | 21.5% | 63.8 | 279 | NA | CABG | Nesiritide | 0.01 μg/kg/min without bolus | pre-OP and post-OP |
| Meybohm, 2013 | Germany | Double-blind | 2 | 18.9% | 69.0 | 180 | AKIN | Cardiac | RIPC | 4 cycles of upper limb ischemia (5-min blood pressure cuff inflation to 200 mmHg, a cuff-pressure at least 15 mm Hg higher than the systolic arterial pressure measured via the arterial line, and 5-min cuff deflation) | pre-OP |
| Meybohm, 2015 | Germany | Double-blind | 2 | 25.8% | 78.2 | 1385 | RIFLE | Cardiac | RIPC | 4 cycles of upper limb ischemia (5-min blood pressure cuff inflation to 200 mmHg, a cuff-pressure at least 15 mm Hg higher than the systolic arterial pressure measured via the arterial line, and 5-min cuff deflation) | NA |
| Mohod, 2019 | India | Not blind | 2 | 12.5% | 55.1 | 40 | NA | CABG | Intensive insulin therapy | Intravenous infusion of 50 units of recombinant human insulin diluted in 50 ml of 0.9% sodium chloride solution, to maintain tight control of blood sugar level between 80 and 110 mg/dl | pre-OP, intra-OP and post-OP |
| Mori, 2014 | Japan | Double-blind | 2 | 33.3% | 74.2 | 42 | AKIN | Thoracic aorta | ANP | 0.0125 mg/kg/min | pre-OP |
| Moriyama, 2017 | Japan | Not blind | 2 | 41.7% | 65.2 | 48 | KDIGO | Cardiac | ANP | 0.025 μg/kg/min | pre-OP, intra-OP and post-OP |
| Moscarelli, 2018 | U.K.. | Double-blind | 2 | 24.2% | 66.4 | 124 | NA | Cardiac | RIPC | Four 5-min cycles of upper limb ischaemia, induced by a blood pressure cuff inflated to 200mmHg, with an intervening 5min of reperfusion by deflating the cuff. | pre-OP |
| Murphy, 2014 | U.K. | Double-blind | 2 | 14.5% | 72.0 | 62 | AKIN | Abdominal aorta | RIPC | 3 cycles of upper arm ischemia. Each cycle was induced by a blood pressure cuff inflated to 100 mmHg above systolic blood pressure for 5 minutes, followed by deflation for a period of 5 minutes | pre-OP |
| Murphy, 2015 | U.K. | Single-blind | 2 | 31.5% | 70.3 | 2003 | AKIN | Cardiac | Restrictive transfusion | Transfuse if haemoglobin falls < 7.5 g/dl | post-OP |
| Nagpal, 2020 | Canada | Double-blind | 2 | 18.8% | 64.5 | 69 | RIFLE | CABG | Starch (Voluven: 6% HES 130/0.4 in 0.9% saline) | Max: 50 mL/kg/d | pre-OP and intra-OP |
| Naguib, 2020 | Egypt | Not blind | 2 | 54.7% | 43.3 | 86 | RIFLE | Valve | Vitamin D | 2 mg PO once daily with food, starting 2 days before cardiac surgery until the end of hospital stay. | pre-OP and post-OP |
| Nouraei, 2016 | Iran | Double-blind | 2 | 29.3% | 60.3 | 99 | AKIN | CABG | RIPC | 3 cycles (each cycle 5-minutes) of a tourniquet cuff inflation applied to upper thigh (≥20mmHg higher than the resting systolic arterial pressure) followed by 5-minute reperfusion. | pre-OP |
| Nouri-Majalan, 2009 | Iran | Double-blind | 2 | 48.3% | 63.0 | 60 | Fall in GFR by 25% | CABG | Vitamin E + allopurinol | 100 units vitamin E four times per day and 100 mg allopurinol twice daily for three to five days prior to elective surgery | pre-OP |
| Oh, 2012 | Korea | Double-blind | 2 | 25.3% | 66.7 | 71 | Increase Cr ≥ 0.3 mg/dL from baseline, ≥ 50% increase Cr in the first 72 hr, or < 0.5 mL/kg/hr of oliguria for >6 hours | CABG | EPO | 300U/kg | pre-OP |
| Ozaydin, 2014 | Turkey | Double-blind | 3 | 26.0% | 63.0 | 296 | Increase in serum creatinine level of >0.5mg/dL or relative increase >25% from baseline level | Cardiac | Carvedilol /Metoprolol | Metoprolol and carvedilol were started at 50 mg once-daily and 6.25 mg twice- daily doses, respectively. The doses were titrated up to maximal tolerated doses. The target doses for metoprolol and carvedilol were 200mg once daily and 25mg twice daily, respectively | pre-OP and post-OP |
| Paparella, 2017 | Italy | Double-blind | 2 | 43.2% | 71.1 | 81 | NA | Cardiac | Methylprednisolone | 250 mg at anaesthetic induction and 250 mg at initiation of CPB | pre-OP |
| Park, 2016 | Korea | Double-blind | 2 | 49.3% | 58.0 | 198 | AKIN | Valve | Atorvastatin | 80 mg single dose on the evening prior to surgery; 40 mg on the morning of surgery; and three further doses of 40 mg on the evenings of postoperative days (POD) 0, 1, and 2 | pre-OP and post-OP |
| Parke, 2015 | New Zealand | Not blind | 2 | 18.8% | 63.2 | 144 | KDIGO | Cardiac | SVV guided bolus fluid strategy | Bolus fluid if inadequte cardiac output and SVV >13 | post-OP |
| Prasad, 2010 | India | Not blind | 2 | 28.3% | 56.7 | 70 | >0.5mg/dL or relative increase >25% from baseline level | CABG | NAC | Oral NAC 600 mg twice a day on the preoperative day and intravenous NAC 600mg on the day of OP and intravenous NAC 600 mg twice a day until the second postoperative day | pre-OP and post-OP |
| Pinaud, 2015 | France | Single-blind | 2 | 48.5% | 74.4 | 99 | AKIN | Valve | RIPC | Three cycles of 5-min inflation to 200 mmHg and 5-min deflation of an automated upper-arm cuff inflator | pre-OP |
| Prowle, 2012 | U.S. | Double-blind | 2 | 30.0% | 68.2 | 85 | RIFLE | Cardiac | Atorvastatin | 40mg/day, 4days before OP | pre-OP |
| Pu, 2018 | Australia | Not blind | 2 | 23.2% | 71.5 | 68 | KDIGO | Cardiac | L-amino acid | 100 g/day | pre-OP, intra-OP and post-OP |
| Rahman, 2010 | U.K.. | Double-blind | 2 | 11.7% | 64.0 | 162 | NA | CABG | RIPC | RIPC (or placebo) stimuli (3 upper limb (or dummy arm), 5-minute cycles of 200 mm Hg cuff inflation/deflation) before aortic clamping | pre-OP |
| Ranucci, 2010 | Italy | Double-blind | 2 | 28.6% | 64.5 | 80 | RIFLE | Cardiac | Fenoldopam | 0.1 µg/kg/min | pre-OP |
| Ristikankare, 2006 | Finland | Double-blind | 2 | 19.5% | 70.5 | 77 | >0.5mg/dL or relative increase >25% from baseline level | Cardiac | NAC | Loading dose of 150 mg/kg in 15 min, followed by 50 mg/kg for the next 4 h, and thereafter, 100 mg/kg for 16 h. | pre-OP, intra-OP and post-OP |
| Ristikankare, 2012 | Finland | Double-blind | 2 | 10.0% | 64.0 | 60 | eGFR decreased more than 25% | CABG | Levosimendan | 12 µg/kg bolus in 10 minutes followed by the infusion of 0.2 µg/kg/min for the next 23 hours and 50 minutes | pre-OP, intra-OP and post-OP |
| Santana-Santos, 2014 | Brazil | Double-blind | 2 | 28.6% | 64.5 | 70 | AKIN | CABG | NAC | 150 mg/kg in 2 hours before surgery, followed by NAC 50 mg/kg over 6 hours | pre-OP, intra-OP and post-OP |
| Sarkar, 2020 | U.K. | Not blind | 2 | 11.9% | 64.0 | 168 | RIFLE | CABG | Preoperative volume replacement therapy | 1 ml/kg/h of Hartmann’s solution for 12 h prior to surgery. | pre-OP |
| Seigneux, 2012 | Switzerland | Double-blind | 3 | 30.0% | 66.3 | 80 | AKIN | Cardiac | EPO | 40000 IU or 20000 IU | post-OP |
| Sezai, 2006 (a) | Japan | Not blind | 2 | 50.0% | 63.4 | 40 | NA | Thoracic aorta | ANP | hANP started at a dose of 0.02μg/kg/min at the start of CPB; the dose was subsequently decreased to 0.01μg/kg/min after the beginning of oral drug administration, and the drug was discontinued 12 h after the beginning of oral drug administration | intra-OP and post-OP |
| Sezai, 2006 (b) | Japan | Double-blind | 2 | 16.2% | 64.3 | 148 | NA | CABG | ANP | hANP started at a dose of 0.02μg/kg/min at the start of CPB; the dose was subsequently decreased to 0.01μg/kg/min after the beginning of oral drug administration, and the drug was discontinued 12 h after the beginning of oral drug administration | intra-OP and post-OP |
| Sezai, 2007 | Japan | Not blind | 2 | 29.8% | 67.3 | 124 | NA | CABG | ANP | hANP started at a dose of 0.02μg/kg/min at the start of CPB; the dose was subsequently decreased to 0.01μg/kg/min after the beginning of oral drug administration, and the drug was discontinued 12 h after the beginning of oral drug administration | intra-OP and post-OP |
| Sezai, 2009 | Japan | Double-blind | 2 | 21.0% | 66.0 | 504 | Increase of ≥0.3 mg/dl | CABG | ANP | hANP 0.02 µg/kg/min initiated at the start of CPB, with the dosage decreased to 0.01 µg/kg/min at the commencement of oral medication and then discontinued after 12 h | pre-OP, intra-OP and post-OP |
| Sezai, 2010 | Japan | Double-blind | 2 | 14.3% | 65.9 | 133 | NA | CABG | ANP | hANP 0.02 µg/kg/min initiated at the start of CPB, with the dosage decreased to 0.01 µg/kg/min at the commencement of oral medication and then discontinued after 12 h | intra-OP and post-OP |
| Sezai, 2011 | Japan | Double-blind | 2 | 11.9% | 68.8 | 285 | NA | CABG | ANP | hANP 0.02 µg/kg/min initiated at the start of CPB, with the dosage decreased to 0.01 µg/kg/min at the commencement of oral medication and then discontinued after 12 h | pre-OP, intra-OP and post-OP |
| Sezai, 2013 | Japan | Double-blind | 2 | 27.2% | 70.5 | 367 | NA | CABG | ANP | No report loading dose,, decreased to 0.01 mg/kg/min at the commencement of oral medication | intra-OP and post-OP |
| Shan, 2021 | China | Double-blind | 2 | 16.3% | 59.6 | 98 | AKIN | Thoracic aorta or abdominal aorta | Dexmedetomidine | 0.4 μg/kg/h immediately after anesthesia induction and 0.1 μg/kg/h after extubation | pre-OP, intra-OP and post-OP |
| Shah, 2014 | India | Double-blind | 2 | 38.0% | 60.6 | 50 | SCr increases >50% | CABG | Levosimendan | 200 μg/kg over 24 h | pre-OP |
| Shahbazi, 2017 | Iran | Double-blind | 2 | 34.7% | 61.3 | 144 | RIFLE | Cardiac | Aminophylline | 5 mg/kg bolus after induction of anesthesia; then, 0.25 mg/kg/hr of the drug was administered intraoperatively and up to 48 hours after surgery | pre-OP, intra-OP and post-OP |
| Sharma, 2013 | India | Double-blind | 2 | 22.5% | 54.3 | 40 | NA | Cardiac | Levosimendan | 200 μg/kg over 24 h | pre-OP |
| Sisillo, 2008 | Italy | Double-blind | 2 | 50.8% | 72.5 | 254 | Increase in serum creatinine x2 | Cardiac | NAC | IV bolus of 1200 mg of NAC before induction of anesthesia, followed by three additional boluses administered at 12-hr intervals in the ICU(total 4800mg) | pre-OP and post-OP |
| Smart, 2021 | Australia | Not blind | 2 | 22.5% | 67.5 | 40 | KDIGO | Cardiac | Succinylated gelatin(4%) | NA | post-OP |
| Smith, 2008 | U.K. | Double-blind | 2 | 27.7% | 74.7 | 47 | NA | Cardiac | Mannitol | 0.5g/kg | intra-OP |
| Soh, 2016 | Korea | Double-blind | 2 | 24.1% | 69.0 | 162 | AKIN | CABG | Bicarbonate | 0.5 mmol/kg for 1 h upon induction of anaesthesia followed by 0.15 mmol kg/h for 23 h | pre-OP, intra-OP and post-OP |
| Soh, 2020 | Korea | Double-blind | 2 | 38.9% | 65.0 | 108 | KDIGO | Thoracic aorta, cardiac | Dexmedetomidine | 0.4 μg/kg/h for 24 h | pre-OP, intra-OP and post-OP |
| Song, 2009 | Korea | Double-blind | 2 | 25.4% | 66.7 | 71 | 50% increase in Cr in 5 days | CABG | EPO | 300U/kg | pre-OP |
| Song, 2015 | Korea | Double-blind | 2 | 29.1% | 68.5 | 117 | Increase SCr ≥ 0.3 mg/dl from baseline, >50% from baseline, or an oliguria < 0.5 ml/kg/h > than 6 hr, within post-OP 48 hr | CABG | NAC | 150 mg/kg bolus, continuous infusion at 150 mg/kg/day for 24 h | pre-OP and intra-OP |
| Song, 2017 | Korea | Double-blind | 2 | 50.0% | 66.5 | 72 | AKIN | Valve | RIPC | 3 cycles of left upper arm ischemia (5-min inflation of the blood pressure cuff to 300 mm Hg followed by 5-min deflation). In the control group, a deflated cuff was placed. | pre-OP |
| Song, 2018 | Korea | Double-blind | 2 | 52.5% | 58.5 | 244 | KDIGO | Valve | RIPC (Pre+Post) | Upon removal of the aortic cross-clamp, three cycles of inflation for 5 min at 250 mm Hg (with 5 min intervals) were applied in the RIC group. Additionally, three cycles of RIC were repeated at postoperative 12 and 24 h. | intra-OP and post-OP |
| Stokfisz, 2020 | Poland | Double-blind | 2 | 42.9% | 66.0 | 28 | KDIGO | CABG | RIPC | 3 cycles of five-minute inflation to 200 mm Hg and five 5-minute deflation of the upper-arm cuff. | pre-OP |
| Swärd, 2004 | Sweden | Double-blind | 2 | 28.8% | 69.7 | 59 | NA | Cardiac | ANP | Continuous infusion at 50 ng/kg/min | NA |
| Tasanarong, 2013 | Thailand | Double-blind | 2 | 43.0% | 61.5 | 100 | KDIGO | CABG | EPO | 200U/kg three days before sugery, then 100U/kg while surgery | pre-OP and intra-OP |
| Thielmann, 2021 | Canada | Double-blind | 2 | 27.6% | 73.3 | 341 | AKIN | Cardiac | Teprasiran(siRNA) | 10 mg/kg dose | post-OP |
| Tian, 2019 | China | Not blind | 2 | 25.8% | 55.0 | 120 | KDIGO | Thoracic aorta | Autologous platelet-rich plasma | 10 to 12 mL/kg | post-OP |
| Tritapepe, 2009 | U.K. | Double-blind | 2 | 19.6% | 66.2 | 102 | creatinine >1.47mg/dL | CABG | Levosimendan | 24 μg/kg | pre-OP |
| Turner, 2014 | U.S. | Not blind | 2 | 37.4% | 70.0 | 123 | Increase in creatinine the first 48 h after surgery of 0.3 mg/dl or more | Cardiac | Bicarbonate | Initial bolus of 0.150 M NaHCO3 at 5.0 ml/kg given prior to initiation of cardio-pulmonary bypass, followed by a 0.150 M NaHCO3 infusion at a rate of 1.0 ml/kg/h for a total of 10h followed by a NaHCO3 infusion of 0.4 ml/kg/h for another 12 h, The goal bicarbonate dose in this study was ∼3.0 mEq/kg. | pre-OP, intra-OP and post-OP |
| Walsh, 2016 | Multi-country | Double-blind | 2 | 41.5% | 72.2 | 258 | AKIN | Cardiac | RIPC | Three 5-minute cycles of thigh ischemia, with 5 minutes of reperfusion be- tween cycles | pre-OP |
| Wijeysundera, 2007 | Canada | Double-blind | 2 | 40.6% | 73.5 | 175 | Increase in creatinine ≥0.5 mg/dl or 25% | Cardiac | NAC | 100 mg/kg iv bolus, 20 mg/kg/hr infusion until four hours after cardiopulmonary bypass | pre-OP, intra-OP and post-OP |
| Witczak, 2008 | Norway | Double-blind | 2 | 20.0% | 66.8 | 20 | NA | Cardiac | Nifedipine | 44.2mg | pre-OP, intra-OP and post-OP |
| Yoo, 2011 | Korea | Single-blind | 2 | 63.5% | 57.5 | 74 | RIFLE | Valve | EPO | 500 IU/kg | pre-OP |
| Young, 2012 | New Zealand | Double-blind | 2 | 37.5% | 65.0 | 96 | RIFLE | Cardiac | RIPC | 3 cycles of 5 min of upper-limb ischemia induced by inflating a blood pressure cuff to 200 mmHg with 5 min of reperfusion | intra-OP |
| Zadeh, 2020 | Iran | Double-blind | 2 | 40.0% | 58.7 | 75 | Two-fold increase in baseline creatinine | CABG | Intensive insulin therapy | If the BS ≥120 mg/dl, an infusion of 50 units of regular insulin in 50 ml saline was started in order to maintain blood glucose between 100 and 120 mg/dl. | intra-OP |
| Zarbock, 2015 | Germany | Double-blind | 2 | 37.1% | 70.4 | 240 | KDIGO | Cardiac | RIPC | 3 cyclesof 5-minute ischemia and 5-minute reperfusion in one upper arm after induction of anesthesia | pre-OP |
| Zarbock, 2021 | Germany | Not blind | 2 | 29.9% | 66.4 | 278 | KDIGO | Cardiac | KDIGO bundle | follow KDIGO guideline | post-OP |
| Zhai, 2017 | China | Double-blind | 2 | 54.2% | 46.0 | 72 | RIFLE | Valve | Dexmedetomidine | 0.6 μg/kg 15 min before anesthesia induction, followed by a treatment of 0.2 μg/kg/h until the end of operation | pre-OP and intra-OP |
| Zheng, 2016 | U.K. | Double-blind | 2 | 20.8% | 59.4 | 1922 | AKIN | Cardiac | Rosuvastatin | 20 mg once daily from 8 days before surgery and for 5 days thereafter | pre-OP and post-OP |
| Zhou, 2015 | U.S. | Double-blind | 2 | 30.0% | 58.5 | 80 | Increase of SCr > 2.0 mg/dL, or twice from baseline, or a new requirement for dialysis | Thoracic aorta | Autologous platelet-rich plasma | 10ml/kg | post-OP |
| Zhou, 2019 | China | Double-blind | 2 | 16.9% | 46.6 | 130 | KDIGO | Thoracic aorta | RIPC | 4 cycles of 5-minute right upper limb ischemia and 5-minute reperfusion | pre-OP |
| Zimmerman, 2011 | U.S. | Single-blind | 2 | 31.4% | 63.5 | 118 | AKIN | Cardiac | RIPC | Three 5-min intervals of lower extremity ischemia separated by 5-min intervals of reperfusion. | pre-OP |
| NCT02668952 | U.S. | Not blind | 2 | 46.7% | 64.1 | 30 | u[TIMP-2][IGFBP7] | Aorta, cardiac | Lsolyte | Lsolyte S injection intravenously as needed. The amount administered (dosage, frequency, and duration) will be left to the clinical judgment of the attending physicians, and will follow usual patterns of use in cardiac surgery patients. | pre-OP, intra-OP and post-OP |
| NCT00484354 | U.S. | Not blind | 2 | 37.4% | 70.0 | 120 | Increase of ≥0.3 mg/dl within 72 hour | CABG | Bicarbonate | Initial bolus of 0.150 M NaHCO3 at 5.0 ml/kg will be given over fifteen minutes prior to surgery. Following the bolus, the 0.150 M NaHCO3 infusion will be run at 1.0 ml/kg/hr during the procedure and for six (6) hours afterwards. The NaHCO3 infusion will then be decreased to 0.4 ml/kg/hr for a further twelve (12) hours. The goal in the NaHCO3 arm is to provide a total dose of approximately 2.5-3.0 mEq/kg of sodium bicarbonate | pre-OP, intra-OP and post-OP |

**Abbreviation:** AKI= Acute kidney injury, AKIN= Acute Kidney Injury Network, ANP= Atrial natriuretic peptide, CABG= Coronary artery bypass graft, EPO= Erythropoietin, KDIGO= Kidney Disease: Improving Global Outcomes, NA= Not available, NAC= N-acetylcysteine, RIFLE= Risk, Injury, Failure, Loss of kidney function, and End-stage kidney disease, RIPC= Remote ischemic preconditioning, SCr= Serum Creatinine

**Supplementary Table 4. P-score rank probability matrix of different interventions for AKI prevention**

| **Intervention** | **P-Score** |
| --- | --- |
| Natriuretic peptide | 0.9170 |
| Nitroprusside | 0.9022 |
| Fenoldopam | 0.8601 |
| EA-230 | 0.8564 |
| L-amino acid | 0.8539 |
| Tolvaptan | 0.8539 |
| NAC+Carvedilol | 0.8497 |
| Methylxanthines | 0.8007 |
| Force diuresis | 0.8000 |
| Dexmedetomidine | 0.7784 |
| Levosimendan | 0.7287 |
| Dopamine | 0.7087 |
| Chloride restriction | 0.7011 |
| Intensive insulin therapy | 0.6987 |
| siRNA | 0.6890 |
| EPO | 0.6816 |
| Synthetic colloids | 0.6261 |
| RIPC | 0.5894 |
| KDIGO bundle | 0.5720 |
| Albumin | 0.5590 |
| Calorie-restricted diet | 0.5524 |
| Vit.D | 0.5201 |
| Steroid | 0.5199 |
| Carvedilol | 0.5124 |
| Autologous platelet-rich plasma | 0.5095 |
| Balanced solution | 0.5057 |
| THR-184 | 0.4783 |
| Anti-oxidant | 0.4758 |
| Acetaminophen | 0.4650 |
| NAC | 0.4517 |
| ABT-719 | 0.4373 |
| Control | 0.4296 |
| Statin | 0.4170 |
| Prophylactic transfusion | 0.4122 |
| Bicarbonate | 0.3763 |
| Vit.C | 0.3715 |
| Vit.E+allopurinol | 0.3689 |
| Restrictive transfusion | 0.3632 |
| Furosemide | 0.3119 |
| Spironolactone | 0.2842 |
| SVV guided fluid therapy | 0.2696 |
| Rasburicase | 0.2584 |
| VRT | 0.2487 |
| Minocycline | 0.2412 |
| Dobutamine | 0.2150 |
| Curcumin | 0.2049 |
| Selenium | 0.1750 |
| Ethyl pyruvate | 0.1749 |
| Aprotinin | 0.1316 |
| Mannitol | 0.1246 |
| Amustaline-treated RBCs | 0.1132 |
| Cyclosporin | 0.0531 |

**Supplementary Table 5. P-score rank probability matrix of different interventions for dialysis-requiring AKI prevention**

| **Intervention** | **P-Score** |
| --- | --- |
| Nifedipine | 0.8491 |
| VRT | 0.8155 |
| ABT-719 | 0.8148 |
| Natriuretic peptide | 0.7969 |
| Dexmedetomidine | 0.7811 |
| Intensive insulin therapy | 0.7760 |
| Carvedilol | 0.6861 |
| NAC+Carvedilol | 0.6845 |
| Force diuresis | 0.6756 |
| Fenoldopam | 0.6346 |
| siRNA | 0.6061 |
| Dopamine | 0.6013 |
| Levosimendan | 0.6011 |
| EPO | 0.5460 |
| Bicarbonate | 0.5058 |
| Autologous platelet-rich plasma | 0.4920 |
| Nitroprusside | 0.4853 |
| Vit.C | 0.4743 |
| Chloride restriction | 0.4742 |
| Aprotinin | 0.4688 |
| Minocycline | 0.4680 |
| Methylxanthines | 0.4676 |
| Mannitol | 0.4675 |
| Fenoldopam+NAC | 0.4606 |
| Dobutamine | 0.4572 |
| Steroid | 0.4554 |
| Prophylactic transfusion | 0.4536 |
| Spironolactone | 0.4458 |
| Vit.D | 0.4417 |
| KDIGO bundle | 0.4348 |
| Control | 0.4340 |
| RIPC | 0.4293 |
| Balanced solution | 0.4099 |
| NAC | 0.4006 |
| Synthetic colloids | 0.3626 |
| Statin | 0.3521 |
| Rasburicase | 0.2661 |
| Furosemide | 0.2368 |
| Albumin | 0.2344 |
| Ethyl pyruvate | 0.2148 |
| Calorie-restricted diet | 0.1737 |
| L-amino acid | 0.1642 |

**Supplementary Table 6. P-score rank probability matrix of different interventions for mortality**

| **Intervention** | **P-Score** |
| --- | --- |
| Minocycline | 0.7989 |
| NAC+Carvedilol | 0.7520 |
| Natriuretic peptide | 0.7305 |
| ABT-719 | 0.7286 |
| Dexmedetomidine | 0.7269 |
| Vit.D | 0.7089 |
| Fenoldopam+NAC | 0.7049 |
| VRT | 0.6936 |
| Cyclosporin | 0.6888 |
| SVV guided fluid therapy | 0.6883 |
| EPO | 0.6846 |
| Rasburicase | 0.6606 |
| Force diuresis | 0.6482 |
| Carvedilol | 0.6142 |
| Levosimendan | 0.6062 |
| siRNA | 0.5477 |
| Steroid | 0.5379 |
| Dopamine | 0.5304 |
| Tolvaptan | 0.5037 |
| Nitroprusside | 0.4993 |
| Intensive insulin therapy | 0.4980 |
| NAC | 0.4932 |
| EA-230 | 0.4883 |
| Mannitol | 0.4865 |
| Aprotinin | 0.4807 |
| Control | 0.4759 |
| Prophylactic transfusion | 0.4695 |
| Calorie-restricted diet | 0.4537 |
| KDIGO bundle | 0.4376 |
| Balanced solution | 0.4354 |
| Bicarbonate | 0.4278 |
| RIPC | 0.4227 |
| Selenium | 0.4089 |
| Spironolactone | 0.3998 |
| Synthetic colloids | 0.3940 |
| Autologous platelet-rich plasma | 0.3873 |
| Fenoldopam | 0.3468 |
| Restrictive transfusion | 0.2894 |
| Vit.C | 0.2740 |
| Dobutamine | 0.1859 |
| Furosemide | 0.1791 |
| Statin | 0.1791 |
| Ethyl pyruvate | 0.1666 |
| Albumin | 0.1656 |

**Supplementary Table 7. P-score rank probability matrix of different interventions for ICU length of stay**

| **Intervention** | **P-Score** |
| --- | --- |
| Fenoldopam | 0.8820 |
| Vit.E+allopurinol | 0.8710 |
| Fenoldopam+NAC | 0.8057 |
| Spironolactone | 0.7916 |
| Vit.D | 0.7534 |
| EPO | 0.7530 |
| Ethyl pyruvate | 0.7484 |
| Autologous platelet-rich plasma | 0.7449 |
| Natriuretic peptide | 0.7316 |
| Levosimendan | 0.6934 |
| Minocycline | 0.6881 |
| Dopamine | 0.5658 |
| Furosemide | 0.5621 |
| Synthetic colloids | 0.5573 |
| Tolvaptan | 0.5374 |
| Methylxanthines | 0.5338 |
| Balanced solution | 0.5336 |
| RIPC | 0.4842 |
| Dexmedetomidine | 0.4484 |
| SVV guided fluid therapy | 0.4148 |
| Calorie-restricted diet | 0.3839 |
| Steroid | 0.3719 |
| EA-230 | 0.3670 |
| Dobutamine | 0.3538 |
| Albumin | 0.3463 |
| Selenium | 0.3402 |
| Control | 0.3258 |
| Vit.C | 0.3052 |
| Restrictive transfusion | 0.2733 |
| Bicarbonate | 0.2602 |
| Statin | 0.2345 |
| Mannitol | 0.1182 |
| NAC | 0.1139 |
| KDIGO bundle | 0.1051 |

**Supplementary Table 8. P-score rank probability matrix of different interventions for hospital length of stay**

| **Intervention** | **P-Score** |
| --- | --- |
| Autologous platelet-rich plasma | 0.9871 |
| Natriuretic peptide | 0.9358 |
| EPO | 0.7867 |
| EA-230 | 0.7757 |
| Vit.D | 0.7403 |
| Dexmedetomidine | 0.7324 |
| Tolvaptan | 0.7233 |
| Fenoldopam+NAC | 0.6849 |
| Levosimendan | 0.6824 |
| Dobutamine | 0.6217 |
| Balanced solution | 0.6187 |
| Steroid | 0.5121 |
| SVV guided fluid therapy | 0.4599 |
| Dopamine | 0.4279 |
| RIPC | 0.4256 |
| Ethyl pyruvate | 0.4011 |
| Statin | 0.3935 |
| Fenoldopam | 0.3775 |
| Calorie-restricted diet | 0.3775 |
| Curcumin | 0.3730 |
| Acetaminophen | 0.3726 |
| Albumin | 0.3721 |
| Restrictive transfusion | 0.3711 |
| KDIGO bundle | 0.3698 |
| Synthetic colloids | 0.3681 |
| Selenium | 0.3577 |
| Control | 0.3511 |
| Vit.C | 0.3176 |
| Bicarbonate | 0.2331 |
| Furosemide | 0.1929 |
| NAC | 0.1567 |

**Supplementary Table 9. Assessment of risk of bias of including studies**

| **Study** | **D1** | **D2** | **D3** | **D4** | **D5** | **Overall** |
| --- | --- | --- | --- | --- | --- | --- |
| Adabag, 2008 | Low | Low | Low | Low | Low | Low |
| Aldemir, 2016 | Low | Low | Low | Low | Low | Low |
| Ali, 2007 | Low | Low | Low | Low | Low | Low |
| Amini, 2018 | Some concerns | Low | Low | Low | Low | Some concerns |
| Antonic, 2017 | Some concerns | Low | Low | Low | Low | Some concerns |
| Bagheri, 2018 | Low | Low | Low | Low | Low | Low |
| Balkanay, 2015 | Low | Low | Low | Low | Low | Low |
| Barba-Navarro, 2017 | Low | Low | Low | Low | Low | Low |
| Barkhordari, 2011 | Low | Low | Low | Low | Low | Low |
| Barr, 2008 | Low | Low | Low | Low | Low | Low |
| Baysal, 2014 | Low | Low | Some concerns | Low | Low | Some concerns |
| Beaver, 2018 | Low | Low | Low | Low | Low | Low |
| Bhaskaran, 2018 | Some concerns | Low | Low | Low | Low | Some concerns |
| Bennett-Guerrero, 2009 | Low | Low | Low | Low | Low | Low |
| Billings IV, 2015 | Low | Low | Some concerns | Low | Low | Some concerns |
| Billings IV, 2016 | Low | Low | Low | Low | Low | Low |
| Bove, 2005 | Low | Low | Low | Low | Low | Low |
| Brixner, 2018 | Low | Low | Some concerns | Low | Low | Some concerns |
| Brulotte, 2013 | Low | Low | Low | Low | Low | Low |
| Burns, 2005 | Low | Low | Low | Low | Low | Low |
| Caimmi, 2003 | Some concerns | Low | Low | Low | Low | Some concerns |
| Candilio, 2015 | Low | Low | Low | Low | Low | Low |
| Cao, 2016 | Some concerns | Low | Low | Some concerns | Low | High |
| Carrascal, 2016 | Some concerns | Low | Low | Low | Low | Some concerns |
| Castillo, 2010 | Low | Low | Some concerns | Low | Low | Some concerns |
| Cho, 2016 | Low | Low | Low | Low | Low | Low |
| Cho, 2017 | Low | Low | Low | Low | Low | Low |
| Cholley, 2017 | Low | Low | Low | Low | Low | Low |
| Choi, 2011 | Low | Low | Low | Low | Low | Low |
| Cogliati, 2007 | Low | Low | Low | Low | Low | Low |
| Coverdale, 2017 | Low | Low | Low | Low | Low | Low |
| Dardashti, 2014 | Low | Low | Low | Low | Low | Low |
| Datzmann, 2018 | Some concerns | Some concerns | Low | Low | Low | High |
| Desai, 2018 | Some concerns | Low | Low | Some concerns | Low | High |
| Dieleman, 2012 | Low | Low | Low | Low | Low | Low |
| Duncan, 2020 | Low | Low | Low | Low | Low | Low |
| Ederoth, 2018 | Low | Low | Low | Low | Low | Low |
| Erb, 2014 | Low | Low | Low | Low | Low | Low |
| Ejaz, 2009 | Low | Low | Low | Low | Low | Low |
| Ejaz, 2013 | Low | Low | Low | Low | Low | Low |
| Eslami, 2021 | Low | Low | Some concerns | Low | Low | Some concerns |
| Fakhari, 2017 | Low | Low | Low | Low | Low | Low |
| Franco, 2021 | Some concerns | Low | Low | Low | Low | Some concerns |
| Gallagher, 2015 | Some concerns | Low | Low | Low | Low | Some concerns |
| Garg, 2018 | Low | Low | Low | Low | Low | Low |
| Garg, 2019 | Some concerns | Some concerns | Low | Low | Low | High |
| Golestaneh, 2014 | Low | Low | Low | Low | Low | Low |
| Grant, 2008 | Low | Low | Low | Low | Low | Low |
| Groenendael, 2021 | Low | Low | Low | Low | Low | Low |
| Grundmann, 2018 | Some concerns | Some concerns | Low | Low | Low | High |
| Haase, 2007 | Low | Low | Low | Low | Low | Low |
| Haase, 2013 | Low | Low | Low | Low | Low | Low |
| Hausenloy, 2015 | Low | Low | Low | Low | Low | Low |
| Himmelfarb, 2018 | Low | Low | Some concerns | Low | Low | Some concerns |
| Hong, 2012 | Low | Low | Low | Low | Low | Low |
| Hong, 2013 | Low | Low | Low | Low | Low | Low |
| Hu, 2015 | Low | Low | Low | Low | Low | Low |
| Hynninen, 2006 | Low | Low | Low | Low | Low | Low |
| Jacob, 2015 | Low | Low | Low | Low | Low | Low |
| Kanchi, 2017 | High | Low | Low | Low | Low | Low |
| Karkouti, 2012 | High | Low | Low | Low | Low | High |
| Kaya, 2007 | Low | Low | Low | Low | Low | Low |
| Krämer, 2002 | Low | Low | Low | Low | Low | Low |
| Kristeller, 2013 | Low | Low | Low | Low | Low | Low |
| Kim, 2012 | Low | Low | Low | Low | Low | Low |
| Kim, 2013 | Low | Low | Low | Low | Low | Low |
| Kim, 2016 | Low | Low | Low | Low | Low | Low |
| Kim, 2017 (a) | Low | Low | Low | Low | Low | Low |
| Kim, 2017 (b) | Low | Low | Low | Low | Low | Low |
| Kishimoto, 2018 | Some concerns | Low | Low | Low | Low | Some concerns |
| Lahtinen, 2011 | Low | Low | Low | Low | Low | Low |
| Landoni, 2017 | Low | Low | Low | Low | Low | Low |
| Lassnigg, 2000 | Low | Low | Low | Low | Low | Low |
| Lee, 2016 | Low | Low | Low | Low | Low | Low |
| Lee, 2018 | Some concerns | Low | Some concerns | Low | Low | High |
| Levin, 2008 | Some concerns | Low | Low | Low | Low | Some concerns |
| Levin, 2012 | Low | Low | Low | Low | Low | Low |
| Li, 2017 | Low | Low | Low | Low | Low | Low |
| Liakopoulos, 2007 | Some concerns | Low | Low | Low | Low | Some concerns |
| Liu, 2016 | Some concerns | Low | Low | Low | Low | Some concerns |
| Ljunggren, 2019 | Low | Low | Low | Low | Low | Low |
| Lomivorotov, 2014 | Some concerns | Low | Low | Low | Low | Some concerns |
| Lucchinetti, 2012 | Low | Low | Low | Low | Low | Low |
| Luckraz, 2021 | Some concerns | Low | Low | Low | Low | Some concerns |
| Macedo, 2006 | Low | Low | Low | Low | Low | Low |
| Mannacio, 2008 | Low | Low | Low | Low | Low | Low |
| McCullough, 2016 | Low | Low | Low | Low | Low | Low |
| McGuinness, 2013 | Low | Low | Low | Low | Low | Low |
| Meersch, 2017 | Some concerns | Low | Low | Low | Low | Some concerns |
| Meersch, 2020 | Low | Low | Low | Low | Low | Low |
| Mehta,2017 | Low | Low | Low | Low | Low | Low |
| Mentzer, 2007 | Low | Low | Low | Some concerns | Low | Some concerns |
| Meybohm, 2013 | Low | Low | Some concerns | Low | Low | Some concerns |
| Meybohm, 2015 | Low | Low | Some concerns | Low | Low | Some concerns |
| Mohod, 2019 | Some concerns | Low | Low | Some concerns | Low | High |
| Mori, 2014 | Low | Low | Some concerns | Low | Low | Some concerns |
| Moriyama, 2017 | Some concerns | Low | Low | Low | Low | Some concerns |
| Moscarelli, 2018 | Low | Low | Low | Low | Low | Low |
| Murphy, 2014 | Low | Low | Low | Low | Low | Low |
| Murphy, 2015 | Some concerns | Low | Low | Low | Low | Some concerns |
| Nagpal, 2020 | Low | Low | Low | Low | Low | Low |
| Naguib, 2020 | Some concerns | Low | Low | Low | Low | Some concerns |
| Nouraei, 2016 | Low | Low | Low | Low | Low | Low |
| Nouri-Majalan, 2009 | Low | Low | Low | Low | Low | Low |
| Oh, 2012 | Low | Low | Low | Low | Low | Low |
| Ozaydin, 2014 | Low | Low | Low | Low | Low | Low |
| Paparella, 2017 | Low | Low | Low | Low | Low | Low |
| Park, 2016 | Low | Low | Low | Low | Low | Low |
| Parke, 2015 | Some concerns | Low | Low | Low | Low | Some concerns |
| Prasad, 2010 | Some concerns | Low | Low | Low | Low | Some concerns |
| Pinaud, 2015 | High | Low | Low | Low | Low | High |
| Prowle, 2012 | Low | Low | Some concerns | Low | Low | Some concerns |
| Pu, 2018 | Some concerns | Low | Low | Low | Low | Some concerns |
| Rahman, 2010 | Low | Low | Low | Low | Low | Low |
| Ranucci, 2010 | Low | Low | Low | Low | Low | Low |
| Ristikankare, 2006 | Low | Low | Low | Low | Low | Low |
| Ristikankare, 2012 | Low | Low | Low | Low | Low | Low |
| Santana-Santos, 2014 | High | Low | Low | Low | Low | High |
| Sarkar, 2020 | Some concerns | Low | Low | Low | Low | Some concerns |
| Seigneux, 2012 | Low | Low | Low | Low | Low | Low |
| Sezai, 2006 (a) | Some concerns | Low | Low | Low | Low | Some concerns |
| Sezai, 2006 (b) | Low | Low | Low | Low | Low | Low |
| Sezai, 2007 | Some concerns | Low | Low | Low | Low | Some concerns |
| Sezai, 2009 | Low | Low | Low | Low | Low | Low |
| Sezai, 2010 | Low | Low | Low | Low | Low | Low |
| Sezai, 2011 | Low | Low | Low | Some concerns | Low | Some concerns |
| Sezai, 2013 | Low | Low | Low | Low | Low | Low |
| Shan, 2021 | Low | Low | Low | Low | Low | Low |
| Shah, 2014 | Low | Low | Low | Low | Low | Low |
| Shahbazi, 2017 | Some concerns | Low | Low | Low | Low | Some concerns |
| Sharma, 2013 | Low | Low | Low | Low | Low | Low |
| Sisillo, 2008 | Low | Low | Low | Low | Low | Low |
| Smart, 2021 | Some concerns | Low | Low | Low | Low | Some concerns |
| Smith, 2008 | Some concerns | Low | Low | Low | Low | Some concerns |
| Soh, 2016 | Low | Low | Low | Low | Low | Low |
| Soh, 2020 | Low | Low | Low | Low | Low | Low |
| Song, 2009 | Low | Low | Low | Low | Low | Low |
| Song, 2015 | Low | Low | Low | Low | Low | Low |
| Song, 2017 | Low | Low | Low | Low | Low | Low |
| Song, 2018 | Low | Low | Low | Low | Low | Low |
| Stokfisz, 2020 | Low | Low | Low | Low | Low | Low |
| Swärd, 2004 | Low | Low | Low | Low | Low | Low |
| Tasanarong, 2013 | Low | Low | Low | Low | Low | Low |
| Thielmann, 2021 | Low | Low | Low | Low | Low | Low |
| Tian, 2019 | Some concerns | Low | Low | Low | Low | Some concerns |
| Tritapepe, 2009 | Low | Low | Low | Low | Low | Low |
| Turner, 2014 | Some concerns | Low | Low | Low | Low | Some concerns |
| Walsh, 2016 | Low | Low | Low | Low | Low | Low |
| Wijeysundera, 2007 | Low | Low | Low | Low | Low | Low |
| Witczak, 2008 | High | Low | Low | Low | Low | High |
| Yoo, 2011 | Some concerns | Low | Low | Low | Low | Some concerns |
| Young, 2012 | Low | Low | Low | Low | Low | Low |
| Zadeh, 2020 | Some concerns | Low | Low | Low | Low | Some concerns |
| Zarbock, 2015 | Low | Low | Low | Low | Low | Low |
| Zarbock, 2021 | Some concerns | Low | Low | Low | Low | Some concerns |
| Zhai, 2017 | Low | Low | Low | Low | Low | Low |
| Zheng, 2016 | Some concerns | Low | Low | Low | Low | Some concerns |
| Zhou, 2015 | Some concerns | Low | Low | Low | Low | Some concerns |
| Zhou, 2019 | Low | Low | Low | Low | Low | Low |
| Zimmerman, 2011 | Some concerns | Low | Low | Low | Low | Some concerns |
| NCT02668952 | Some concerns | Low | Low | Low | Low | Some concerns |
| NCT00484354 | Some concerns | Low | Low | Low | Low | Some concerns |

**Supplementary Table 10. Assessment of confidence of evidence of network meta-analysis of different strategies comparing to control for AKI prevention**

| **Comparison** | **Number of studies** | **Within-study bias** | **Reporting bias** | **Indirectness** | **Imprecision** | **Heterogeneity** | **Incoherence** | **Confidence rating** |
| --- | --- | --- | --- | --- | --- | --- | --- | --- |
| **ABT:Cont** | 1 | No concerns | Low risk | No concerns | Major concerns | No concerns | Some concerns | Low |
| **APRP:Cont** | 2 | Some concerns | Low risk | No concerns | Major concerns | No concerns | Some concerns | Very low |
| **AT-RBC:Cont** | 1 | Some concerns | Some concerns | No concerns | Some concerns | Some concerns | Some concerns | Very low |
| **Acet:Cont** | 1 | Some concerns | Some concerns | No concerns | Major concerns | No concerns | Some concerns | Very low |
| **Alb:Cont** | 1 | No concerns | Low risk | No concerns | Major concerns | No concerns | Some concerns | Low |
| **Anti-O:Cont** | 1 | Some concerns | Some concerns | No concerns | Major concerns | No concerns | Some concerns | Very low |
| **Apro:Cont** | 1 | No concerns | Some concerns | No concerns | Some concerns | No concerns | Some concerns | Low |
| **BS:Cont** | 1 | No concerns | Low risk | No concerns | Major concerns | No concerns | No concerns | Low |
| **Bica:Cont** | 8 | No concerns | Low risk | No concerns | Some concerns | Some concerns | Some concerns | Low |
| **Carv:Cont** | 1 | No concerns | Low risk | No concerns | Major concerns | No concerns | Some concerns | Low |
| **Cl_res:Cont** | 1 | Some concerns | Low risk | No concerns | Some concerns | Some concerns | Some concerns | Low |
| **Cont:CrD** | 1 | Major concerns | Some concerns | No concerns | Major concerns | No concerns | Some concerns | Very low |
| **Cont:Cur** | 1 | No concerns | Low risk | No concerns | Some concerns | No concerns | Some concerns | Moderate |
| **Cont:Cyc** | 1 | No concerns | Some concerns | No concerns | No concerns | No concerns | Some concerns | Low |
| **Cont:Dex** | 7 | No concerns | Low risk | No concerns | No concerns | No concerns | Some concerns | Moderate |
| **Cont:Dobu** | 1 | Some concerns | Low risk | No concerns | Some concerns | Some concerns | No concerns | Low |
| **Cont:Dopa** | 2 | No concerns | Low risk | No concerns | Some concerns | Some concerns | Major concerns | Low |
| **Cont:EA-230** | 1 | No concerns | Some concerns | No concerns | No concerns | Some concerns | Some concerns | Low |
| **Cont:EPO** | 8 | No concerns | Low risk | No concerns | No concerns | Some concerns | Some concerns | Low |
| **Cont:Ep** | 1 | No concerns | Some concerns | No concerns | Major concerns | No concerns | Some concerns | Very low |
| **Cont:FD** | 1 | Some concerns | Low risk | No concerns | Some concerns | No concerns | Some concerns | Low |
| **Cont:Feno** | 3 | No concerns | Low risk | No concerns | No concerns | Some concerns | Major concerns | Low |
| **Cont:Furo** | 2 | No concerns | Some concerns | No concerns | Major concerns | No concerns | No concerns | Very low |
| **Cont:GFT** | 1 | Some concerns | Some concerns | No concerns | Major concerns | No concerns | Some concerns | Very low |
| **Cont:IIT** | 2 | Some concerns | Low risk | No concerns | Major concerns | No concerns | Some concerns | Very low |
| **Cont:KB** | 2 | Some concerns | Low risk | No concerns | Some concerns | Some concerns | Some concerns | Low |
| **Cont:L-AA** | 1 | Some concerns | Some concerns | No concerns | Major concerns | No concerns | Some concerns | Very low |
| **Cont:Levo** | 7 | No concerns | Low risk | No concerns | No concerns | Some concerns | No concerns | Moderate |
| **Cont:Mann** | 1 | No concerns | Some concerns | No concerns | Major concerns | No concerns | Some concerns | Very low |
| **Cont:Meth** | 3 | No concerns | Low risk | No concerns | Some concerns | No concerns | Some concerns | Low |
| **Cont:Mino** | 1 | No concerns | Some concerns | No concerns | Major concerns | No concerns | Some concerns | Very low |
| **Cont:NAC** | 12 | No concerns | Low risk | No concerns | Some concerns | Some concerns | No concerns | Moderate |
| **Cont:NAC+Carv** | 1 | No concerns | Low risk | No concerns | No concerns | Some concerns | Some concerns | Moderate |
| **Cont:NP** | 7 | No concerns | Low risk | No concerns | No concerns | No concerns | Some concerns | Moderate |
| **Cont:Nitr** | 1 | No concerns | Low risk | No concerns | No concerns | No concerns | Some concerns | Moderate |
| **Cont:PT** | 1 | Major concerns | Some concerns | No concerns | Major concerns | No concerns | Some concerns | Very low |
| **Cont:RIPC** | 27 | No concerns | Low risk | No concerns | No concerns | Major concerns | Some concerns | Low |
| **Cont:RT** | 1 | Some concerns | Low risk | No concerns | Major concerns | No concerns | Some concerns | Very low |
| **Cont:Ras** | 1 | No concerns | Some concerns | No concerns | Major concerns | No concerns | Some concerns | Very low |
| **Cont:SC** | 3 | No concerns | Low risk | No concerns | Major concerns | No concerns | Some concerns | Low |
| **Cont:Sele** | 1 | Some concerns | Some concerns | No concerns | Some concerns | No concerns | No concerns | Low |
| **Cont:Spir** | 1 | No concerns | Low risk | No concerns | Major concerns | No concerns | Some concerns | Low |
| **Cont:Stat** | 5 | Some concerns | Low risk | No concerns | Major concerns | No concerns | Some concerns | Very low |
| **Cont:Ster** | 4 | No concerns | Low risk | No concerns | Some concerns | Some concerns | Some concerns | Low |
| **Cont:THR** | 1 | Some concerns | Low risk | No concerns | Major concerns | No concerns | Some concerns | Very low |
| **Cont:Tolv** | 1 | Some concerns | Low risk | No concerns | No concerns | No concerns | Some concerns | Low |
| **Cont:VRT** | 1 | Some concerns | Some concerns | No concerns | Major concerns | No concerns | Some concerns | Very low |
| **Cont:Vit.C** | 2 | Some concerns | Low risk | No concerns | Major concerns | No concerns | No concerns | Very low |
| **Cont:Vit.D** | 2 | Some concerns | Low risk | No concerns | Major concerns | No concerns | Some concerns | Very low |
| **Cont:Vit.E+All** | 1 | No concerns | Some concerns | No concerns | Major concerns | No concerns | Some concerns | Very low |
| **Cont:siRNA** | 1 | No concerns | Some concerns | No concerns | Some concerns | Some concerns | Some concerns | Low |

**Supplementary Table 11. Completed but not published trials without available result for post cardiac surgery AKI prevention**

| **ClinicalTrials.gov Identifier** | **Intervention** | **Comparison** |
| --- | --- | --- |
| NCT04019184 | L-Alanyl/L-Glutamine | Placebo |
| NCT02643745 | Nephrology intervention before surgery | Standard care |
| NCT02610283 | QPI-1002 | Placebo |
| NCT03715868 | Non-milked derived protein source formula diet | Milked derived protein source formula diet |
| NCT02136329 | Sildenafil | Placebo |
| NCT04829916 | RMC-035 | Placebo |
| NCT01547455 | Atorvastatin | Placebo |
| NCT01260259 | RIPC | Control |
| NCT01369732 | EPO | Control |

**Supplementary Reference**

151. Shan XS, Dai HR, Zhao D, Yang BW, Feng XM, Liu H, et al. Dexmedetomidine reduces acute kidney injury after endovascular aortic repair of Stanford type B aortic dissection: A randomized, double-blind, placebo-controlled pilot study. J Clin Anesth. 2021;75:110498.

152. Sezai A, Hata M, Niino T, Yoshitake I, Unosawa S, Wakui S, et al. Results of low-dose human atrial natriuretic peptide infusion in nondialysis patients with chronic kidney disease undergoing coronary artery bypass grafting: the NU-HIT (Nihon University working group study of low-dose HANP Infusion Therapy during cardiac surgery) trial for CKD. J Am Coll Cardiol. 2011;58(9):897-903.

153. Sezai A, Nakata K, Iida M, Yoshitake I, Wakui S, Hata H, et al. Results of low-dose carperitide infusion in high-risk patients undergoing coronary artery bypass grafting. Ann Thorac Surg. 2013;96(1):119-26.

154. Shah B, Sharma P, Brahmbhatt A, Shah R, Rathod B, Shastri N, et al. Study of levosimendan during off-pump coronary artery bypass grafting in patients with LV dysfunction: a double-blind randomized study. Indian J Pharmacol. 2014;46(1):29-34.

155. Shahbazi S, Alishahi P, Asadpour E. Evaluation of the Effect of Aminophylline in Reducing the Incidence of Acute Kidney Injury After Cardiac Surgery. Anesth Pain Med. 2017;7(4):e21740.

156. Sharma P, Malhotra A, Gandhi S, Garg P, Bishnoi A, Gandhi H. Preoperative levosimendan in ischemic mitral valve repair. Asian Cardiovasc Thorac Ann. 2014;22(5):539-45.

157. Sisillo E, Ceriani R, Bortone F, Juliano G, Salvi L, Veglia F, et al. N-acetylcysteine for prevention of acute renal failure in patients with chronic renal insufficiency undergoing cardiac surgery: a prospective, randomized, clinical trial. Crit Care Med. 2008;36(1):81-6.

158. Smart L, Boyd C, Litton E, Pavey W, Vlaskovsky P, Ali U, et al. A randomised controlled trial of succinylated gelatin (4%) fluid on urinary acute kidney injury biomarkers in cardiac surgical patients. Intensive Care Med Exp. 2021;9(1):48.

159. Smith MN, Best D, Sheppard SV, Smith DC. The effect of mannitol on renal function after cardiopulmonary bypass in patients with established renal dysfunction. Anaesthesia. 2008;63(7):701-4.

160. Soh S, Song JW, Shim JK, Kim JH, Kwak YL. Sodium bicarbonate does not prevent postoperative acute kidney injury after off-pump coronary revascularization: a double-blinded randomized controlled trial. Br J Anaesth. 2016;117(4):450-7.

161. Soh S, Shim JK, Song JW, Bae JC, Kwak YL. Effect of dexmedetomidine on acute kidney injury after aortic surgery: a single-centre, placebo-controlled, randomised controlled trial. Br J Anaesth. 2020.

162. Song YR, Lee T, You SJ, Chin HJ, Chae DW, Lim C, et al. Prevention of acute kidney injury by erythropoietin in patients undergoing coronary artery bypass grafting: a pilot study. Am J Nephrol. 2009;30(3):253-60.

163. Song JW, Shim JK, Soh S, Jang J, Kwak YL. Double-blinded, randomized controlled trial of N-acetylcysteine for prevention of acute kidney injury in high risk patients undergoing off-pump coronary artery bypass. Nephrology (Carlton). 2015;20(2):96-102.

164. Song Y, Song JW, Lee S, Jun JH, Kwak YL, Shim JK. Effects of remote ischemic preconditioning in patients with concentric myocardial hypertrophy: A randomized, controlled trial with molecular insights. Int J Cardiol. 2017;249:36-41.

165. Song JW, Lee WK, Lee S, Shim JK, Kim HJ, Kwak YL. Remote ischaemic conditioning for prevention of acute kidney injury after valvular heart surgery: a randomised controlled trial. Br J Anaesth. 2018;121(5):1034-40.

166. Stokfisz K, Ledakowicz-Polak A, Zagorski M, Jander S, Przybylak K, Zielinska M. The clinical utility of remote ischemic preconditioning in protecting against cardiac surgery-associated acute kidney injury: A pilot randomized clinical trial. Adv Clin Exp Med. 2020;29(2):189-96.

167. Sward K, Valsson F, Odencrants P, Samuelsson O, Ricksten SE. Recombinant human atrial natriuretic peptide in ischemic acute renal failure: a randomized placebo-controlled trial. Crit Care Med. 2004;32(6):1310-5.

168. Tasanarong A, Duangchana S, Sumransurp S, Homvises B, Satdhabudha O. Prophylaxis with erythropoietin versus placebo reduces acute kidney injury and neutrophil gelatinase-associated lipocalin in patients undergoing cardiac surgery: a randomized, double-blind controlled trial. BMC Nephrol. 2013;14:136.

169. Thielmann M, Corteville D, Szabo G, Swaminathan M, Lamy A, Lehner LJ, et al. Teprasiran, a Small Interfering RNA, for the Prevention of Acute Kidney Injury in High-Risk Patients Undergoing Cardiac Surgery: A Randomized Clinical Study. Circulation. 2021;144(14):1133-44.

170. Tian WZ, Er JX, Liu L, Chen QL, Han JG. Effects of Autologous Platelet Rich Plasma on Intraoperative Transfusion and Short-Term Outcomes in Total Arch Replacement (Sun's Procedure): A Prospective, Randomized Trial. J Cardiothorac Vasc Anesth. 2019;33(8):2163-9.

171. Tritapepe L, De Santis V, Vitale D, Guarracino F, Pellegrini F, Pietropaoli P, et al. Levosimendan pre-treatment improves outcomes in patients undergoing coronary artery bypass graft surgery. Br J Anaesth. 2009;102(2):198-204.

172. Turner KR, Fisher EC, Hade EM, Houle TT, Rocco MV. The role of perioperative sodium bicarbonate infusion affecting renal function after cardiothoracic surgery. Front Pharmacol. 2014;5:127.

173. Walsh M, Whitlock R, Garg AX, Legare JF, Duncan AE, Zimmerman R, et al. Effects of remote ischemic preconditioning in high-risk patients undergoing cardiac surgery (Remote IMPACT): a randomized controlled trial. CMAJ. 2016;188(5):329-36.

174. Wijeysundera DN, Beattie WS, Rao V, Granton JT, Chan CT. N-acetylcysteine for preventing acute kidney injury in cardiac surgery patients with pre-existing moderate renal insufficiency. Can J Anaesth. 2007;54(11):872-81.

175. Witczak BJ, Hartmann A, Geiran OR, Bugge JF. Renal function after cardiopulmonary bypass surgery in patients with impaired renal function. A randomized study of the effect of nifedipine. Eur J Anaesthesiol. 2008;25(4):319-25.

176. Yoo YC, Shim JK, Kim JC, Jo YY, Lee JH, Kwak YL. Effect of single recombinant human erythropoietin injection on transfusion requirements in preoperatively anemic patients undergoing valvular heart surgery. Anesthesiology. 2011;115(5):929-37.

177. Young PJ, Dalley P, Garden A, Horrocks C, La Flamme A, Mahon B, et al. A pilot study investigating the effects of remote ischemic preconditioning in high-risk cardiac surgery using a randomised controlled double-blind protocol. Basic Res Cardiol. 2012;107(3):256.

178. Javaherforoosh Zadeh F, Azemati S. Adjusted tight control blood glucose management in diabetic patients undergoing on pump coronary artery bypass graft. A randomized clinical trial. J Diabetes Metab Disord. 2020;19(1):423-30.

179. Zarbock A, Schmidt C, Van Aken H, Wempe C, Martens S, Zahn PK, et al. Effect of remote ischemic preconditioning on kidney injury among high-risk patients undergoing cardiac surgery: a randomized clinical trial. JAMA. 2015;313(21):2133-41.

180. Zarbock A, Kullmar M, Ostermann M, Lucchese G, Baig K, Cennamo A, et al. Prevention of Cardiac Surgery-Associated Acute Kidney Injury by Implementing the KDIGO Guidelines in High-Risk Patients Identified by Biomarkers: The PrevAKI-Multicenter Randomized Controlled Trial. Anesth Analg. 2021;133(2):292-302.

181. Zhai M, Kang F, Han M, Huang X, Li J. The effect of dexmedetomidine on renal function in patients undergoing cardiac valve replacement under cardiopulmonary bypass: A double-blind randomized controlled trial. J Clin Anesth. 2017;40:33-8.

182. Zheng Z, Jayaram R, Jiang L, Emberson J, Zhao Y, Li Q, et al. Perioperative Rosuvastatin in Cardiac Surgery. N Engl J Med. 2016;374(18):1744-53.

183. Zhou SF, Estrera AL, Loubser P, Ignacio C, Panthayi S, Miller C, 3rd, et al. Autologous platelet-rich plasma reduces transfusions during ascending aortic arch repair: a prospective, randomized, controlled trial. Ann Thorac Surg. 2015;99(4):1282-90.

184. Zhou H, Yang L, Wang G, Zhang C, Fang Z, Lei G, et al. Remote Ischemic Preconditioning Prevents Postoperative Acute Kidney Injury After Open Total Aortic Arch Replacement: A Double-Blind, Randomized, Sham-Controlled Trial. Anesth Analg. 2019;129(1):287-93.

185. Zimmerman RF, Ezeanuna PU, Kane JC, Cleland CD, Kempananjappa TJ, Lucas FL, et al. Ischemic preconditioning at a remote site prevents acute kidney injury in patients following cardiac surgery. Kidney Int. 2011;80(8):861-7.

186. Mexico UoN. Fluid Chloride and AKI in Cardiopulmonary Bypass: https://ClinicalTrials.gov/show/NCT02668952; 2016 [updated January.

187. Sciences WFUH. Use of Bicarbonate to Reduce the Incidence of Acute Renal Failure After Cardiac Surgery: https://ClinicalTrials.gov/show/NCT00484354; 2006 [updated May.

188. Joosten A, Tircoveanu R, Arend S, Wauthy P, Gottignies P, Van der Linden P. Impact of balanced tetrastarch raw material on perioperative blood loss: a randomized double blind controlled trial. Br J Anaesth. 2016;117(4):442-9.

189. Callum J, Farkouh ME, Scales DC, Heddle NM, Crowther M, Rao V, et al. Effect of Fibrinogen Concentrate vs Cryoprecipitate on Blood Component Transfusion After Cardiac Surgery: The FIBRES Randomized Clinical Trial. JAMA. 2019;322(20):1966-76.

190. Probst S, Cech C, Haentschel D, Scholz M, Ender J. A specialized post anaesthetic care unit improves fast-track management in cardiac surgery: a prospective randomized trial. Crit Care. 2014;18(4):468.

191. Yoo YC, Shim JK, Song Y, Yang SY, Kwak YL. Anesthetics influence the incidence of acute kidney injury following valvular heart surgery. Kidney Int. 2014;86(2):414-22.

192. Demir A, Yilmaz FM, Ceylan C, Doluoglu OG, Ucar P, Zungun C, et al. A comparison of the effects of ketamine and remifentanil on renal functions in coronary artery bypass graft surgery. Ren Fail. 2015;37(5):819-26.

193. van Diepen S, Norris CM, Zheng Y, Nagendran J, Graham MM, Gaete Ortega D, et al. Comparison of Angiotensin-Converting Enzyme Inhibitor and Angiotensin Receptor Blocker Management Strategies Before Cardiac Surgery: A Pilot Randomized Controlled Registry Trial. J Am Heart Assoc. 2018;7(20):e009917.

194. Marathias KP, Vassili M, Robola A, Alivizatos PA, Palatianos GM, Geroulanos S, et al. Preoperative intravenous hydration confers renoprotection in patients with chronic kidney disease undergoing cardiac surgery. Artif Organs. 2006;30(8):615-21.

195. Hajjar LA, Vincent JL, Barbosa Gomes Galas FR, Rhodes A, Landoni G, Osawa EA, et al. Vasopressin versus Norepinephrine in Patients with Vasoplegic Shock after Cardiac Surgery: The VANCS Randomized Controlled Trial. Anesthesiology. 2017;126(1):85-93.

196. Chu DK, Brignardello-Petersen R, Guyatt GH, Ricci C, Genuneit J. Method's corner: Allergist's guide to network meta-analysis. Pediatr Allergy Immunol. 2022;33(1):e13609.

197. Choi MR, Fernandez BE. Protective Renal Effects of Atrial Natriuretic Peptide: Where Are We Now? Front Physiol. 2021;12:680213.

198. Zhao J, Pei L. Cardiac Endocrinology: Heart-Derived Hormones in Physiology and Disease. JACC Basic Transl Sci. 2020;5(9):949-60.

199. Weber AJ, Kail RE, Stanford LR. Morphology of single, physiologically identified retinogeniculate Y-cell axons in the cat following damage to visual cortex at birth. J Comp Neurol. 1989;282(3):446-55.

200. Mitaka C, Ohnuma T, Murayama T, Kunimoto F, Nagashima M, Takei T, et al. Effects of low-dose atrial natriuretic peptide infusion on cardiac surgery-associated acute kidney injury: A multicenter randomized controlled trial. J Crit Care. 2017;38:253-8.

201. Zarbock A, Kellum JA. Remote Ischemic Preconditioning and Protection of the Kidney--A Novel Therapeutic Option. Crit Care Med. 2016;44(3):607-16.

202. Sprick JD, Mallet RT, Przyklenk K, Rickards CA. Ischaemic and hypoxic conditioning: potential for protection of vital organs. Exp Physiol. 2019;104(3):278-94.

203. Yang T, Sun Y, Li Q, Li S, Shi Y, Leak RK, et al. Ischemic preconditioning provides long-lasting neuroprotection against ischemic stroke: The role of Nrf2. Exp Neurol. 2020;325:113142.

204. Johnson ACM, Zager RA. Mechanisms and consequences of oxidant-induced renal preconditioning: an Nrf2-dependent, P21-independent, anti-senescence pathway. Nephrol Dial Transplant. 2018;33(11):1927-41.

205. Yao H, Chi X, Jin Y, Wang Y, Huang P, Wu S, et al. Dexmedetomidine Inhibits TLR4/NF-kappaB Activation and Reduces Acute Kidney Injury after Orthotopic Autologous Liver Transplantation in Rats. Sci Rep. 2015;5:16849.

206. Wang K, Wu M, Xu J, Wu C, Zhang B, Wang G, et al. Effects of dexmedetomidine on perioperative stress, inflammation, and immune function: systematic review and meta-analysis. Br J Anaesth. 2019;123(6):777-94.

207. Liu X, Xie G, Zhang K, Song S, Song F, Jin Y, et al. Dexmedetomidine vs propofol sedation reduces delirium in patients after cardiac surgery: A meta-analysis with trial sequential analysis of randomized controlled trials. J Crit Care. 2017;38:190-6.

208. He H, Peng W, Luan H, Shi C, Tu W. The effect of dexmedetomidine on haemodynamics during intracranial procedures: a meta-analysis. Brain Inj. 2018;32(13-14):1843-8.

209. Toller WG, Stranz C. Levosimendan, a new inotropic and vasodilator agent. Anesthesiology. 2006;104(3):556-69.

210. Bhamidipati CM, LaPar DJ, Stukenborg GJ, Morrison CC, Kern JA, Kron IL, et al. Superiority of moderate control of hyperglycemia to tight control in patients undergoing coronary artery bypass grafting. J Thorac Cardiovasc Surg. 2011;141(2):543-51.

211. Song JW, Shim JK, Yoo KJ, Oh SY, Kwak YL. Impact of intraoperative hyperglycaemia on renal dysfunction after off-pump coronary artery bypass. Interact Cardiovasc Thorac Surg. 2013;17(3):473-8.

212. Umpierrez G, Cardona S, Pasquel F, Jacobs S, Peng L, Unigwe M, et al. Randomized Controlled Trial of Intensive Versus Conservative Glucose Control in Patients Undergoing Coronary Artery Bypass Graft Surgery: GLUCO-CABG Trial. Diabetes Care. 2015;38(9):1665-72.

213. Bonanni A, Signori A, Alicino C, Mannucci I, Grasso MA, Martinelli L, et al. Volatile Anesthetics versus Propofol for Cardiac Surgery with Cardiopulmonary Bypass: Meta-analysis of Randomized Trials. Anesthesiology. 2020;132(6):1429-46.
